# Nuclear Localization and Cleavage of STAT6 Is Induced by Kaposi’s Sarcoma-Associated Herpesvirus for Viral Latency

**DOI:** 10.1371/journal.ppat.1006124

**Published:** 2017-01-18

**Authors:** Chong Wang, Caixia Zhu, Fang Wei, Shujun Gao, Liming Zhang, Yuhong Li, Yanling Feng, Yin Tong, Jianqing Xu, Bin Wang, Zhenghong Yuan, Erle S. Robertson, Qiliang Cai

**Affiliations:** 1 MOE& MOH Key Laboratory of Medical Molecular Virology, School of Basic Medicine, Shanghai Medical College, Fudan University, Shanghai, P. R. China; 2 ShengYushou Center of Cell Biology and Immunology, School of Life Sciences and Biotechnology, Shanghai Jiao Tong University, Shanghai, P. R. China; 3 Hospital and Institute of Obstetrics and Gynecology, Shanghai Medical College, Fudan University, Shanghai, P. R. China; 4 Medical Laboratory, Nanchang Hospital of Integrative Traditional Chinese and Western Medicine, Nanchang, P.R. China; 5 Shanghai Public Health Clinical Center, Fudan University, Shanghai, P. R. China; 6 Division of Hematology, Shanghai First People’s Hospital, Shanghai Jiao Tong University, Shanghai, P. R. China; 7 Department of Microbiology, Perelman School of Medicine, University of Pennsylvania, Philadelphia, Pennsylvania, United States of America; Baylor College of Medicine, UNITED STATES

## Abstract

Emerging evidence implies that STAT6 plays an important role in both the adaptive and innate immune responses to virus infection. Kaposi’s sarcoma-associated herpesvirus (KSHV) is an oncogenic γ-herpesvirus agent associated with several human malignancies, including Kaposi’s sarcoma (KS) and primary effusion lymphomas (PELs). Previously, we demonstrated that KSHV blocks IL-4-induced STAT6 phosphorylation and retains a basal IL-13/STAT6 constitutive activation for cell survival and proliferation. However, the mechanism by which KSHV regulates STAT6 remains largely unknown. Here, we found that KSHV-encoded LANA interacts with STAT6 and promotes nuclear localization of STAT6 independent of the tyrosine 641-phosphorylation state. Moreover, nuclear localization of STAT6 is also dramatically increased in KS tissue. The latent antigen LANA induces serine protease-mediated cleavage of STAT6 in the nucleus, where the cleaved STAT6 lacking transactivation domain functions as a dominant-negative regulator to repress transcription of Replication and Transcription Activator (RTA) and in turn shut off viral lytic replication. Blockade of STAT6 by small interference RNA dramatically enhances expression of RTA, and in turn reduces KSHV-infected endothelial cell growth and colony formation. Taken together, these results suggest that nuclear localization and cleavage of STAT6 is important for modulating the viral latency and pathogenesis of KSHV.

## Author Summary

STAT6, a member of the signal transducer and activator of transcription (STAT) family, has been shown to play an important role in viral infection. STAT6 activation is linked to reactivation of oncogenic herpesvirus and their associated cancers. However, the precise mechanism by which KSHV modulates STAT6 regulation remains unclear. In the present study, we demonstrate that KSHV induces nuclear localization and cleavage of STAT6 in both KSHV-infected B lymphoma and endothelial cells. Importantly, this effect is dependent on LANA (a key latent antigen) expression and leads to inhibition of viral lytic replication. Herein, we provide a previously uncharacterized description of how STAT6 plays an inhibitory role in the pathogenesis of oncogenic viruses.

## Introduction

Signal transducer and activator of transcription (STAT) is a family of latent cytoplasmic transcription factors activated by specific cytokine receptor-mediated signal transducers. Seven members of the STAT family, including STAT1, 2, 3, 4, 5a, 5b, and 6, have been described so far [1]. STAT6 is activated by cytokines like IL-4 and IL-13 that interact with a receptor complex containing IL-4Rα chain [1]. Selective activation of STAT6 by IL-4 or IL-13 involves phosphorylation, dimerization and then translocation into the nucleus, where it binds to specific DNA elements TTC(N_3/4_)GAA within the promoter region, activating gene transcription [2]. It has been demonstrated that STAT6 is required to induce the expression of CD23 and MHC class II, IgE isotype switching in B cells [3], as well as differentiation of Th2 type T cells [4]. However, STAT6 blocks IL-4-dependent inhibition of IFN-γ-induced gene expression in macrophages or Th1 type T-cell differentiation, indicating that STAT6 plays a key role in negative regulation of gene expression [5,6]. Although little is known regarding the mechanisms of down-regulation of STAT6-dependent signaling, recent reports of STAT6 isoform with carboxyl-truncation in both bone marrow-derived mast cells and mast cell lines suggest that STAT6 could function as a dominant-negative regulator in gene expression, which, due to lack of the carboxyl-terminus, interferes with the normal ability of STAT6 to induce transcription of target genes [7,8]. For instance, a 70kDa carboxyl-truncated isoform of STAT6 was detected in IL-4-stimulated mast cells [7], and this cleavage of STAT6 is induced by serine proteases in the nucleus. Interestingly, full length STAT6 (94kDa) can also be cleaved at different sites to yield short STAT6 (60kDa and 55kDa) in the cytoplasm of mast cells by neutrophil elastase and proteinase, respectively [8]–a phenomenon not observed in B cells.

Kaposi’s sarcoma-associated herpesvirus (KSHV), also known as human herpesvirus 8 (HHV-8), is the etiological agent for Kaposi’s sarcoma (KS), and is causally associated with primary effusion lymphoma (PEL) and Multicentric Castleman disease (MCD). Like other herpesviruses, KSHV infection also undergoes a two-stage life cycle: latency and lytic replication. During latency, only a limited number of genes including LANA, vFLIP, vCyclin, kaposin, and the viral microRNAs are expressed [9,10], and play critical roles in cell proliferation, apoptosis, and escape of the host immune surveillance [11,12]. Among these genes, LANA (encoded by ORF73) is the master regulator of KSHV latency. LANA not only functions as a linker to connect KSHV episome with host chromosome for maintenance of KSHV genome [13–15], but also modulates viral and cellular gene expression by interacting with transcription factors and chromatin regulatory proteins. Moreover, LANA modulates the turnover activity of tumor suppressors like p53 and Rb which lead to chromosomal instability [16]. The switch from latency to lytic replication is mediated through another key regulator ― RTA (Replication and Transcription Activator), which is encoded by ORF50 [17]. Upon induction, the mRNA transcript of RTA is expressed and acts as a transcription activator of downstream early and late genes during lytic replication for production of viral progeny [18]. In regard to the role of cytokine signaling in regulation of KSHV-mediated pathogenesis, Very little is known regarding the role of cytogenetic signaling, including STAT signaling, during KSHV latent and lytic replication, although some evidence has indicated that KSHV deregulates cytokine receptor-mediated STAT signal transduction [19–22]. In regards to STAT6, our previous studies have shown that KSHV blocks IL-4-induced STAT6 phosphorylation favoring latency, while stimulation with IL-4 resulted in RTA expression and reactivation of viral lytic replication [22,23]. We recently also found that KSHV retains a basal IL-13/STAT6 constitutive activation for cell survival and proliferation [24]. However, whether STAT6 plays a role in maintaining KSHV latency, independent of IL-4 or IL-13 stimulation remains unclear.

In the present study, we characterized the interaction of STAT6 and LANA, and their roles in KSHV-infected cells with regard to the mechanisms of cleaved STAT6 generation, phosphorylation status and nuclear localization. We identified that cleaved STAT6 lacking transcriptional activity acts as a dominant-negative regulator in lytic gene transcription of KSHV latently-infected cells. Distinct from other STATs, STAT6 cleavage in the KSHV latently-infected cells are due to serine proteases in the nucleus. These findings indicate that nuclear localization and cleavage of STAT6 is critical for KSHV to block lytic replication.

## Results

### Nuclear localization and cleavage of STAT6 is induced by KSHV infection in the latency period

**Fig 1:**
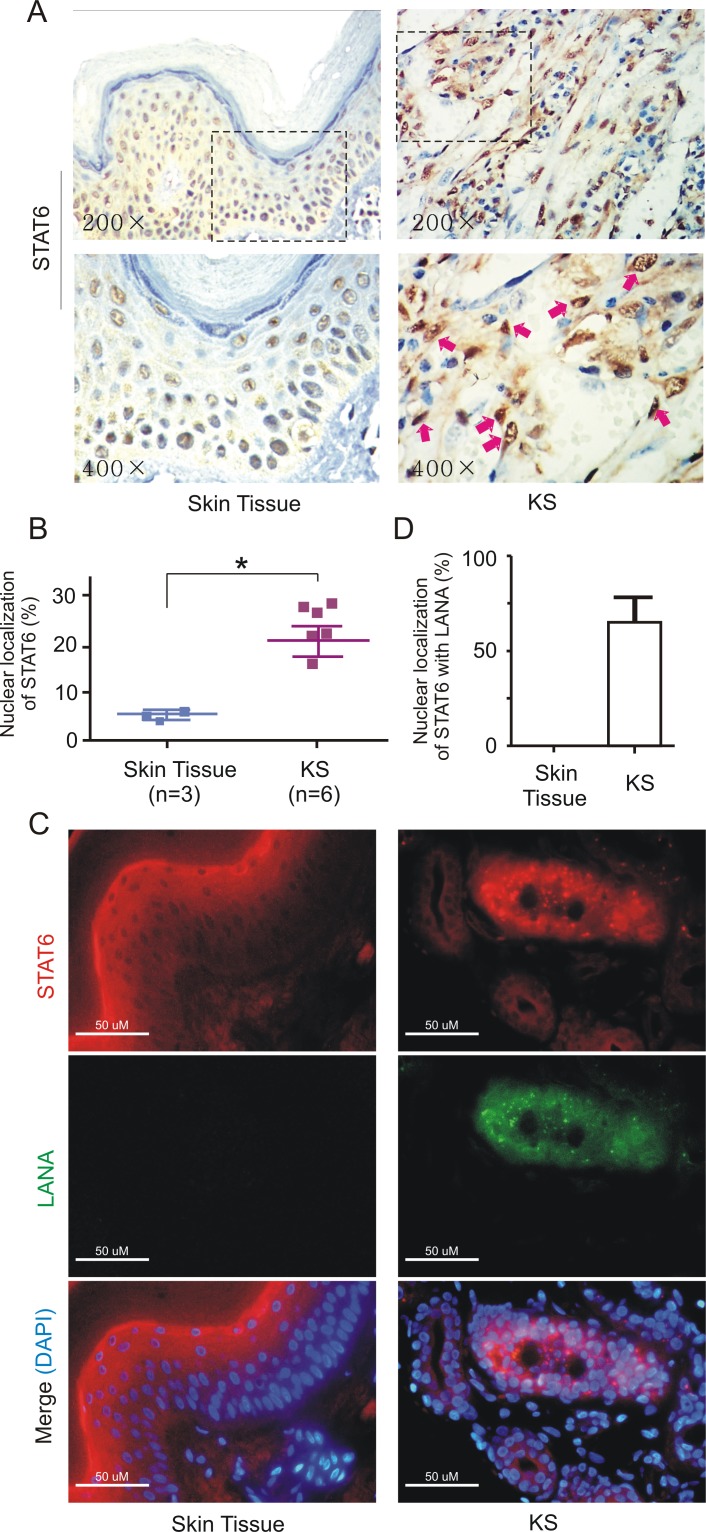
Nuclear localization of STAT6 occurs in KS patient tissues. (**A**) Representative images of immunohistochemistry staining against STAT6 in KS patient tissue and normal skin tissue. The larger magnification (x400) image and relative percentage of staining cells with STAT6 nuclear localization (Arrow) is shown in the lower panels. (**B**) The relative percentage of STAT6 nuclear localization from panel A was individually quantified by nuclear and cytoplasmic staining of 100 cells. Asterisks indicate *P*<0.05. (**C**) Immunofluorescent analysis of endogenous STAT6, LANA and merged in KS patient tissue and normal skin tissue. Nuclei were stained with DAPI. Scale bars represent 50μM. (**D**) The relative percentage of STAT6 nuclear localization in the presence of LANA from panel C was individually quantified by nuclear and cytoplasmic staining of 100 cells.

**Fig 2:**
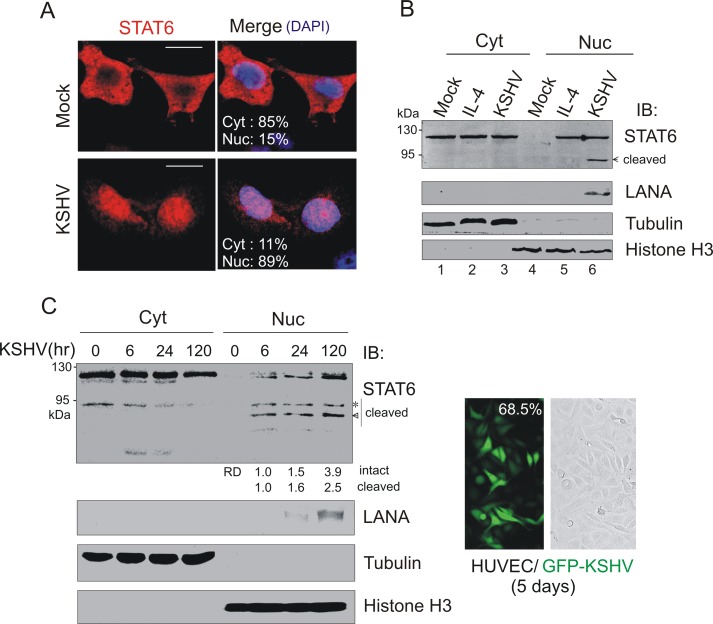
KSHV induces STAT6 nuclear localization. (**A**) Confocal microscopy of endogenous STAT6 in iSLK stable cells with or without KSHV infection after puromycin selection. Nuclei were stained with DAPI. Scale bars represent 10μM. The average percentage of STAT6 localization in nuclear or cytoplasm compartment was calculated based on observation of fifty-staining cells. (**B**) Western blot analyses of fractionated proteins from panel A. iSLK cells treated with IL-4 (50 ng/ml) for 30 min were used as positive control for STAT6 nuclear localization. Cyt, cytosolic; Nuc, nuclear, were revealed by α-tubulin, Histone H3, and LANA, respectively. Arrow indicates the major band of cleaved STAT6 induced by KSHV. (**C**) Western blot analyses of fractionated proteins from HUVEC cells with KSHV primary infection. Asterisk is a specific band of cleaved STAT6 existed in HUVEC cells. The relative density (RD) of intact and cleaved STAT6 proteins in nuclear compartment was quantitated and shown. The average percentage of HUVEC cells with KSHV infection (GFP positive) at 5 days is shown in right panel.

Previously, despite the absence of detectable phosphorylation of Y641 on STAT6 in KS tissue [24], we clearly observed there were more nuclear localization of total STAT6 in KS tissues than in normal skin controls (Fig 1A and 1B), which is further verified by the immunofluorescence assays against STAT6 and LANA (Fig 1C and 1D). To further confirm if infection of KSHV enhances nuclear localization of STAT6, endogenous STAT6 in iSLK cells with and without KSHV infection were monitored by immunofluorescence and cell fractionation assay. As shown in Fig 2A and 2B, there was an increase in the amount of nuclear localization of STAT6 in KSHV-infected cells when compared with control cells. Moreover, in HUVEC cells with KSHV primary infection, more STAT6 consistently translocated into nuclear compartment upon KSHV infection (Fig 2C), further supporting the hypothesis that KSHV induces STAT6 nuclear translocation. Interestingly, we also found a cleaved isoform of STAT6 present in the nuclear compartment of both iSLK and HUVEC cells upon KSHV infection, although there was additional slow-migrated cleaved isoform of STAT6 presented in HUVEC cells (Fig 2B and 2C).

**Fig 3:**
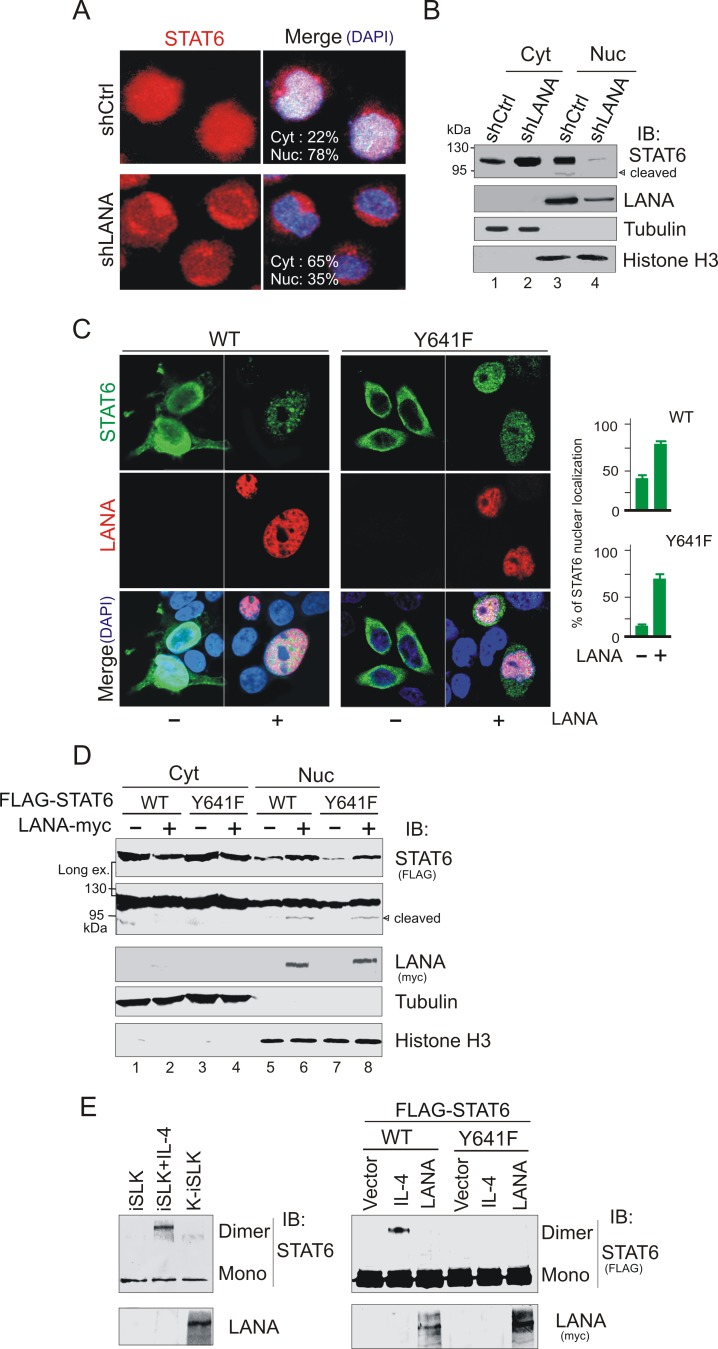
LANA promotes nuclear localization of STAT6 independent of Y641-phosphorylation. (**A**) Inhibition of LANA blocks nuclear localization of STAT6. Confocal microscopy of endogenous STAT6 (red) and merged in PEL cells BC3 with lentivirus-mediated constitutively knocked down of LANA (shLANA) or luciferase control (shCtrl). Nuclei were stained with DAPI. (**B**) Western blot analyses of fractionated BC3 cells with LANA or luciferase control knockdown. Cyt, cytosolic; Nuc, nuclear; were revealed by α-tubulin and Histone H3, respectively. (**C**) LANA induces nuclear localization of exogenous STAT6 independent of phosphorylation on tyrosine 641. HEK293 cells transfected with wild type (WT) or Y641 mutant (Y641F) of STAT6 with FLAG tag (green) in the presence or absence of LANA with RFP tag (red) were imaged by confocal assay. Nuclei were stained with DAPI. The relative percentage of STAT6 nuclear localization (right panel) was individually quantitated by nuclear and cytoplasmic staining of 100 cells. (**D**) Immunoblotting analyses of fractionated 293 cells transfected with expressing plasmids as indicated. Cyt, cytosolic; Nuc, nuclear; were revealed by α-tubulin and Histone H3, respectively. (**E**) Cells from panel C in Fig 1 or panel D were individually treated with DSS for 30 minutes before lysis and analyzed by immunoblotting (IB) for dimerization of STAT6 (data is shown on the left and right panels, respectively). Cells treated with IL-4 (50 ng/ml) for 30 min were used as control.

To further investigate if there was a similar effect STAT6 localization and cleavage in B cells, and address whether KSHV-encoded latent antigen LANA contributes to nuclear localization of STAT6, we performed immunofluorescence assays of KSHV-infected B lymphoma PEL cells with or without LANA knockdown. As shown in Fig 3A, we observed that inhibition of LANA dramatically reduced nuclear localization of STAT6 (from 78% to 35%) in BC3 cells. Western blot analysis of nuclear and cytoplasm fractionation further confirmed the role of LANA in induction of STAT6 nuclear localization and cleavage (Fig 3B), indicating that nuclear localization and cleavage of STAT6 is induced by LANA in response to KSHV latent infection.

### Nuclear localization and cleavage of STAT6 is due to interaction with LANA and independent of Y641-phosphorylation

Our previous study demonstrated that LANA could inhibit IL-4-induced STAT6 signaling pathway and decrease STAT6 phosphorylation [23]. To address whether the nuclear localization and cleavage of STAT6 induced by LANA is independent of phosphorylation on tyrosine 641, we performed immunofluorescence and cell fractionation assays by co-expressing LANA with wild type STAT6 or its mutant Y641F in 293 cells. Strikingly, the results revealed that both wild type STAT6 and its mutant Y641F translocated into the nucleus and was cleaved in the presence of LANA (Fig 3C and 3D), although there was a moderately reduction of STAT6 nuclear localization after Y641 was mutated. To elucidate if nuclear localized STAT6 induced by LANA forms a dimer independent of phosphorylation, we performed dimerization assays using exogenous STAT6 along with IL-4 stimulation as positive control. Our results, revealed that no STAT6 dimers were induced by LANA (Fig 3E, right panel). Similar results were observed using endogenous STAT6 in iSLK with or without KSHV infection (Fig 3E, left panel)

**Fig 4:**
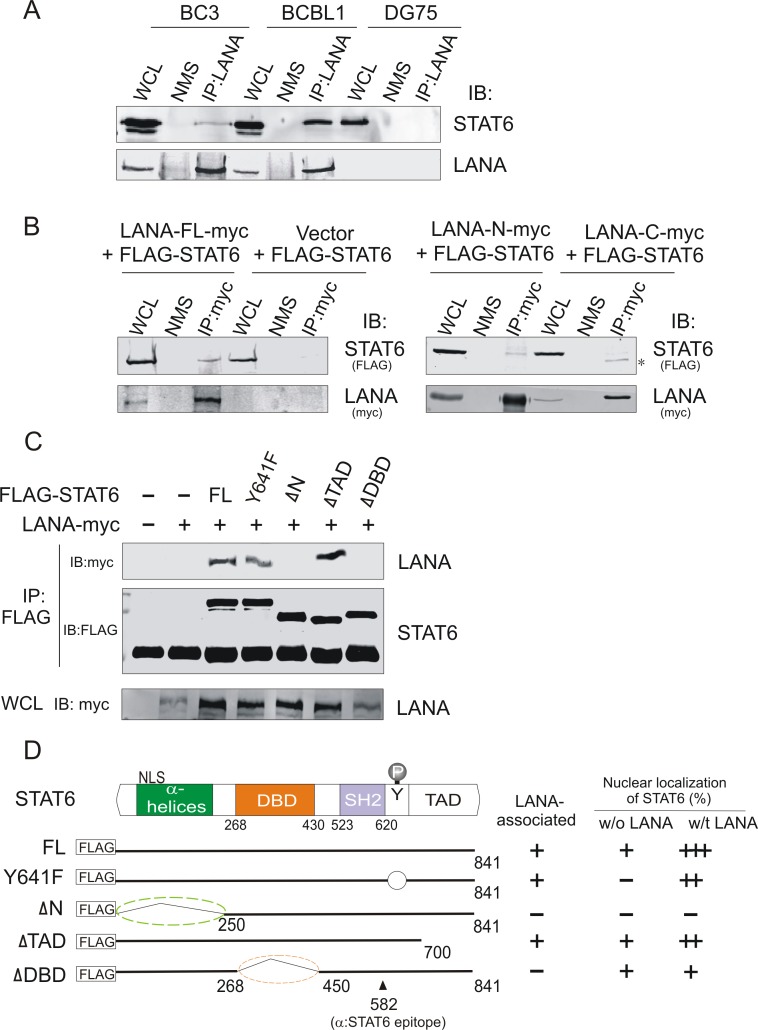
STAT6 is associated with LANA. (**A**) Endogenous STAT6 interacts with LANA in PEL cells. Whole cell lysates (WCL) from PEL cells (BC3, BCBL1) and B lymphoma cells (DG75) were individually subjected to immunoprecipitation (IP) and immunoblotting (IB) assay as indicated. NMS, normal mouse IgG. (**B**) LANA interacts with STAT6 and depends on its amino domain. Whole cell lysate (WCL) of HEK293 cells transfected with STAT6 with FLAG tag in the presence of full-length (FL) LANA or its amino (N) or carboxyl (C) truncated mutants with myc tag, were individually subjected to immunoprecipitation (IP) and immunoblotting (IB) with antibodies against Flag and myc epitope. Asterisk indicates a cleaved isoform of STAT6. (**C**) Exogenous STAT6 interacts with LANA and depends on its amino domain and DNA-binding domain. Whole cell lysate (WCL) of HEK293 cells transfected with STAT6 with FLAG tag in the presence or absence of LANA with myc tag, were individually subjected to immunoprecipitation (IP) and immunoblotting (IB) with antibodies against Flag and myc epitope. (**D**) Schematic of wild type STAT6 and its truncated mutants with FLAG tag. FL, full length; Y641F, tyrosine 641 mutated into phenylalanine 641; DBD, DNA-binding domain; TAD, Transactivation domain; NLS, nuclear localization sequence. The epitope (position 582) recognized by the STAT6 (D3H4) antibody is indicated. The relative ability of STAT6 binding with LANA and percentage of STAT6 nuclear localization is individually summarized from the data of Fig 3C and supplementary S1 Fig and shown on the right panel.

To explore whether the nuclear translocation of STAT6 induced by KSHV is due to the interaction between STAT6 with LANA, we performed an immunoprecipitation assay in KSHV positive (BC3, BCBL1) and negative cell lines (DG75) using a LANA monoclonal antibody, followed by immunoblotting against STAT6. In accordance with our hypothesis, our results demonstrated that STAT6 was pulled down by LANA in KSHV positive cells, but not in KSHV negative cells (Fig 4A). We also demonstrated that exogenous STAT6 did associate with LANA, which is mainly dependent on the amino domain of LANA (Albeit the carboxyl terminus of LANA also presents to selectively associate with the cleaved isoform of STAT6), when they were co-expressed in 293 cells (Fig 4B).

To further address which domain of STAT6 is required for LANA interactions, we generated three truncated mutants of STAT6 based on its functional domains, and performed a reverse immunoprecipitation assay by targeting STAT6. As shown in Fig 4C, deletion of either the α-helics domain (ΔN) or the DNA-binding domain (ΔDBD) significantly abolished the interaction of STAT6 with LANA, when compared with wild type STAT6 or its mutants Y641F and ΔTAD (deletion of transactivation domain). Given the presence of a nuclear localization sequence (NLS) located at the α-helics domain of STAT6, in order to exclude the potential effect of deletion of the NLS on STAT6-LANA interactions, we evaluated the cellular localization of each of the truncated mutants in the presence or absence of LANA in 293 cells by immunofluorescence. We observed (Fig 4D right panel and supplementary S1 Fig) that deletion of the α-helics domain containing NLS abolished STAT6 nuclear localization regardless of whether LANA was co-expressed or not. By contrast, after the deletion of the DNA-binding domain, the nuclear localization of did not significantly change in the presence or absence of LANA when compared with wild type STAT6 or TAD deleted mutants in which the NLS are retained. This indicates that the DNA-binding domain of STAT6 is indeed required for STAT6 to interact with LANA, and that the NLS is necessary for LANA-induced nuclear translocation of STAT6.

### Interaction with STAT6 enhances the stability of LANA

**Fig 5:**
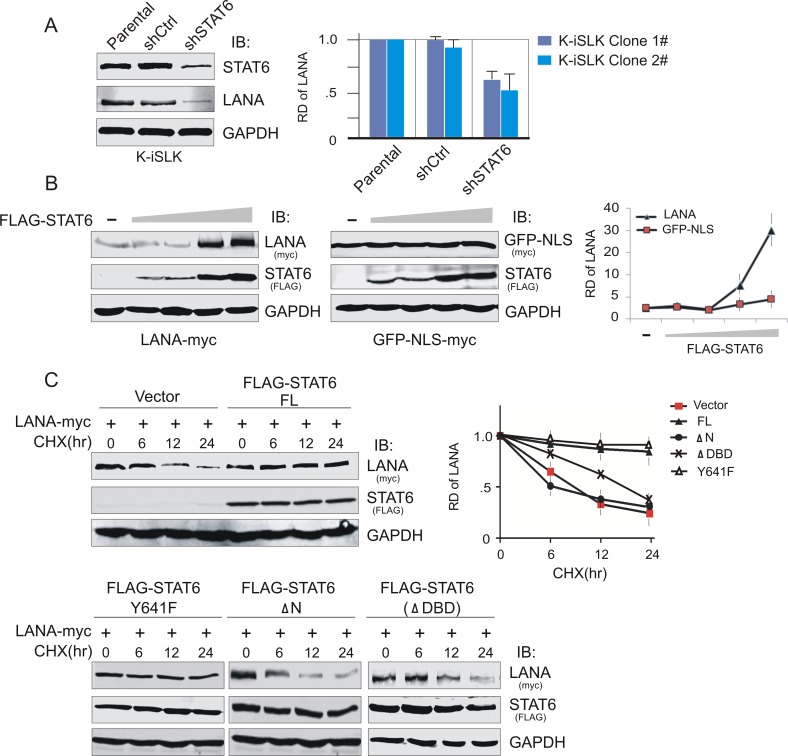
STAT6 contributes to the protein stability of LANA. (**A**) Knockdown of STAT6 reduces the expression level of endogenous LANA in KSHV-infected cells. Parental and K-iSLK cells with lentivirus-mediated knocked down of STAT6 (shSTAT6) or luciferase control (shCtrl), were subjected to immunoblotting. The relative density (RD) of protein level of LANA protein was individually quantitated based on triplicate experiments from two independent clones and shown on the right panel. (**B**) STAT6 stabilizes LANA in a dose-dependent manner. HEK293T cells were co-transfected LANA-myc (2μg) with different dosages of FLAG-STAT6 (0, 1, 2, 5, 10μg). 48hr post-transfection, cells were harvested and lysed for immunoblotting as indicated. The CMV-driven promoter plasmid GFP-NLS-myc was used as a control. The relative density (RD) of protein level of LANA or GFP-NLS is quantified based on triplicate experiments and shown on the right panel. (**C**) STAT6 enhances the protein stability of LANA. HEK293T cells were co-transfected by LANA-myc with full length (FL) FLAG-STAT6, its mutants (Y641F, deletion with amino-domain ΔN or DNA-binding domain ΔDBD) or vector alone. 36hr post-transfection, cells were treated with Cycloheximide (CHX) 20μg/ml for the indicated time before harvesting and lysing for immunoblotting. The relative density (RD) of protein level of LANA is quantified based on triplicate experiments and shown at the right panel.

As knockdown of STAT6 in iSLK cells with KSHV infection reduces the protein expression of LANA (Fig 5A), we hoped to investigate whether the LANA-mediated nuclear translocation of STAT6 also results in an increase in LANA stability. HEK293 cells co-expressing LANA-myc with increasing amounts of FLAG-STAT6 were thus subjected to immunoblotting analysis. In agreement with our hypothesis, co-expression of STAT6 specifically enhanced the level of LANA protein in a dose-dependent manner (Fig 5B, left panel). By contrast, the level of GFP-NLS-myc protein, which is driven by the same CMV promoter, did not significantly change in the presence of increasing amounts of STAT6 expression. Thus, it appears that STAT6 may stabilize LANA.

To further determine the specific manner in which STAT6 contributes to LANA stability, we analyzed the protein stability of LANA co-expression with full-length STAT6, its mutants with nuclear-localization deficient (ΔN), mutation of tyrosine 641 (Y641F), or lacking DNA-binding ability (ΔDBD), or vector alone in 293T cells with cycloheximide treatment for 0, 6, 12 and 24 hours. The immunoblotting results revealed that the stability of LANA protein is significantly enhanced in the presence of full-length STAT6, but not its mutant with nuclear-localization deficiency ΔN, ΔDBD or vector alone (Fig 5C). By contrast, Y641F did not significantly impair the ability of STAT6 to stabilize LANA. Therefore, it would appear that nuclear-localized STAT6 induced by LANA enhances the stability of LANA and is independent of phosphorylation on Y641.

### LANA-induced cleavage of STAT6 in nucleus is sensitive to both serine protease and proteasomal inhibitors

**Fig 6:**
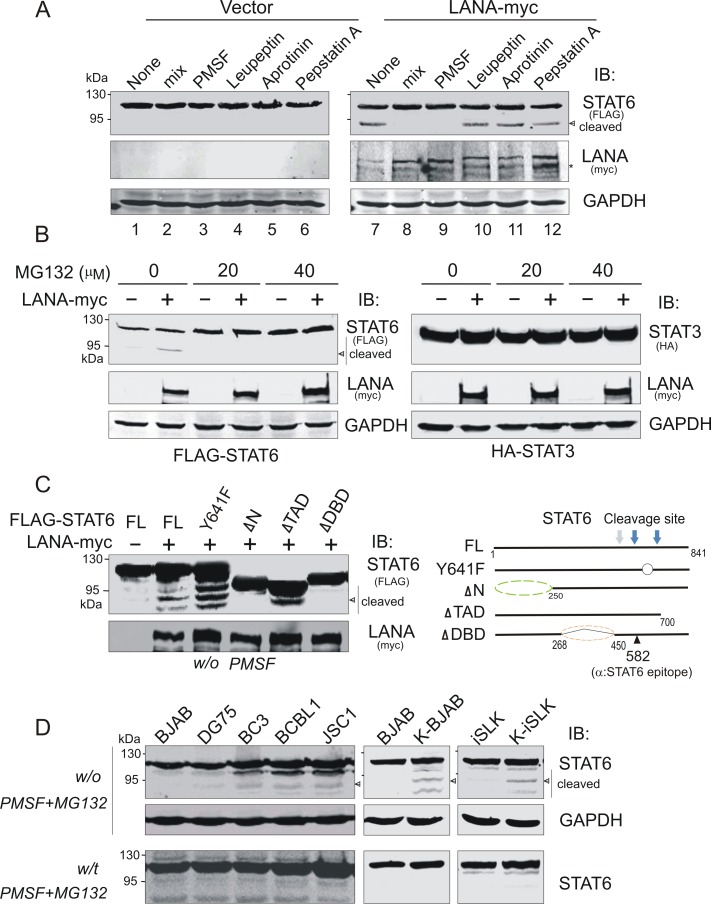
STAT6 is cleaved by serine protease upon KSHV infection. (**A**) LANA promotes serine protease-mediated cleavage of STAT6. HEK293 cells co-transfected by FLAG-STAT6 with LANA-myc or vector alone were lysed in the absence or presence of different protease inhibitors as indicated, followed by immunoblotting against exogenous STAT6, LANA or GAPDH. Asterisk indicates the cleaved bands of LANA. (**B**) LANA-induced STAT6 cleavage is also MG132 sensitive. HEK293 cells were co-transfected by FLAG-STAT6 with LANA-myc or vector alone. At 48hr post-transfection, cells were treated with different dosages of proteasomal inhibitor MG132 for 2hr before harvest, and lysed in the absence of PMSF (serine-protease inhibitor) followed by immunoblotting against exogenous STAT6, LANA or GAPDH. HA-STAT3 was used as a parallel control. (**C**) LANA-induced STAT6 cleavage depends on its interacting domain but not Y641 phosphorylation. HEK293 cells co-transfected by LANA-myc with full length FLAG-STAT6 or its mutants were lysed in the absence of PMSF, followed by immunoblotting against exogenous STAT6 or LANA. The potential cleavage sites of STAT6 induced by LANA are proposed based on the molecular weight of cleaved isoforms and indicated on the right panel. (**D**) Higher levels of STAT6 cleavage in KSHV-positive cells than that in KSHV-negative cells. Cell lysates in the presence or absence of PMSF and MG132 treatment were individually subjected to immunoblotting against STAT6 and GAPDH. Arrow indicates the major band of cleaved STAT6 induced by KSHV or LANA.

To determine which type of protease is critical to contribute to LANA-induced nuclear cleavage of STAT6, HEK293 cells transfected by FLAG-STAT6 with LANA-myc or vector alone, and were subjected to treatment with different protease inhibitors followed by immunoblotting assay. Strikingly, our results revealed that LANA-mediated cleavage of STAT6 was dramatically inhibited by the serine-protease inhibitor PMSF (an inhibitor previously also shown to block nuclear cleavage of STAT6), but not other protease inhibitors (Fig 6A, compare lane 1, 2, 3 with 7, 8, 9). To further delineate the role of LANA in STAT6 cleavage, HEK293 cells were co-transfected by FLAG-STAT6 with LANA-myc or vector alone, were treated with different doses of proteasomal inhibitor MG132 for 2 hours prior to harvest. HA-tagged STAT3 was used as a parallel control. Our immunoblotting results revealed that LANA specifically induces cleavage of STAT6 but not STAT3, and that this LANA-mediated cleavage of STAT6 was also sensitive to proteasomal inhibitor MG132 (Fig 6B).

To further define if LANA-mediated STAT6 cleavage requires specific interactions between STAT6 and LANA, we determined the LANA-induced cleavage profile of STAT6 truncated mutants along with full length and Y641F mutant. Consistent with our hypothesis, both ΔN (which lacks the ability to localize in the nucleus) and ΔDBD (which loses the ability to associate with LANA) STAT6 mutants did not undergo cleavage in the presence of LANA. By contrast, the ΔTAD mutant (which retains the ability to associate with LANA and localizes in nucleus) appeared as cleaved STAT6 band, similar to cleaved bands from full-length STAT6 (Fig 6C, left panel). Unexpectedly, we also observed that the Y641F mutation greatly enhanced the LANA-induced cleavage of STAT6, when compared with wild type STAT6 (Fig 6C, left panel). In addition, based on the observation of three cleaved STAT6 bands induced by LANA, there are at least three potential cleaved sites located at the carboxyl terminus of STAT6 (Fig 6C, right panel).

Importantly, as shown in Fig 6D, in the absence of PMSF and MG132 treatment, we did observe that more cleaved isoforms of STAT6 in the KSHV-positive B lymphoma cells (BC3, BCBL1 and JSC1) and KSHV-infected cells (K-BJAB, and K-iSLK), supporting the conclusion that KSHV induces STAT6 cleavage.

### Cleaved STAT6 induced by LANA blocks RTA transcription via binding to RTA promoter

**Fig 7:**
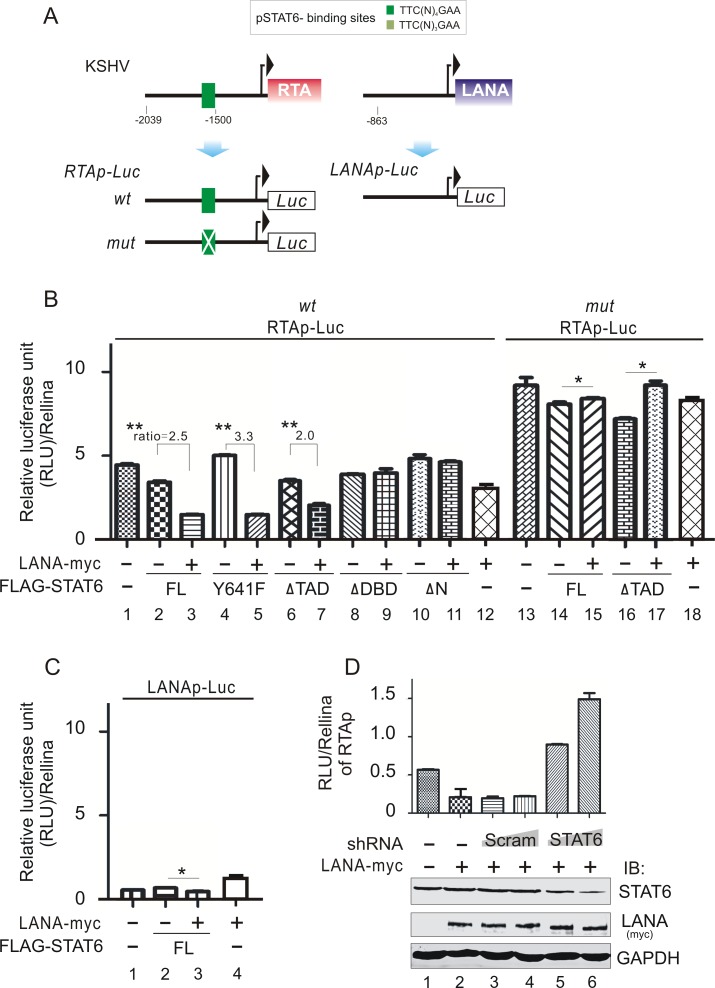
LANA enhances the inhibitory function of STAT6 on transcription of RTA through STAT6-binding sites within RTA promoter. (**A**) Schematic of putative STAT6-binding sites within RTA and LANA promoters. The RTA (or LANA) promoter-driven luciferase reporters RTAp-Luc (or LANAp-Luc) are shown at the bottom panel. LANA enhances the inhibitory function of STAT6 on transcription of RTA promoter (**B**) but not LANA promoter (**C**). HEK293 cells co-transfected with the indicated promoter-reporters combination with full-length (FL) STAT6 or its mutants in the presence or absence of LANA were subjected to reporter assay. Relative luciferase unit (RLU) normalization with Rellina activity was analyzed. Data is presented as meansαSD of three independent experiments. Asterisks indicate *P* value as follows: *, *P*>0.05, and **, *P*<0.05. (**D**) Knockdown of STAT6 abolishes the inhibitory function of LANA on RTA transcription. HeLa cells co-transfected with the indicated plasmids in the presence of RTAp-luc reporter, were subjected to reporter assays as described in panel B.

To elucidate the physiologically functional link between nuclear localization and cleavage of STAT6 induced by LANA during KSHV latent infection, and given that LANA blocks cytokine IL-4-stimulated STAT6 signaling for maintaining latency [23], we speculated that KSHV might also utilize nuclear-localized STAT6 and its cleaved isoform induced by LANA as a negative regulator to repress viral lytic gene transcription. In an attempt to validate our hypothesis, we first analyzed both the promoter sequence of the key latent antigen LANA and lytic activator RTA, and found a canonical pSTAT6-binding site within the RTA promoter but not in LANA promoter (Fig 7A). This provided a clue that the nuclear-localized and cleaved STAT6 may act as a transcription repressor to inhibit RTA expression through binding to its promoter, but without affecting LANA expression. To verify this hypothesis, we generated a luciferase reporter driven by wild type RTA promoter or its mutant with pSTAT6-binding site mutation, and performed reporter assays with full length STAT6 or its truncated mutants in 293T cells. The LANA promoter reported was used as a parallel control. Our results revealed that the RTA promoter was indeed dramatically inhibited by both wild type STAT6 and its cleaved form of STAT6 (ΔTAD) in the presence of LANA, and this inhibition is independent of the status of Y641-phosphorylation and abolished by ΔDBD or ΔN mutant (Fig 7B, compare lane 2, 4, 6, 8, 10 with lane 3, 5, 7, 9, 11). The deletion of the STAT6-binding site within the RTA promoter (mut RTAp-Luc) specifically abrogated the inhibitory activity of LANA-induced nuclear localized and cleaved STAT6 in RTA transcription (Fig 7B, compare lane 1, 3, 7 with lane 13, 15, 17), although the expression of STAT6 in the absence of LANA could block the transcription of RTA to a limited extent (which may due to the NLS localized at the amino terminus of STAT6) (supplementary S2 Fig). By contrast, no significant response of the LANA promoter was observed when it was co-expressed with STAT6 and LANA (Fig 7C, compare lane 2 with lane 3). In addition, knockdown of STAT6 significantly blocks the inhibitory role of LANA in RTA transcription (Fig 7D), suggesting an important role of STAT6 during KSHV latency.

**Fig 8:**
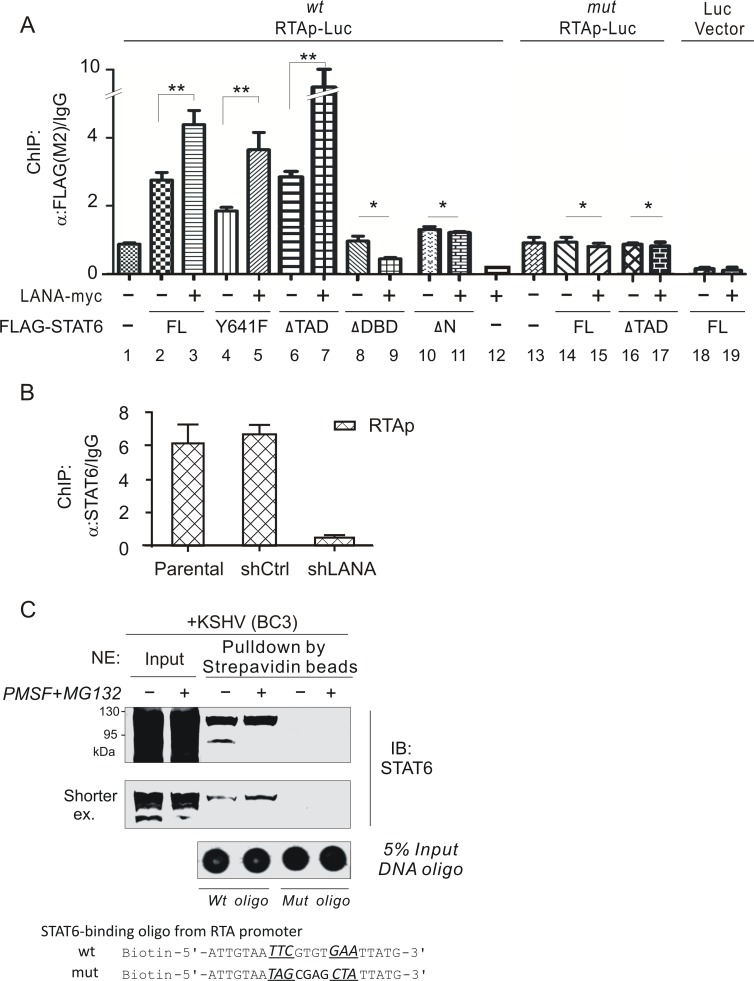
LANA enhances the affinity of STAT6 bound to RTA promoter. (**A**) Intact or cleaved form of STAT6 bound to RTA promoter and enhanced by LANA. HEK293 cells co-transfected with the indicated plasmids, were subjected to Chromatin immunoprecipitation (ChIP) with exogenous STAT6 (full length, Y641F, ΔTAD, ΔDBD, and ΔN). Non-specific mouse IgG and the pGL-3 reporter vector were individually used as control. The relative levels of STAT6 bound to RTA promoter were detected by quantitative PCR. Data is presented as means±SD of three independent experiments. Asterisks indicate *P* value as follows: *, *P*>0.05, and **, *P*<0.05. (**B**) Knockdown of LANA reduces the affinity of STAT6 with RTA promoter. Parental and BC3 cells with lentivirus-mediated constitutively knocked down of LANA (shLANA) or luciferase control (shCtrl) from panel A in Fig 3, were subjected to ChIP assay with endogenous STAT6 as described in panel A. (**C**) In vitro DNA-binding assays of intact STAT6 and its cleaved form from PEL cells. Nuclear extracts (NE) from KSHV-positive BC3 cells with or without PMSF and MG132 treatment, were individually incubated with wild-type or mutant STAT6-binding DNA oligonucleotide derived from RTA promoter followed by three washings, and the precipitates were immunoblotted with STAT6 antibody. Five percent input of DNA oligonucleotide is shown at the bottom.

To verify the inhibitory effect of STAT6 on RTA transcription is due to STAT6 interaction with the STAT6-binding site within the RTA promoter, we performed a chromatin-immunoprecipitation (CHIP) assay using a wild type RTA promoter and a STAT6-binding site mutant in the presence or absence of STAT6 and LANA. Our results demonstrated that LANA dramatically enhanced the affinity of full length STAT6 and its Y641 and ΔTAD mutant bound to the RTA promoter (Fig 8A, compare lane 3, 5, 7 with lane 2, 4, 6), but not its DNA-binding domain (ΔDBD) or NLS-deleted domain (ΔN) mutant (Fig 8A, compare lane 9, 11 with lane 8, 10). By contrast, mutation of the STAT6-binding site significantly reduced the affinity of LANA-mediated full-length STAT6 or its ΔTAD mutant binding to the RTA promoter (Fig 8A, compare lane 15, 17 with 3, 7). Consistent with our observation that LANA induces nuclear localization and cleavage of STAT6, the affinity of STAT6 binding to the RTA promoter was significantly reduced by knockdown of LANA (Fig 8B). These results indicate that induction of both nuclear localization and cleavage of STAT6 is important for LANA to de-regulate functions of STAT6.

To explore whether LANA-induced nuclear localized and cleaved STAT6 in KSHV-latently infected cells interacts with the DNA element of the STAT6-binding site within the RTA promoter, we performed an *in vitro* DNA binding assay by individually incubating the wild-type or the mutated STAT6-binding DNA oligonucleotide, with biotinylated labeling and loading equal amounts of nuclear extracts from KSHV-infected PEL (BC3) cells with or without PMSF and MG132 treatment. The DNA binding activity of both nuclear full length STAT6 and its cleaved form in BC3 cells was significant (Fig 8C, middle panel), whereas little or no signal was seen using the mutant oligonucleotide (Fig 8C, right panel). These results support our hypothesis that LANA-induced nuclear localized STAT6 and its cleaved form is a negative regulator of the RTA promoter by binding to its cognate DNA sequence during latency.

### Ectopically expressed STAT6 inhibits KSHV lytic replication

**Fig 9:**
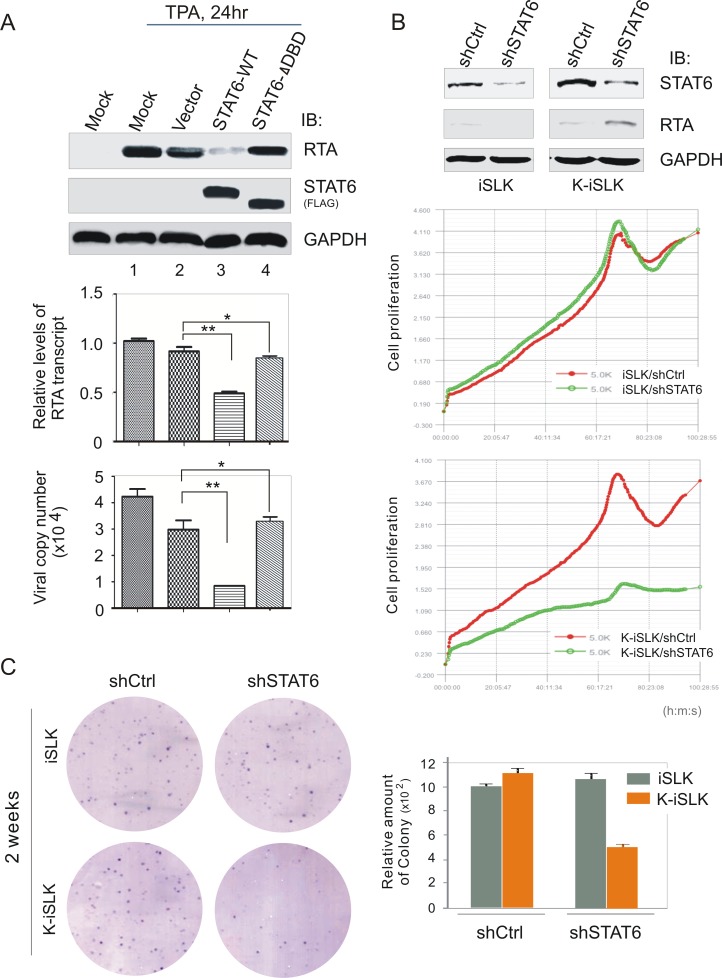
STAT6 is crucial for KSHV to block viral lytic replication and drive cell growth. (**A**) Introduction of intact STAT6 inhibits RTA transcription and virion production. HEK293/Bac36 cells (mock) or HEK293/Bac36 cells transfected with wild-type (WT) or DBD-deleted mutant (ΔDBD) of exogenous STAT6 or vector alone, at 48hr post-transfection, were individually treated with TPA/Sodium butyrate for 24 hr before harvest. Equal amounts of cells were divided for immunoblotting against RTA, STAT6 and GAPDH as indicated in the figure, and RNA extract for quantitative PCR of RTA transcription. The supernatants from culture were purified to quantitate virion production. The statistical significance was evaluated and *p*<0.05 indicated as double asterisks. (**B**) STAT6 knockdown activates RTA expression and reduces KSHV-infected cell growth. iSLK and K-iSLK cells were individually transfected shSTAT6 or shCtrl control. Immunoblotting analysis of endogenous STAT6 and RTA was carried out at 48hr post-transfection. Equal amounts of transfected cells were seeded for real-time monitoring of cell proliferation over a four-day period (bottom panel). (**C**) STAT6 knockdown reduces KSHV-infected cell colony formation. A representative of colony formation after 2-week culture is shown. The relative amount of colony was calculated from two independent experiments (right panel).

To further determine whether introduction of STAT6 alone could block KSHV lytic reactivation, 293-Bac36 cells that harbor an intact KSHV genome were transfected with ectopically expressed wild type STAT6 or its ΔDBD mutant or vector alone, followed by treatment with or without TPA/NaB for 24 hours. Exogenous STAT6 remarkably reduced the transcription and expression of RTA, which blocks viral reactivation and virus progeny production (Fig 9A, compare lane 1, 2 with 3, 4). Consistently, similar results were observed by using K-iSLK cells as target cells (supplementary S3 Fig).

### Disruption of STAT6 expression enhances the expression of RTA and in turn reduces KSHV-induced cell growth

To clarify if disruption of STAT6 expression could turn over the inhibition of RTA expression and in turn impair KSHV latently-infected cell growth, STAT6 in iSLK cells and its derivative KSHV-infected cell line K-iSLK were individually knocked down by lentivirus-mediated small interference RNA against STAT6 (shSTAT6) or luciferase control (shCtrl), followed by immunoblotting assays and real-time monitoring of cell growth rate. The results revealed that knockdown of STAT6 in KSHV-infected cell line K-iSLK significantly enhances RTA expression (Fig 9B, top panel), and in turn reduces cell growth when compared with luciferase control (Fig 9B, low panel). By contrast, no significant difference was observed in the K-iSLK parent cell iSLK (Fig 9B, middle panel). In addition, the results of a two-week culture period demonstrated that STAT6 knockdown alone also markedly blocked colony formation of K-iSLK cells but not iSLK cells, when compared with luciferase knockdown control (Fig 9C). Similar results were observed in BC3 cells with STAT6 knockdown (supplementary S4 Fig). Thus, it appears that LANA-mediated nuclear localization and cleavage of STAT6 plays an important role for KSHV to drive viral latency in host cells.

## Discussion

**Fig 10:**
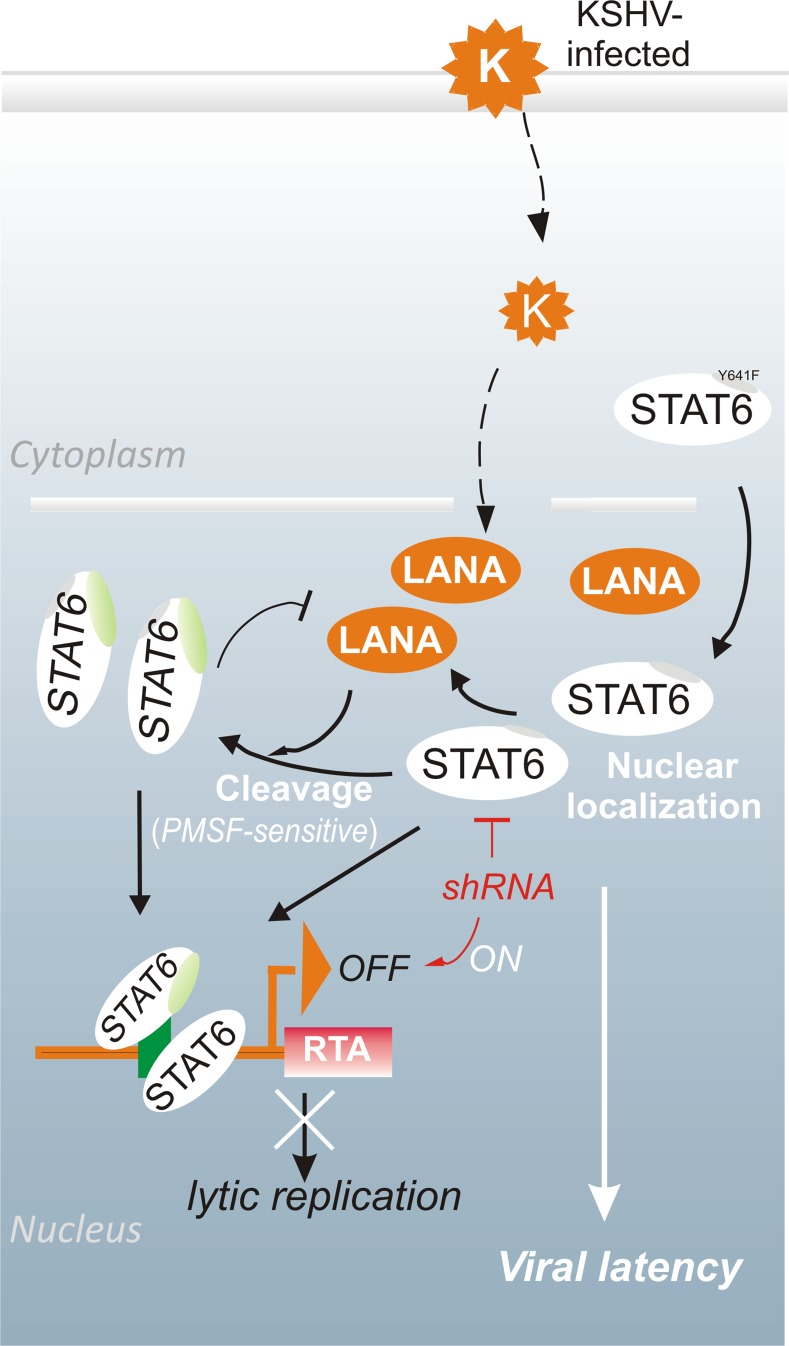
A schematic representation of cytokine-independent regulation of STAT6 upon KSHV infection. In the KSHV latently-infected cells, KSHV encoding LANA induces nuclear translocation of STAT6 independent of Y641-phosphorylation stimulated by cytokine IL-4 or IL1-3. The interaction between STAT6 and LANA not only stabilizes LANA but also leads to serine protease (PMSF sensitive)-mediated cleavage of STAT6 in the nucleus, which in turn represses RTA transcription and lytic replication, and facilitates viral latency and pathogenesis.

In the present study, we demonstrated a novel regulatory mechanism of KSHV-mediated STAT6 signaling in KSHV-infected cells. We found that STAT6 was induced by KSHV to translocate into the nucleus independent of tyrosine 641-phosphorylation. The nuclear localization of STAT6 was due to the interaction with the latent antigen LANA, and in turn led to STAT6 cleavage in the nucleus of KSHV-infected cells. The nuclear-localized and cleaved STAT6, induced by LANA, inhibited the transcription of RTA, and blocked lytic replication and viral progeny production. Conversely, full length nuclear-localized STAT6 enhanced the protein stability of LANA for viral latency (Fig 10). Consistent with our previous studies [23,24], these findings explain why LANA could block the IL-4-induced phosphorylation of STAT6, and why IL-13-mediated constitutively phosphorylation of STAT6 was dramatically enhanced at the early stage (< 3 days), but reduced later (>5 days) along with the increased expression of LANA during KSHV primary infection (which could be due to the effect of nuclear localization of STAT6 induced by LANA).

Our finding that LANA-mediated STAT6 cleavage in the nucleus was inhibited by PMSF, but resistant to a number of other protease inhibitors including pepstatin, leupeptin and aprotinin, suggests that STAT6 cleavage is caused by a serine protease. In addition, proteasome inhibitor MG132 could also indirectly block LANA-mediated STAT6 cleavage, further supporting this conclusion. Moreover, Y641F STAT6, which is defect for IL-4-induced phosphorylation and less nuclear localization, translocates into the nucleus in the presence of LANA and is more sensitive to LANA-mediated cleavage. Although other proteases may be present in different cells lines we observed STAT6 cleavage in B cells, endothelial cells and epithelial cells, indicating that STAT6 cleavage is tightly regulated by KSHV infection. Taken together, our results revealed a previously uncharacterized pathway for KSHV pathogenesis which includes nuclear translocation and cleavage of STAT6.

A recent report suggested that STING is required for Sendai virus-induced phosphorylation of STAT6 on Y641, and leads to STAT6 translocation in nucleus after virus infection [25]. Similar to the interaction of STAT6 with STING, the DNA-binding domain of STAT6 is required for interactions with LANA. However, further investigation is required to determine if LANA competes or cooperates with STING. Unlike other STATs, no detailed mechanisms for driving the nuclear import or export of STAT6 have been identified yet. We could not exclude the possibility that cleaved STAT6 induced by LANA is not generated in the nucleus but is present in the cytoplasmic fraction due to protein shuttling. For example, recent reports have shown that STAT6 phosphorylation on Tyr^641^ was induced by viral or parasitic infection independent of IL-4 [25–27]. However, the canonical STAT6 signaling induced by IL-4 is independent of STING or MAVS, which help viruses trigger STAT6 phosphorylation [28]. This could also address why KSHV selectively responds to IL-4 and IL-13 stimulation as observed in our previous studies [23,24].

There still remains some controversy with regard to how KSHV regulates unstimulated STAT6 and constitutively phosphorylated STAT6 by IL-13 during latency. The discoveries by our and other have shown that dephosphorylation of STAT6 is restricted to the nucleus [29], and in addition to tyrosine phosphorylation, serine phosphorylation is also necessary for STAT6 to exert full transcriptional potency [30], could be an explanation for this discrepancy. On the other hand, although the consequences of serine phosphorylation of STAT6 remain obscure, IL-4 has been shown to induce the phosphorylation of STAT6 not only on tyrosine 641 but also serine on 756 [31]. In contrast, in addition to stimulation by cytokine IL-4 or IL-13, some studies present alternatives to the canonical induction of STAT6 by IL-15 in mast cells [32], by PDGF in fibroblast cells [33], or by IFNα in B cells [34]. Other posttranslational modifications such as acetylation and methylation could explain the complicated regulation of STAT6-mediated activation of expression [35,36].

It has been demonstrated that there exists four isoforms of STAT6 in humans, which potentially contribute to competition of activated STAT6 signaling. However, in different cell types, the cleavage of STAT6 by proteases is different. It has been suggested that the elastase family is responsible for cleavage of STAT6 in mast cells [8], whereas the calpains family has been reported to be responsible in T cells. However, unlike the production of a 65kD cleaved STAT6 in mast cells, degradation in T cells seems to be complete [37]. Our findings revealed that STAT6 cleavage by serine proteases could be induced by KSHV infection in B cells, endothelial and epithelial cells. Consistent with previous studies [7], our results also suggest that STAT6 cleavage is not observed in healthy B cells but appears in KSHV-infected B cells, indicating that KSHV induces STAT6 cleavage.

In agreement with a previous report [38], we observed that the cleavage positions of LANA-induced STAT6 with carboxyl terminal-truncated mutants are mainly located between 673 and 695 amino acids, and the STAT6 protease activity is present in the nucleus and inhibited by a serine protease inhibitor PMSF. Although the carboxyl truncated cleavage of STAT3 is also similarly regulated by proteolytic processing during terminal differentiation of neutrophils [39], in contrast to STAT6, we did not see any apparent cleavage of STAT3. This suggests that the cleavage of STAT6 is specifically induced by KSHV.

Interestingly, recent studies reported that LANA also undergoes caspase cleavage in response to oxidative stress [40]. In this study, we observed that STAT6 also pulled down the cleaved isoform of LANA at the amino terminus, and several protease inhibitors including PMSF greatly enhanced the level of LANA expression. However, further studies will be required to determine if the protein stability of LANA enhanced by its interaction of STAT6 is through blocking the cleavage of LANA.

In accordance with a previous observation that STAT6^-/-^ mice present higher virus titer than wild type control [25], our studies found that inhibition of STAT6 by small hairpin RNA interference in KSHV-latently infected cells also reactivates lytic replication by enhancing RTA expression, which in turn leads to reduction of cell growth and colony formation of KSHV-infected cells. We also demonstrated that ectopic expression of STAT6 in HEK293 cells carrying KSHV genome is sufficient to block TPA/Sodium butyrate-induced RTA transcription and viral production, respectively. Strikingly, constitutive expression of C-terminally truncated STAT5 proteins in CD4 T cells from HIV patients on treatment have been shown to be associated with good response to therapy [41]. However, it is not known whether the truncated cleavage of STAT6 in KSHV-latently infected cells may influence the outcome of disease progression, and need to be further investigated.

## Materials and Methods

### Ethics statement

De-identified 3μm paraffin-embedded KS patient tissues were obtained from public health clinical center of Fudan University. Usage of redundant cancer sample for research purpose was approved by the Hospital Medical Ethics Committee. The IRB approved protocol in which Declaration of Helsinki protocols were followed and each donor gave written, informed consent.

### DNA constructions and antibodies

Plasmids expressing STAT6 truncation mutants ΔN and ΔDBD was constructed by PCR amplicon (FLAG-STAT6 as template, a gift from Jaharul S. Haque at Lerner Research Institute) inserted into pcDNA3.1-FLAG-C1 with restriction enzymes *EcoR*I and *Xho*I, respectively. STAT6 mutants ΔTAD and Y641F were individually constructed by PCR-directed site mutation with 701 stop codon and Y641F. Luciferase reporter RTAp-luc with wild type RTA promoter was described previously [42], RTAp-luc with STAT6-binding site mutation (TTCCGCGGAA into TATATGTCTA) was obtained by PCR-directed site mutation with RTAp-luc as template. Plasmids LANA-myc (WT, N: 1–340), GFP-LANA-C(930–1162)-myc, RFP-LANA, LANAp-Luc, and pCDNA3.1-GFP-NLS-myc was described previously [42,43].

Antibodies to STAT6 (D3H4, Cell signaling for WB; YE361, Abcam for IHC and IF), α-tubulin (1C6, Santa cruz), Histone H3 (#8226, Abcam), FLAG (M2, Sigma), and GAPDH (G8140-01, US Biological) were used according to the manufacturers specifications. The monoclonal antibody anti-myc (9E10) and HA (12CA5) were prepared from hybridoma cultures. Mouse monoclonal antibodies against LANA and RTA were kindly provided by Ke Lan from Shanghai Pasteur Institute of CAS. PMSF, Leupeptin, Aprotinin, and Pepstatin A were purchased from Amresco. TPA was purchased from Sigma and sodium butyrate from J&K Corporation. Proteasome inhibitor MG132 was purchased from Biomol International, and Cyclohexamide (C4859, Sigma Inc., St. Louis, MO). Chelex 100 Resin (#142–1253) was from Bio-Rad.

### Cell culture and transfection

KSHV-negative (BJAB and DG75 from American Type Culture Collection [ATCC], Manassas, VA) and positive (BC3, BCBL1, and JSC1 from ATCC) B-lymphoma cells, iSLK (1mg/ml hygromycin, 250ug/ml G418, a gift from Shou-Jiang Gao at University of South California) and iSLK-Bac16 (K-iSLK, 1mg/ml hygromycin, 250ug/ml G418 and 1ug/ml puromycin, a gift from Shou-Jiang Gao at University of South California) cells were maintained in RPMI 1640 medium supplemented with 10% fetal bovine serum (FBS) and 1% penicillin and streptomycin (Gibco-BRL). HUVEC (ATCC), HeLa (ATCC), HEK293 (ATCC), and 293/BAC36 (a gift from Erle Robertson at University of Pennsylvania) which harboring wild- type KSHV (1ug/ml puromycin) cells, were maintained in DMEM supplemented with 10% FBS. All cell lines were incubated at 37°C in a humidified environmental incubator with 5% CO_2_. 293 and 293/Bac36 cells were transfected with 1 mg/ml polyethyleneimine (PEI) at a ratio of 1μg plasmid DNA: 3μl PEI. iSLK and iSLK-Bac16 were transfected with Lipofectamine 2000 reagent (Invitrogen) according to the manufacturer’s recommendations. Cells were transfected at culture for 24hrs with cell confluence reaching 60–70%. B-cells transfection was performed with Lonza-4D nucleofector system in an optimized program CA137.

### Immunofluorescence assay

Immunofluorescence assays were performed as described previously [44]. Briefly, cells were harvested and plated in polylysine-treated coverslips for 4hrs in CO_2_ incubator to let cells attach to the plate. And then washed with ice-cold PBS twice, and fixed in 4% paraformaldehyde for 20 min at room temperature. After fixation, cells were washed three times in PBS and permeabilized in PBS containing 0.2% fish skin gelatin (G-7765; Sigma) and 0.2% Triton X-100 for 5 min, followed by primary and secondary antibody staining. Nucleus was counterstained with 4, 6-diamidino-2-phenylindole (DAPI), and coverslips were mounted with *p*-phenylenediamine. Cells were visualized with Leica SP8 confocal microscope.

### Immunoprecipitation and immunoblotting

Cells were harvested, washed once with ice-cold PBS, and lysed in 600μl ice-cold RIPA buffer [150 mM NaCl, 50 mM Tris (pH7.6), 1% Nonidet P-40, 2 mM EDTA, 1 mM phenylmethylsulfonylfluoride (PMSF), 1 mM Na_3_VO_3_, 1 g/ml aprotinin, 1 g/ml leupeptin, 1 g/ml pepstatin] for 30min with constant shaking at 4°C. Cell debris was removed by centrifugation at 14,500 rpm at 4°C for 10min. The supernatants were then transferred to a new eppendorf tube. Five percent of the supernatant was used as input. The rest lysates were then precleared with normal mouse IgG (Invitrogen) and protein A/G Sepharose beads by end-over-end rotation at 4°C for 1hr. After preclear, beads were spun out, washed with ice-cold RIPA buffer for four times and re-suspended with 30μl RIPA buffer. Supernatant was then incubated with primary antibody at 4°C with rotation overnight. Protein of interest complexes were captured the next day with 30μl protein A/G Sepharose beads with rotation on 4°C for another 4hrs. Beads were spun out, washed with ice-cold RIPA buffer for four times and re-suspended with 30μl RIPA buffer. For immunoblotting assays, after input lysates, immunoprecipitated (IP) complexes were boiled in 6xSDS loading buffer, proteins were fractionated by SDS-PAGE, and transferred to a 0.45-mm nitrocellulose membrane. The protein of interest in the membrane was probed with primary antibodies at 4°C with shake for overnight, followed by incubation with appropriate secondary antibodies for another 1hr at room temperature. The member was scanned with an Odyssey Infrared scanner (Li-Cor Biosciences). Densitometric analysis was performed with the Odyssey scanning software.

### Purification and quantitation of KSHV virion

HEK293-Bac36 (KSHV) cells were individually induced with 20 ng/ml of tetradecanoyl phorbol acetate (TPA) and 1.5 mM sodium butyrate (NaB) for 2 days at 37°C with 5% CO_2_. After induction, the supernatant of culture medium was collected and filtered through a 0.45μm filter, and viral particles were spun down at 25,000 rpm for 2h at 4°C. The concentrated virus was collected and used for virion quantitation by qPCR.

### Cell proliferation and colony formation assay

iSLK and iSLK-Bac16 cells was individually transfected with RFP-tagged plasmid GV113 containing shControl or shSTAT6 for 48hrs. Equal amount cells were then seeded in 16 well with normalization of transfection efficiency by flow cytometer assay. Cells growth rate was real-time measured by xCELLIgence system (ACEA Biosciences, Inc.). Experiments were performed in triplicate with 6 repeats each time point. For colony formation, iSLK and iSLK-Bac16 cells were trysinized and equal amount cells were dispersed to 10cm plate. After 14 days, cell culture supernatants were discarded and cells were fixed with 4% (v/v) formaldehyde and stained with 0.1% crystal violet. Colony formation in each dish were scanned by Li-Cor Odyseesy and counted. Experiments were performed in triplicate.

### Dimerization assay

STAT6 dimerization experiment was performed with freshly made solution of disuccinimidyl suberate (DSS, Thermo Scientific). At 36hr-posttransfection, cells were harvested and washed three times with ice-cold PBS (pH 8.0) to remove amine-containing culture media and proteins from the cells. Cells pellet was re-suspended in PBS to reach a concentration of 2.5x10^7^ cells per ml, and cross-linked with 1mM DSS for 30 minutes at room temperature. The cross-link reaction was stopped by addition of 20mM Tris-HCl (pH 7.5) and incubated for another 15 min at room temperature, followed by washing with PBS for once and lysis with RIPA for 30min at ice. The cell lysates were separated by SDS-PAGE and analyzed by immunoblotting with STAT6 antibody (YE361, Abcam) for endogenous dimer of STAT6.

### Fractionation of nuclear or cytoplasm proteins

Cells were harvested and washed twice with ice-cold PBS followed by resuspending and lysis cell pellet in a hypotonic buffer A (10 mM HEPES-K^+^ pH 7.5, 10 mM KCl, 1.5 mM MgCl_2_, 0.5 DTT) containing 0.1% NP-40 for 5 min on ice. The cytoplasm fraction was obtained by spinning at 3000 rpm ×5 min at 4°C. The supernatant (cytoplasm protein) harvested and frozen at −80°C for use. The nuclear pellets were washed twice with buffer A (without NP-40), resuspended in RIPA buffer and sonication with 30% of the maximum power output (10s on/off) for two rounds, followed by 14500 rpm for 5 min at 4°C, supernatant (nuclear protein) was harvested and snap frozen for further use. The efficiency of cytoplasm and nuclear extraction were verified by immunoblotting with Histone H3 and α-Tubulin antibodies, respectively.

### Chromatin immunoprecipitation

Chromatin immunoprecipitation (ChIP) assay were performed with some modification as described previously [45–48]. At 48hr post-transfection, approximate thirty millions of cells were cross-linked with 1.42% (vol/vol) formaldehyde for 5min at room temperature for 10 min. Formaldehyde was quenched by adding 125mM glycine and incubate at room temperature for 5min. Fixed cells were scraped and washed with ice-cold PBS twice and lysed with 1ml IP buffer (150mM NaCl, 50mM Tris-Hcl [pH7.5], 5mM EDTA, 0.5%NP40(v/v), 1.0% TritonX-100 (v/v) with protease inhibitors [1 mM PMSF, 10mM Na_3_VO_3_, 2μg/ml aprotinin, 5μg/ml leupeptin, 1μg/ml pepstatin]) and incubated for 15 min with constant shaking at 4°C. Nuclear component was obtained by centrifuge at 12,000g for 1min at 4°C and nuclear pellet was washed with IP buffer and re-suspended in 600 μl IP buffer. Chromatin was sheared with sonication to an average fragment size of 300 to 500 base pairs. Solubilized chromatin extracts were cleared by centrifugation at 12,000g for 10min at 4°C. 20μl supernatant was transferred to a new eppendorf tube to determine shearing efficiency and extract DNA as input. Rest of the sample was used for immunoprecipitation. Antibody against interest protein was added to the sheared chromatin and incubation overnight at 4°C with shaking. For mock, non-specific IgG was used as a control. The chromatin complex was precleared the next day with centrifuge at 12000g for 10min at 4°C. 90% supernatant was transferred to a new tube and chromatin complex was captured by adding protein A/G Sepharose beads with rotation at 4°C for 1hr. After incubation beads were washed five times with 1ml IP buffer to exclude non-specific binding. To elute DNA, 100ul chelex 100 (10% w/v) (Bio-Rad) was added to the washed beads and boiled for 10 min, followed by treatment with 1μl proteinase K (20μg/μl) and incubation at 56°C for 30min, and boiled for another 10min to inactivate proteinase K. condensate DNA was centrifuged at 12,000g for 1min at 4°C and washed with 120μl ddH_2_0, supernatants of the two elution were collected. Input DNA was precipitate with 2 volumes of ethanol, and washed with 70% (v/v) ethanol. The pellet was re-suspended in 100ul chelex 100 (10% w/v) and boiled for 10min and continue processing as IP sample. Purified DNA was amplified by quantitative PCR using RTA specific primers (forward: 5'-CCCGACTAATGAGGACAAT-3', and reveres: 5'-TTCAAACCCATCATCTGTG-3').

### Immunohistochemistry

Immunohistochemistry of STAT6 was performed on deparaffinized, formalin-fixed tissue sections using an indirect immunoperoxidase method with an automated immunostainer as described previously [24].

### RNA interference

iSLK and iSLK-Bac16 cells with STAT6 knockdown were individually performed by transient transfection with STAT6 shRNA. STAT6 shRNA sequence (GGGAGAAGATGTGTGAAACTCTGAA) was inserted into lentivirus pGV113 vector which carrying a RFP protein. At 48hr post-transfection with PEI method, cells were visualized under florescence microscope and flow cytometer assay to check transfection efficiency and RNA interfering efficiency was assessed by immunoblotting against STAT6. pGV113 vector with luciferase (shLuc) target (TGCGTTGCTAGTACCAAC) sequence was used as control.

### Dual-luciferase reporter assay

Transfected cells at 48hr post-transfection were lysed with 200ul Passive lysis buffer (Promega), followed by dual luciferase reporter assay according to the manufacturer’s instruction as described previously. *Renilla* luciferase was used as a control to normalize the transfection efficiency. Relative luciferase activity [RLU] was expressed as fold changes relative to the reporter construct alone. Assays were performed in triplicate.

### RNA extraction, reverse transcription, and quantitative PCR

Total RNA from cells was extracted using TRIzol regent (invitrogen) according to the manufacturer’s Instructions. 1μg RNA was reverse transcripted with a Superscript II reverse transcription kit (Invitrogen, Inc., Carlsbad, CA). After reverse transcription, 1μl cDNA was used as template for quantitative PCR. The RTA primers (5’-CAGACGGTGTCAGTCAAGGC-3’ and 5’-ACATGACGTCAGGAAAGAGC-3’) and GAPDH (5’-ACGACCACTTTGTCAAGCTC-3’ and 5’-GGTCTACATGGCAACTGTGA-3’) was used as an internal control. The cDNA was amplified in a total volume of 20ul containing 10 μl of SYBR premix Ex Taq (Takara, Inc.), 0.5 μl each primer (10μM), 1μl cDNA and rest of RNAase free water. PCR program was running on thermocycler (Bio-Rad Inc.) in a procedure of 40 cycles of 1 min at 94°C, 30 s at 55°C, and 30 s at 72°C, followed by 10 min at 72°C. A melting-curve analysis was performed to verify the specificities of the amplified products. Each sample was tested in triplicate and date was summarized from three independent experiments. The relative mRNA fold changes relative to GAPDH were calculated by the threshold cycle (*CT*) method.

### Statistical analysis

Statistical significance of differences between means of at least n = 3 experiments was determined using Student’s *t*-test.

## Supporting Information

S1 Fig HEK293 cells transfected with truncated mutants (ΔN, ΔTAD, ΔDBD) of STAT6 with FLAG tag (green) in the presence or absence of LANA with RFP tag (red) imaged by confocal assay. Nuclei were stained with DAPI. The relative percentage of STAT6 nuclear localization (bottom panel) was individually quantified by nuclear and cytoplasmic staining of 100 cells.
(TIF)

S2 Fig STAT6 inhibits RTA promoter but not its mutant with STAT6-binding site mutation or LANA promoter in a dose-dependent manner. HEK293 cells co-transfected with the indicated promoter-reporters at different dosages (0, 2, 5μg) of FLAG-STAT6 were subjected to reporter assay. Relative luciferase unit (RLU) normalization with Rellina activity was analyzed. Data are means ±SEM. Immunoblotting analyses of exogenous STAT6 is shown in the bottom panel. GAPDH was used as control.
(TIF)

S3 Fig Introduction of intact STAT6 inhibits RTA transcription and virion production. K-iSLK cells (mock) or K-iSLK cells transfected with wild-type (WT) or DBD-deleted mutant (ΔDBD) of exogenous STAT6 or vector alone, at 48hr post-transfection, were individually treated with TPA/Sodium butyrate for 24 hr before harvest. Equal amounts of cells were used to RNA extract for quantitative PCR of RTA transcription. The supernatants from culture were purified to quantitate virion production. The statistical significance was evaluated and *p*<0.05 indicated as double asterisks.
(TIF)

S4 Fig STAT6 knockdown reduces KSHV-infected BC3 cell growth. PEL BC3 cells were individually transfected shSTAT6 or shCtrl control. Equal amounts (2.5 million) of transfected cells were seeded and monitored the cell growth for 3 days.
(TIF)
