# Expression of Concern: Nuclear Localization and Cleavage of STAT6 Is Induced by Kaposi’s Sarcoma-Associated Herpesvirus for Viral Latency

**DOI:** 10.1371/journal.ppat.1010047

**Published:** 2021-12-15

**Authors:** The PLOS Pathogens Editors

Following the publication of this article [1], concerns were raised regarding results presented in Figs 3, 5, and 9, and S1 Fig. Specifically,

- Irregularities and discontinuities have been detected in the background of the following panels:
  - Fig 3B: STAT6 panel, Tubulin panel, and Histone H3 panel.
  - Fig 6B: STAT6 (FLAG) panel and corresponding GAPDH panel.
- In Fig 3C, the WT LANA—LANA panel appears to be devoid of any background noise.
- In Fig 5C, the following panels appear similar:
  - FLAG-STAT6 Y641F LANA (myc) and FLAG-STAT6 ΔN STAT6 (FLAG) if stretched vertically.
  - FLAG-STAT6 Y641F GAPDH and FLAG-STAT6 ΔN GAPDH.
- In Fig 9C, the iSLK shCtrl panel partially overlaps with the iSLK shSTAT6 panel when rotated.
- In S1 Fig, the LANA—LANA panels for ΔN, ΔTAD, and ΔDBD appear to be identical.

The corresponding author indicates that the FLAG-STAT6 Y641F panels of Fig 5C and the iSLK-shSTAT6 panel of Fig 9C are incorrect and provided the updated Figs 3, 5 and 9, and raw image data underlying the blots and images presented in this article in the Supporting Information S1–S7 Files below. Some of the underlying data provided in the S7 File are of too low resolution to confirm the results presented in the published figures, or did not appear to be an exact match with the published results. Furthermore, the underlying blots provided raised additional concerns with the Fig 5C FLAG-STAT6 ΔN LANA (myc) and the Fig 6A Vector STAT6 (FLAG) panels as they did not appear to present an exact match with the underlying data, as well as the Fig 3C LANA Y641F LANA (-) panel where the underlying data did not match the colour of the signal in the published fig.

A member of the *PLOS Pathogens* editorial board has reassessed the article and the underlying data provided by the authors and indicated that due to the unresolved concerns with results presented in this article, the conclusions about LANA promotion of nuclear localisation of STAT6 independent of its tyrosine-641 phosphorylation state and LANA induced serine protease cleavage of STAT6 are questionable.

The *PLOS Pathogens* Editors issue this expression of concern to notify readers of the above issues and to provide the available underlying data.

**Fig 3:**
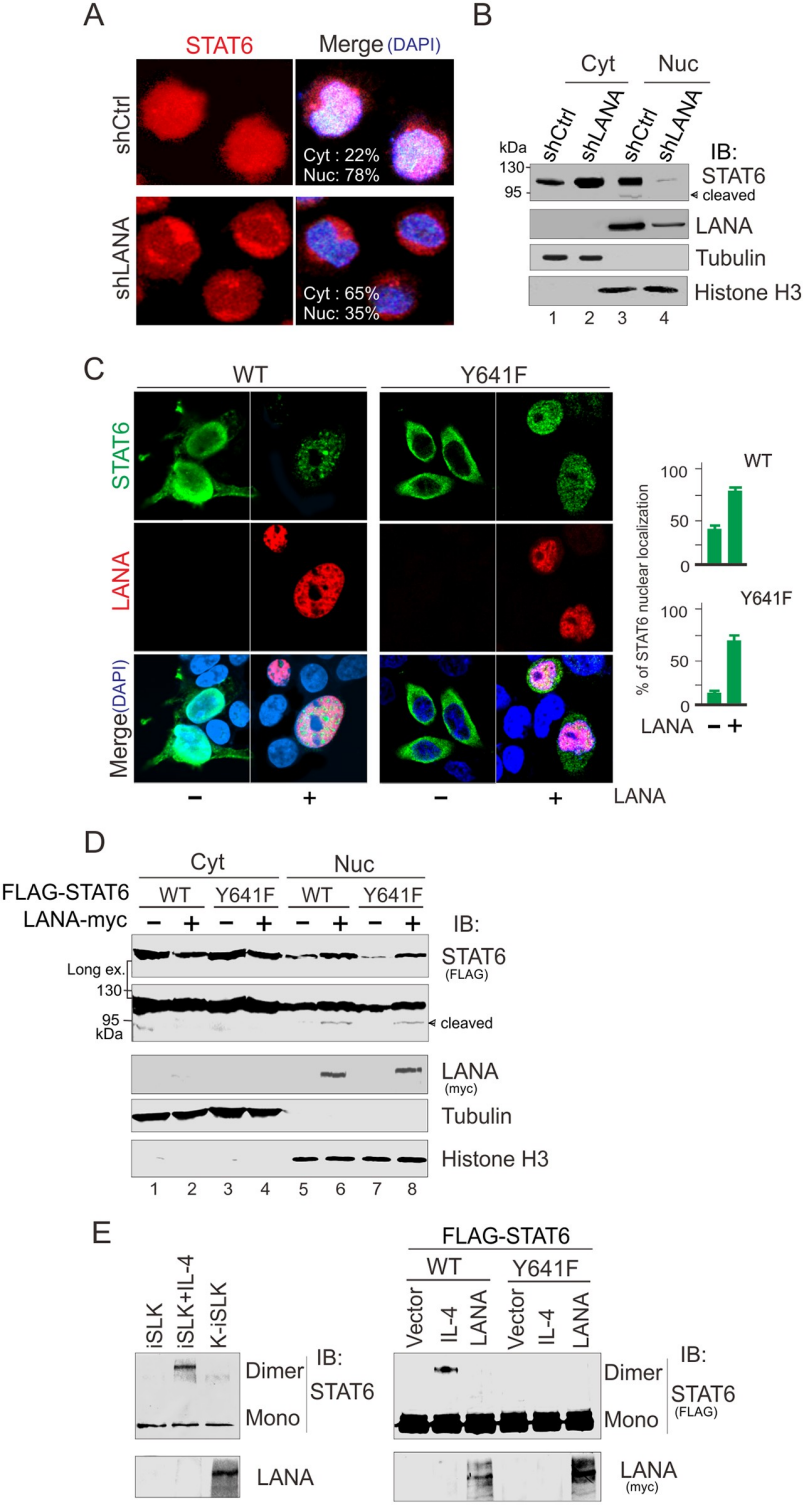
LANA promotes nuclear localization of STAT6 independent of Y641-phosphorylation. (**A**) Inhibition of LANA blocks nuclear localization of STAT6. Confocal microscopy of endogenous STAT6 (red) and merged in PEL cells BC3 with lentivirus-mediated constitutively knocked down of LANA (shLANA) or luciferase control (shCtrl). Nuclei were stained with DAPI. (**B**) Western blot analyses of fractionated BC3 cells with LANA or luciferase control knockdown. Cyt, cytosolic; Nuc, nuclear; were revealed by α-tubulin and Histone H3, respectively. (**C**) LANA induces nuclear localization of exogenous STAT6 independent of phosphorylation on tyrosine 641. HEK293 cells transfected with wild type (WT) or Y641 mutant (Y641F) of STAT6 with FLAG tag (green) in the presence or absence of LANA with RFP tag (red) were imaged by confocal assay. Nuclei were stained with DAPI. The relative percentage of STAT6 nuclear localization (right panel) was individually quantitated by nuclear and cytoplasmic staining of 100 cells. (**D**) Immunoblotting analyses of fractionated 293 cells transfected with expressing plasmids as indicated. Cyt, cytosolic; Nuc, nuclear; were revealed by α-tubulin and Histone H3, respectively. (E) Cells from panel C in Fig 1 or panel D were individually treated with DSS for 30 minutes before lysis and analyzed by immunoblotting (IB) for dimerization of STAT6 (data is shown on the left and right panels, respectively). Cells treated with IL-4 (50 ng/ml) for 30 min were used as control.

**Fig 5:**
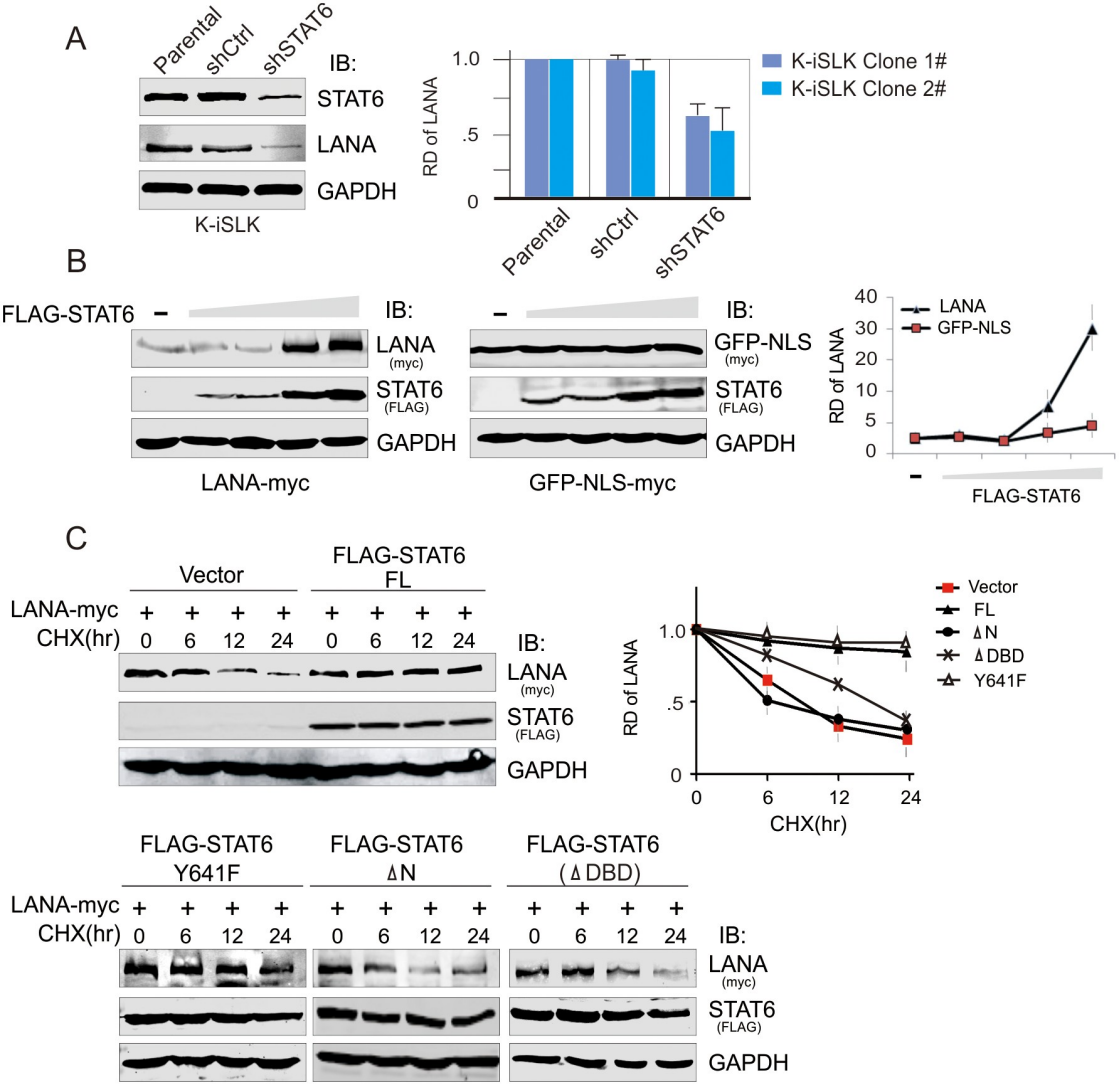
STAT6 contributes to the protein stability of LANA. (**A**) Knockdown of STAT6 reduces the expression level of endogenous LANA in KSHV-infected cells. Parental and K-iSLK cells with lentivirus-mediated knocked down of STAT6 (shSTAT6) or luciferase control (shCtrl), were subjected to immunoblotting. The relative density (RD) of protein level of LANA protein was individually quantitated based on triplicate experiments from two independent clones and shown on the right panel. (**B**) STAT6 stabilizes LANA in a dose-dependent manner. HEK293T cells were co-transfected LANA-myc (2μg) with different dosages of FLAG-STAT6 (0, 1, 2, 5, 10μg). 48hr post-transfection, cells were harvested and lysed for immunoblotting as indicated. The CMV-driven promoter plasmid GFP-NLS-myc was used as a control. The relative density (RD) of protein level of LANA or GFP-NLS is quantified based on triplicate experiments and shown on the right panel. (**C**) STAT6 enhances the protein stability of LANA. HEK293T cells were co-transfected by LANA-myc with full length (FL) FLAG-STAT6, its mutants (Y641F, deletion with amino-domain ΔN or DNA-binding domain ΔDBD) or vector alone. 36hr post-transfection, cells were treated with Cycloheximide (CHX) 20μg/ml for the indicated time before harvesting and lysing for immunoblotting. The relative density (RD) of protein level of LANA is quantified based on triplicate experiments and shown at the right panel.

**Fig 9:**
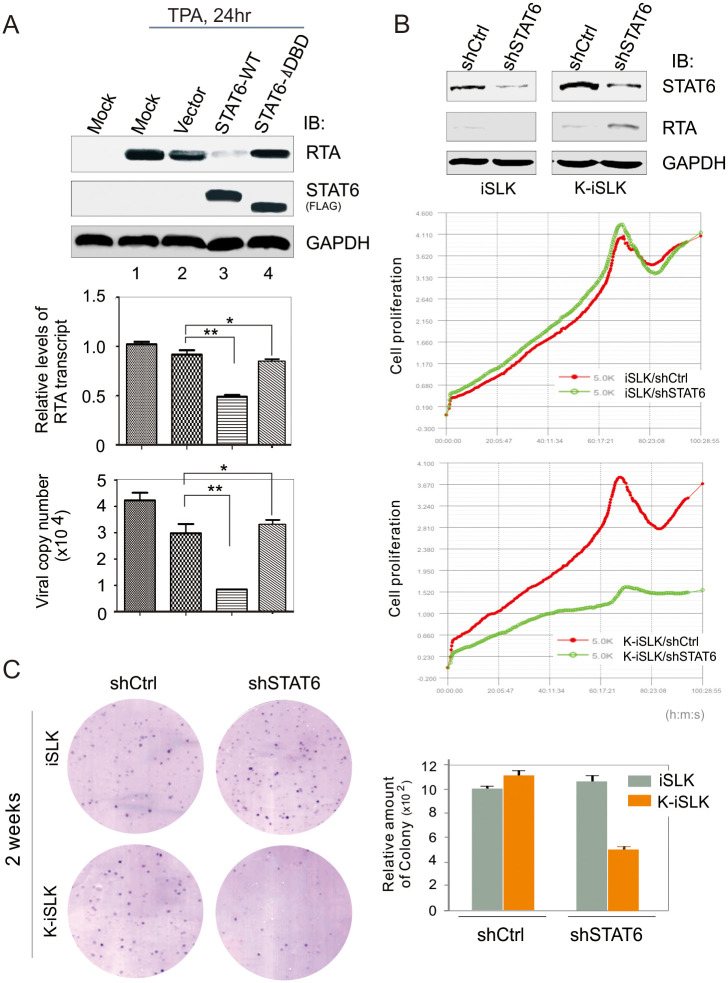
STAT6 is crucial for KSHV to block viral lytic replication and drive cell growth. (**A**) Introduction of intact STAT6 inhibits RTA transcription and virion production. HEK293/Bac36 cells (mock) or HEK293/Bac36 cells transfected with wild-type (WT) or DBD-deleted mutant (ΔDBD) of exogenous STAT6 or vector alone, at 48hr post-transfection, were individually treated with TPA/Sodium butyrate for 24 hr before harvest. Equal amounts of cells were divided for immunoblotting against RTA, STAT6 and GAPDH as indicated in the figure, and RNA extract for quantitative PCR of RTA transcription. The supernatants from culture were purified to quantitate virion production. The statistical significance was evaluated and p<0.05 indicated as double asterisks. (**B**) STAT6 knockdown activates RTA expression and reduces KSHV-infected cell growth. iSLK and K-iSLK cells were individually transfected shSTAT6 or shCtrl control. Immunoblotting analysis of endogenous STAT6 and RTA was carried out at 48hr post-transfection. Equal amounts of transfected cells were seeded for real-time monitoring of cell proliferation over a four-day period (bottom panel). (**C**) STAT6 knockdown reduces KSHV-infected cell colony formation. A representative of colony formation after 2-week culture is shown. The relative amount of colony was calculated from two independent experiments (right panel).

## Supporting information

S1 Fig HEK293 cells transfected with truncated mutants (ΔN, ΔTAD, ΔDBD) of STAT6 with FLAG tag (green) in the presence or absence of LANA with RFP tag (red) imaged by confocal assay. Nuclei were stained with DAPI. The relative percentage of STAT6 nuclear localization (bottom panel) was individually quantified by nuclear and cytoplasmic staining of 100 cells.
(TIF)

S1 File Original data underlying results presented in Fig 3. (ZIP)

S2 File Original data underlying results presented in Fig 5. (ZIP)

S3 File Original data underlying results presented in Fig 6. (ZIP)

S4 File Original data underlying results presented in Fig 9. (ZIP)

S5 File Original data underlying results presented in S1 Fig. (ZIP)

S6 File Underlying individual level data for Figs 1, 7, and 8. (ZIP)

S7 File Underlying blots and images for Figs 1–9. (PDF)
