# Supplementary figures and images for: Nuclear Localization and Cleavage of STAT6 Is Induced by Kaposi’s Sarcoma-Associated Herpesvirus for Viral Latency
Source: PLoS Pathog. 2017 Jan 18;13(1):e1006124. doi: 10.1371/journal.ppat.1006124 (PMC5242515; doi:10.1371/journal.ppat.1006124)

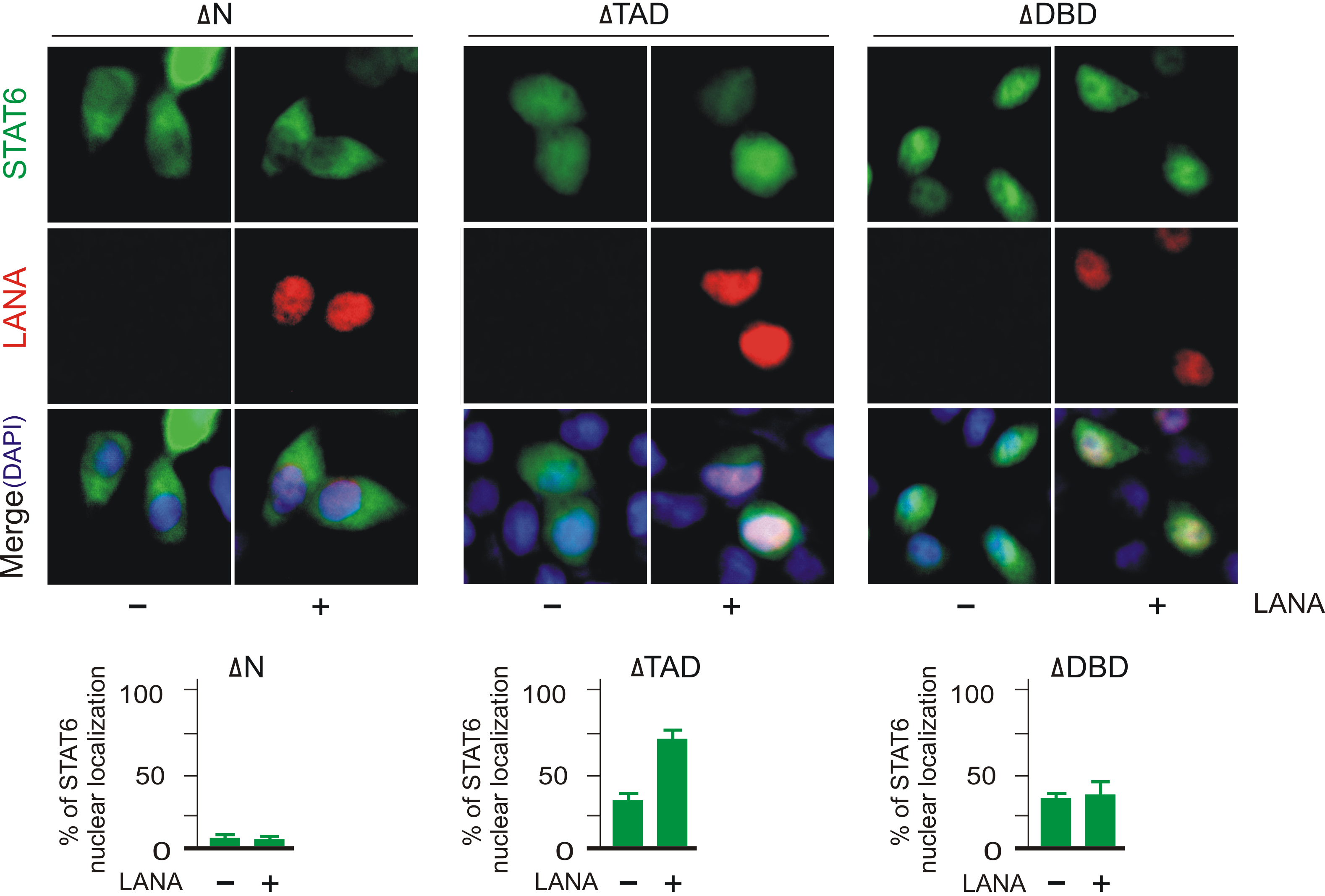

Supplement: S1 Fig — Nuclei were stained with DAPI. The relative percentage of STAT6 nuclear localization (bottom panel) was individually quantified by nuclear and cytoplasmic staining of 100 cells. (TIF) [file ppat.1006124.s001.tif]

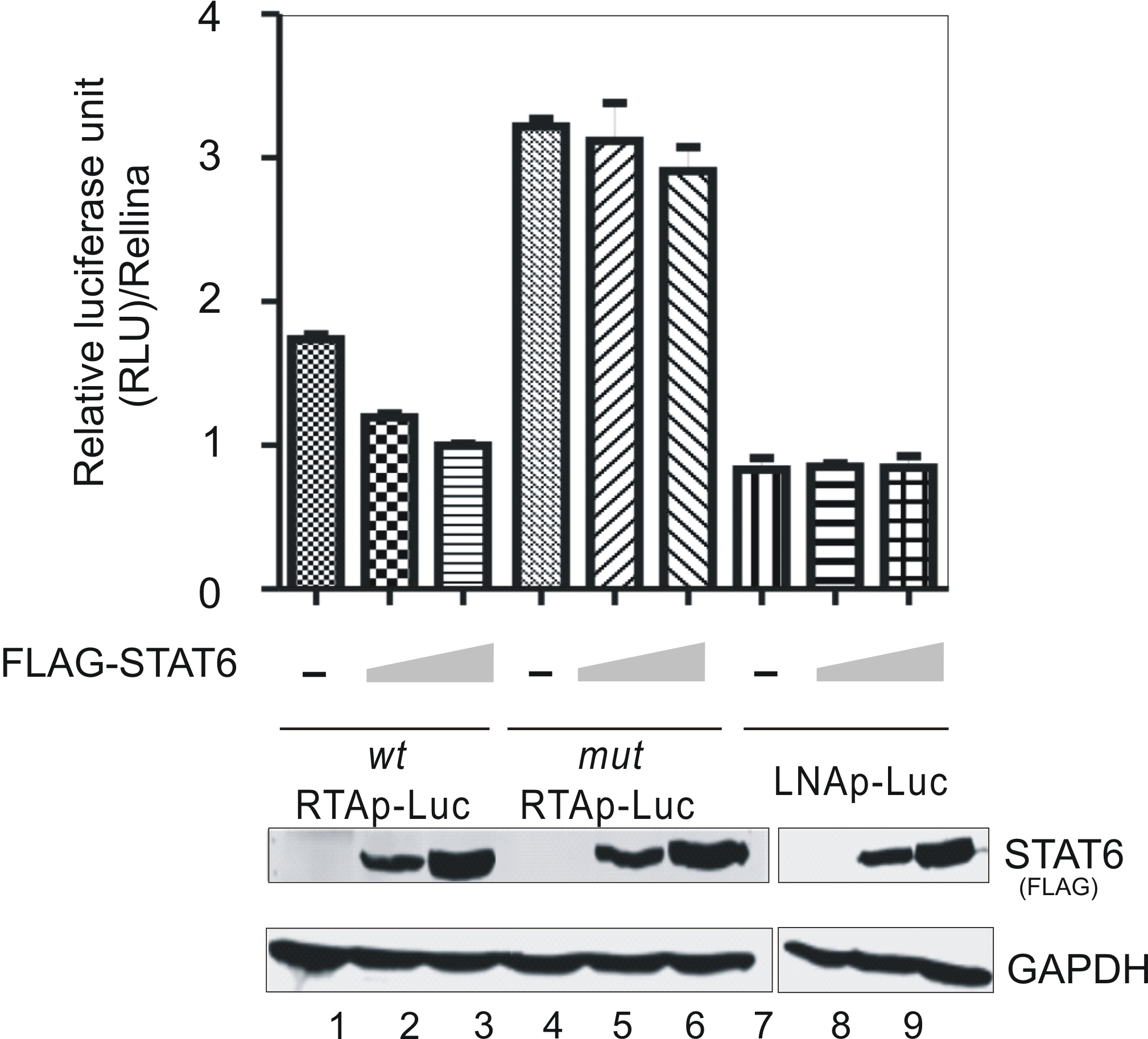

Supplement: S2 Fig — HEK293 cells co-transfected with the indicated promoter-reporters at different dosages (0, 2, 5μg) of FLAG-STAT6 were subjected to reporter assay. Relative luciferase unit (RLU) normalization with Rellina activity was analyzed. Data are means ±SEM. Immunoblotting analyses of exogenous STAT6 is shown in the bottom panel. GAPDH was used as control. (TIF) [file ppat.1006124.s002.tif]

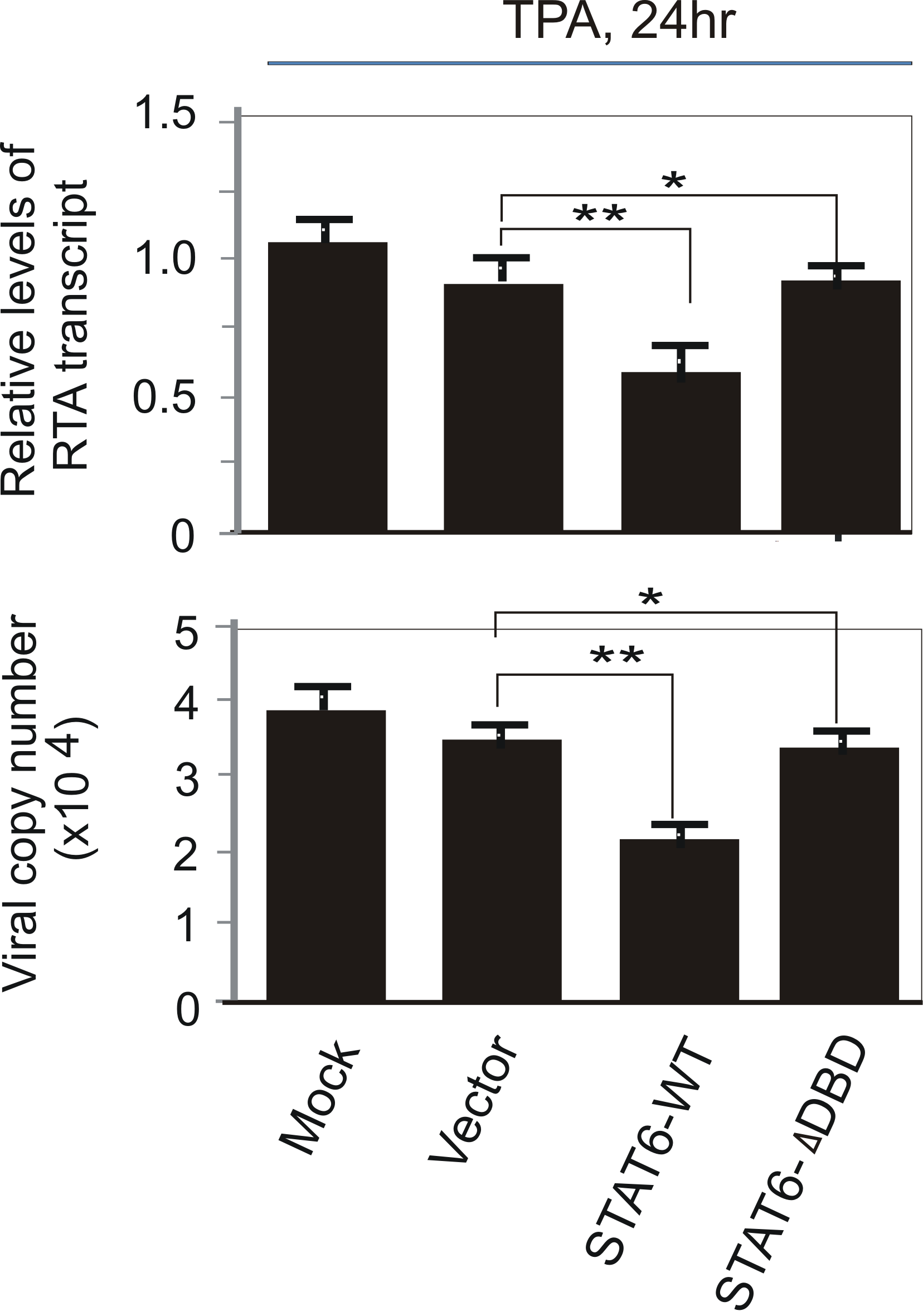

Supplement: S3 Fig — K-iSLK cells (mock) or K-iSLK cells transfected with wild-type (WT) or DBD-deleted mutant (ΔDBD) of exogenous STAT6 or vector alone, at 48hr post-transfection, were individually treated with TPA/Sodium butyrate for 24 hr before harvest. Equal amounts of cells were used to RNA extract for quantitative PCR of RTA transcription. The supernatants from culture were purified to quantitate virion production. The statistical significance was evaluated and p<0.05 indicated as double asterisks. (TIF) [file ppat.1006124.s003.tif]

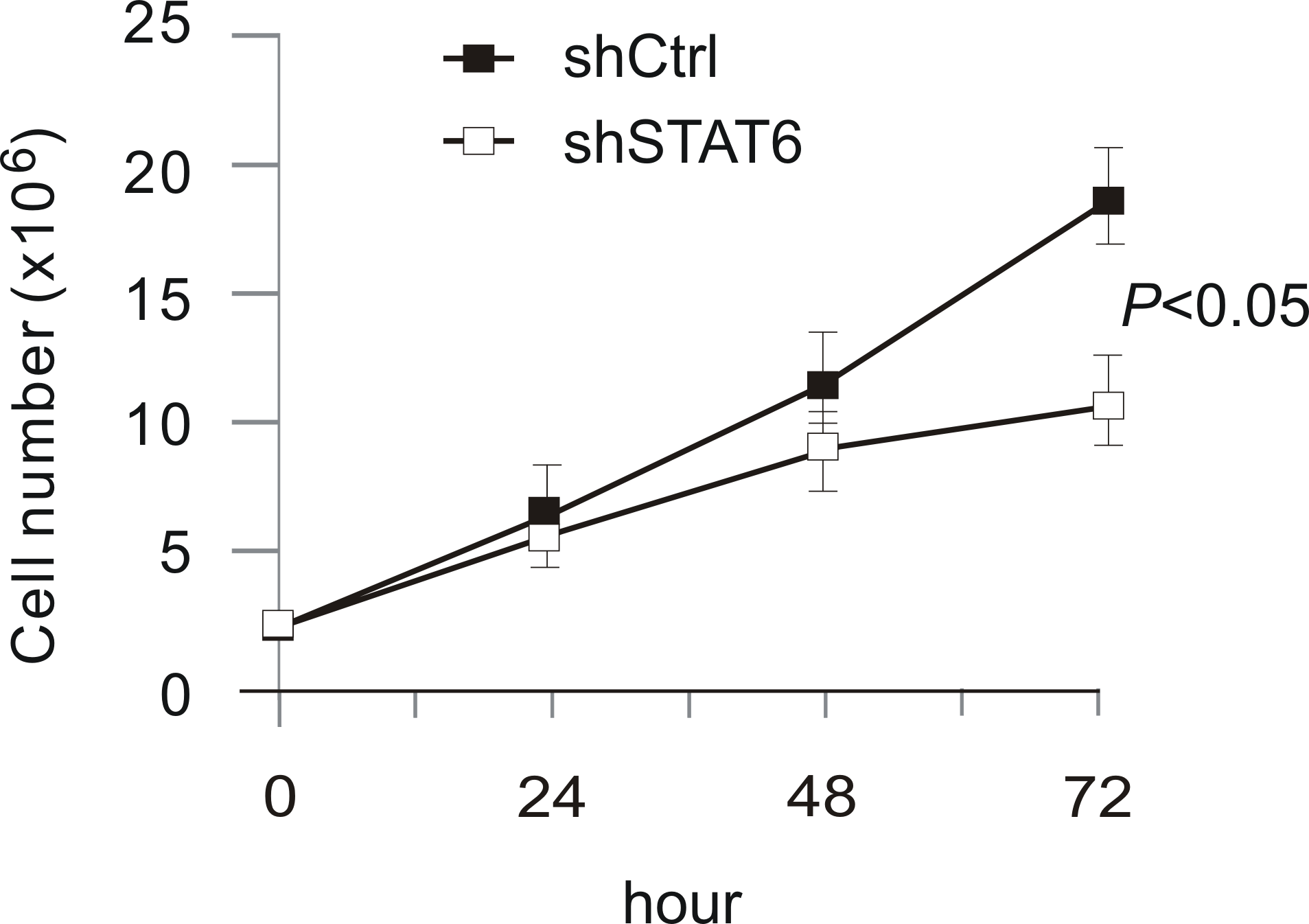

Supplement: S4 Fig — PEL BC3 cells were individually transfected shSTAT6 or shCtrl control. Equal amounts (2.5 million) of transfected cells were seeded and monitored the cell growth for 3 days. (TIF) [file ppat.1006124.s004.tif]
