# Supplementary figures and images for: Expression of Concern: Nuclear Localization and Cleavage of STAT6 Is Induced by Kaposi’s Sarcoma-Associated Herpesvirus for Viral Latency
Source: PLoS Pathog. 2021 Dec 15;17(12):e1010047. doi: 10.1371/journal.ppat.1010047 (PMC8673609; doi:10.1371/journal.ppat.1010047)

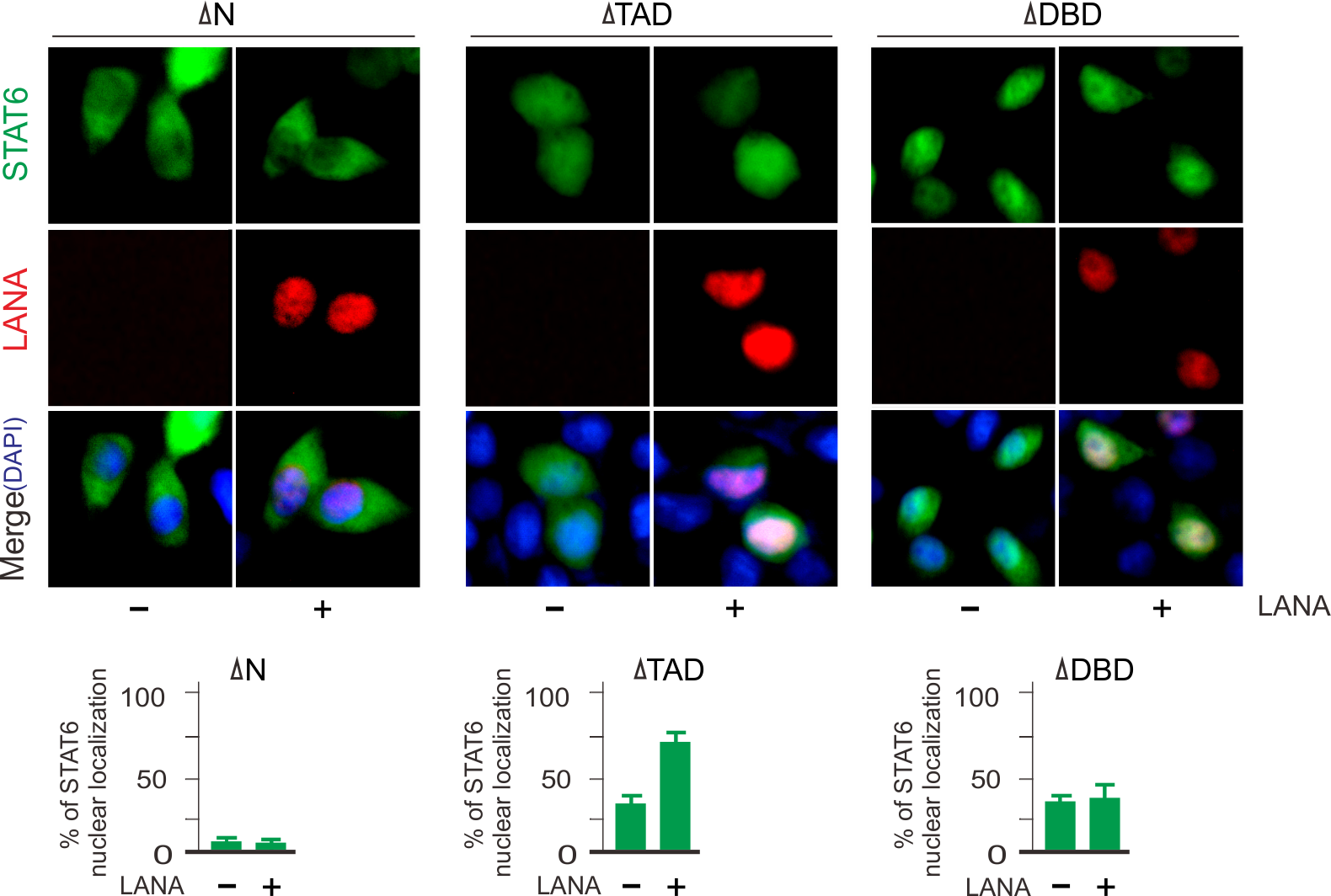

Supplement: S1 Fig — Nuclei were stained with DAPI. The relative percentage of STAT6 nuclear localization (bottom panel) was individually quantified by nuclear and cytoplasmic staining of 100 cells. (TIF) [file ppat.1010047.s001.tif]

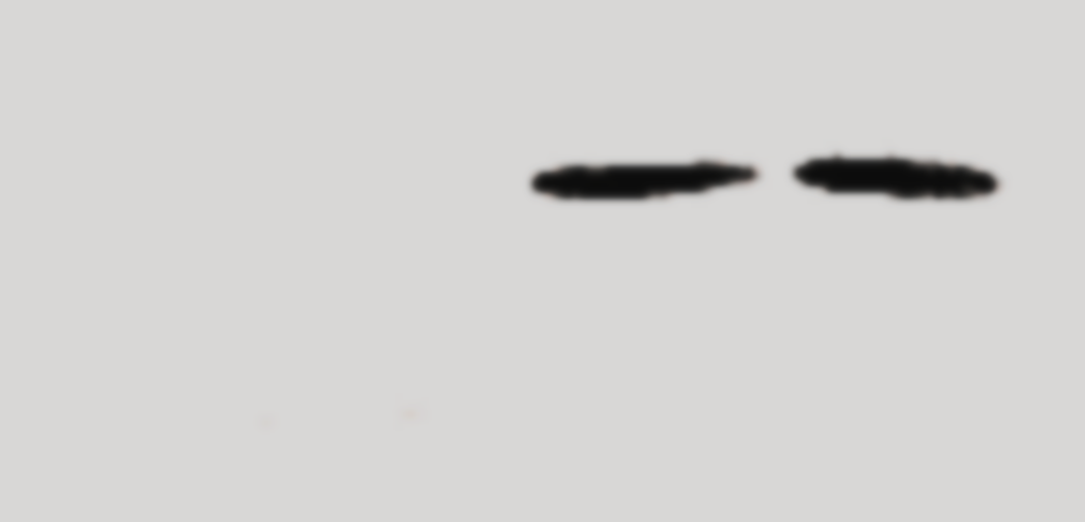

Supplement: S1 File — (ZIP) [file ppat.1010047.s002.zip › S1 File underlying data Figure 3/Fig.3B-IB-Histone H3.tif]

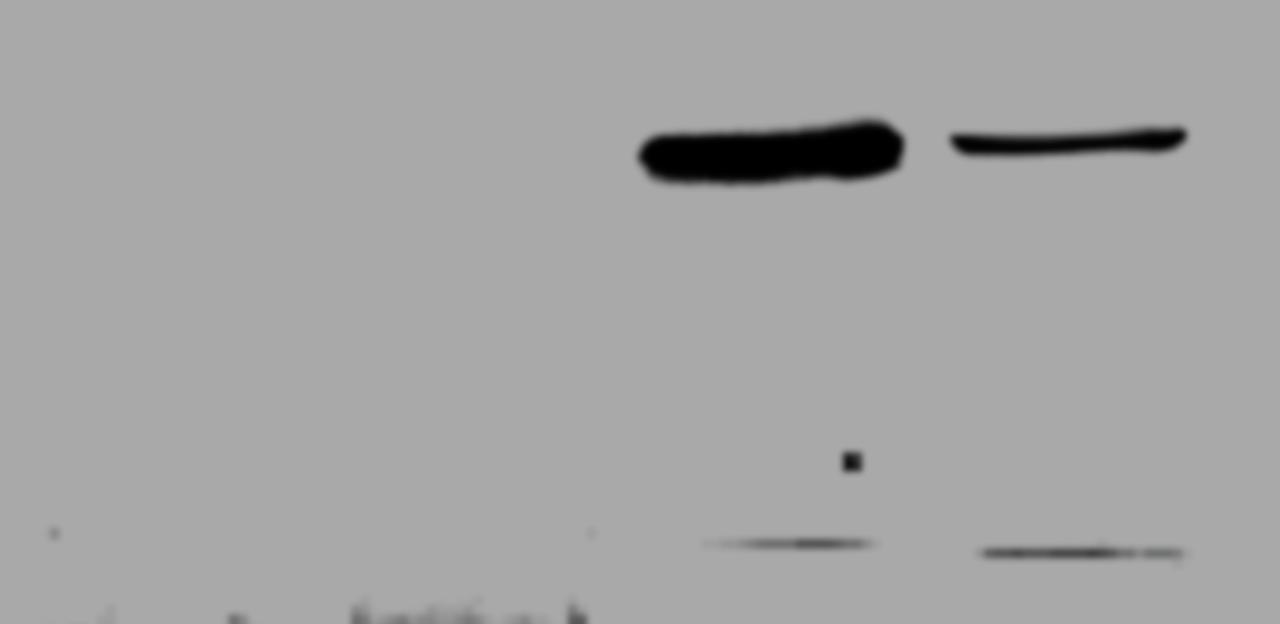

Supplement: S1 File — (ZIP) [file ppat.1010047.s002.zip › S1 File underlying data Figure 3/Fig.3B-IB-LANA.tif]

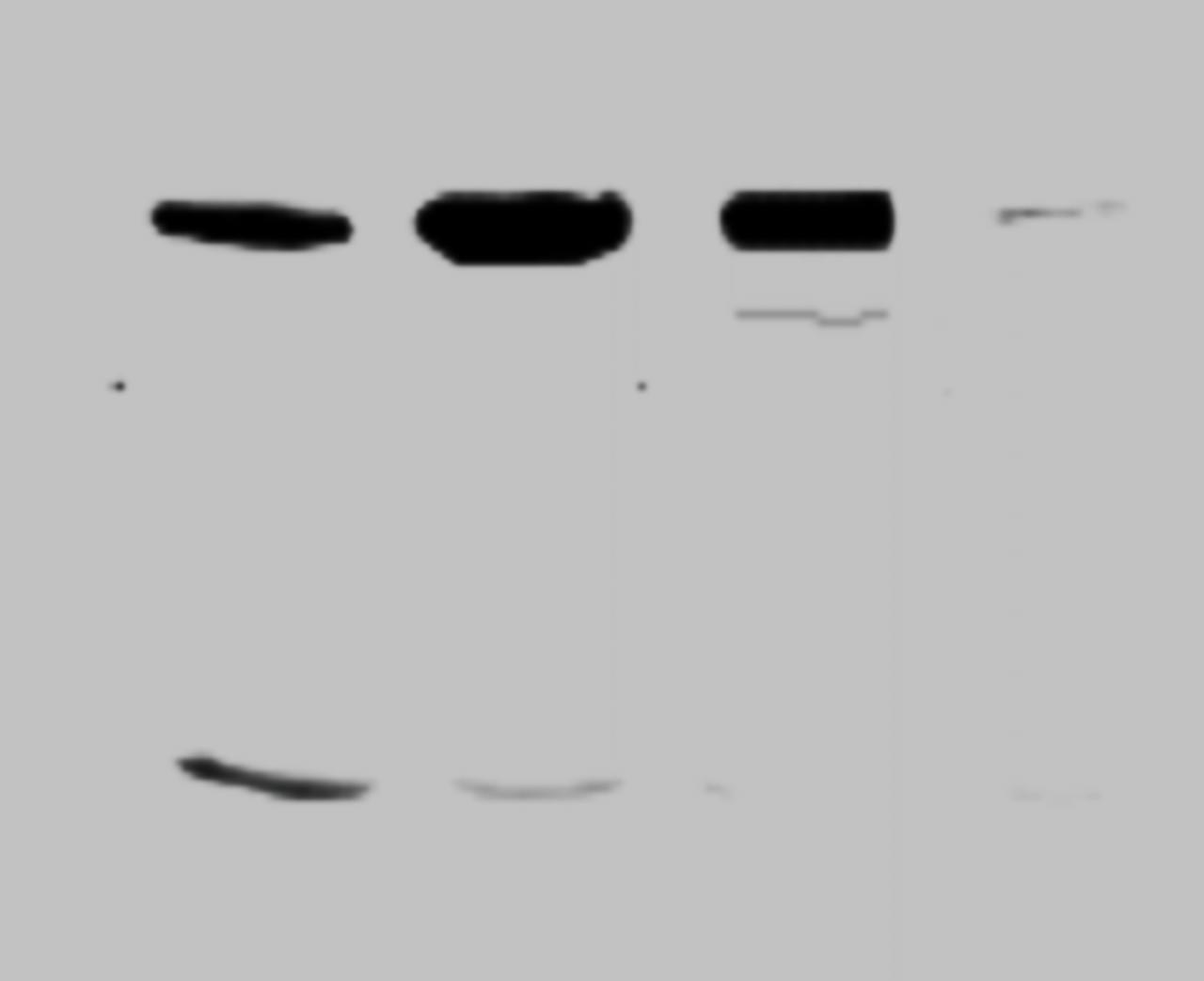

Supplement: S1 File — (ZIP) [file ppat.1010047.s002.zip › S1 File underlying data Figure 3/Fig.3B-IB-STAT6.tif]

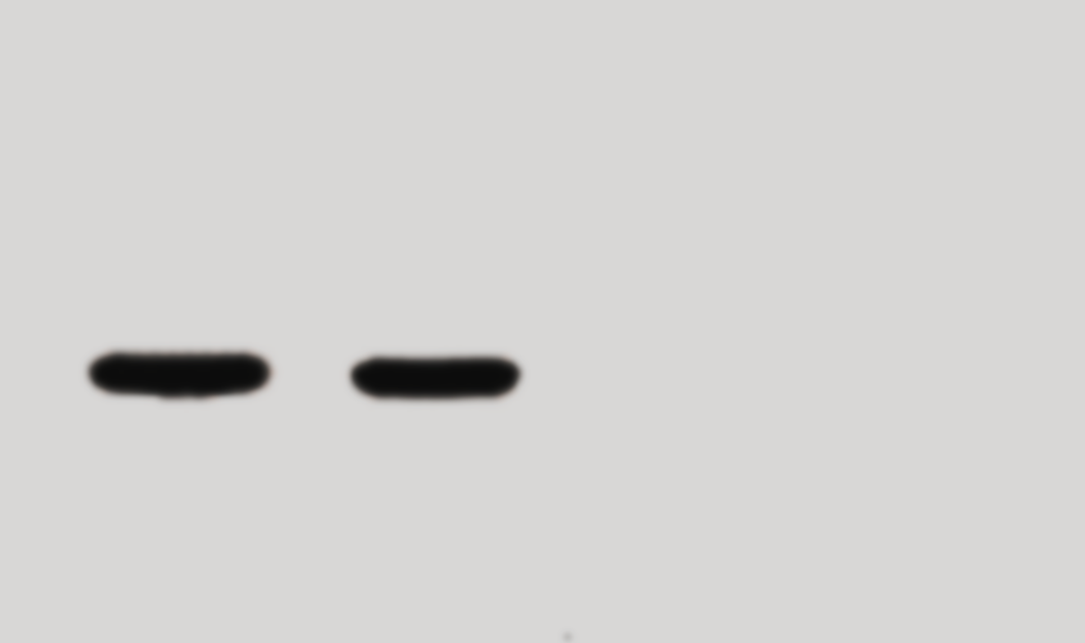

Supplement: S1 File — (ZIP) [file ppat.1010047.s002.zip › S1 File underlying data Figure 3/Fig.3B-IB-Tubulin.tif]

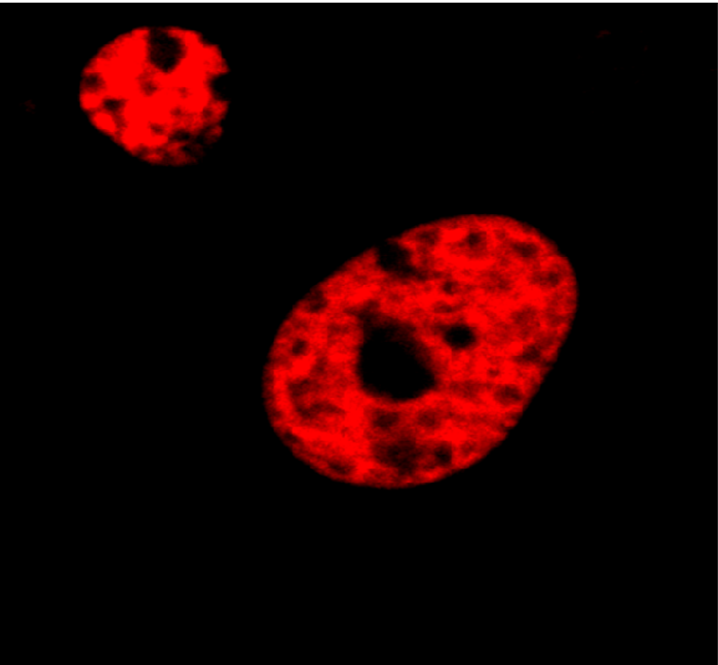

Supplement: S1 File — (ZIP) [file ppat.1010047.s002.zip › S1 File underlying data Figure 3/Fig.3C-STAT6 WT+LANA.tif]

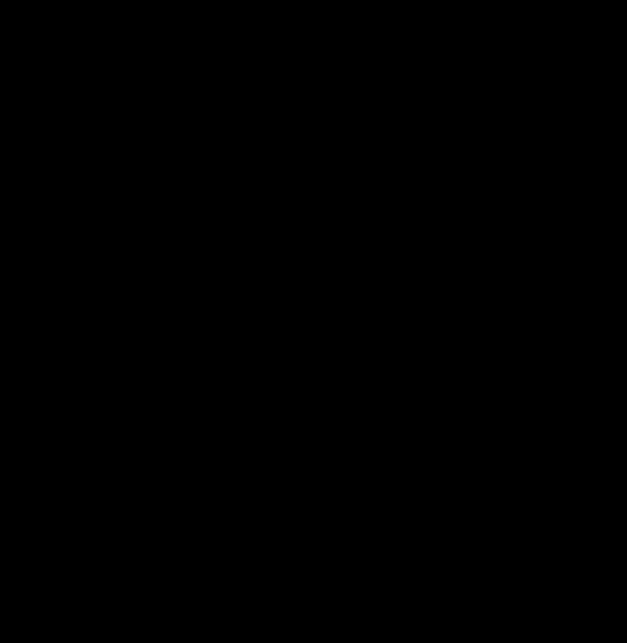

Supplement: S1 File — (ZIP) [file ppat.1010047.s002.zip › S1 File underlying data Figure 3/Fig.3C-STAT6 WT-LANA1.tif]

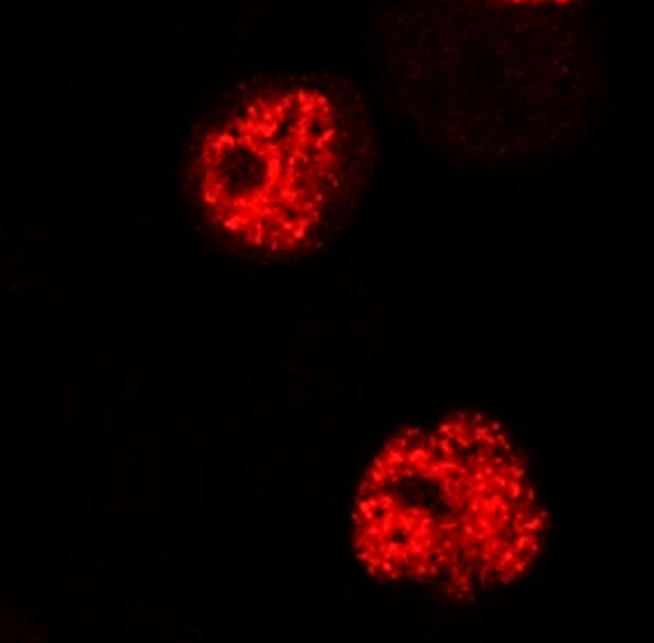

Supplement: S1 File — (ZIP) [file ppat.1010047.s002.zip › S1 File underlying data Figure 3/Fig.3C-Y641F+LANA.tif]

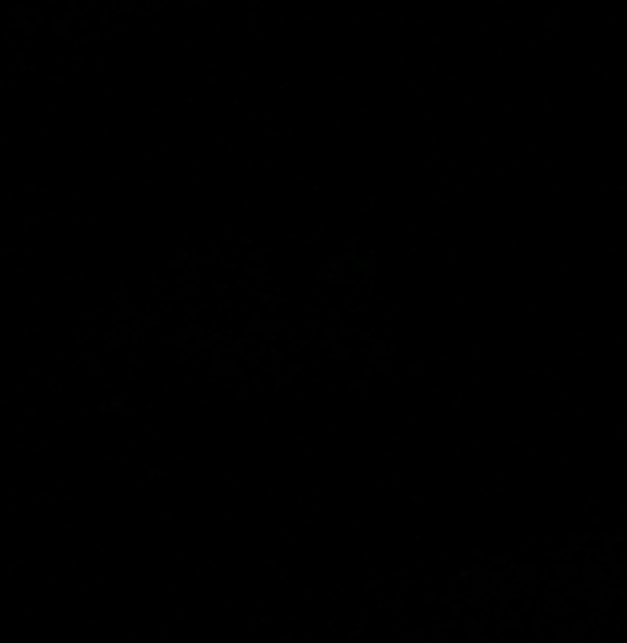

Supplement: S1 File — (ZIP) [file ppat.1010047.s002.zip › S1 File underlying data Figure 3/Fig.3C-Y641F-LANA.tif]

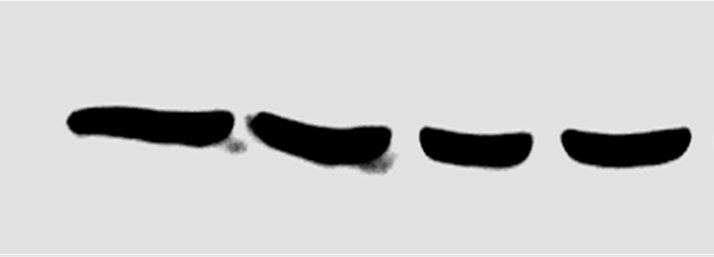

Supplement: S2 File — (ZIP) [file ppat.1010047.s003.zip › S2 File underlying data Figure 5/Fig.5C-STAT6 dDBD-IB-GAPDH.tif]

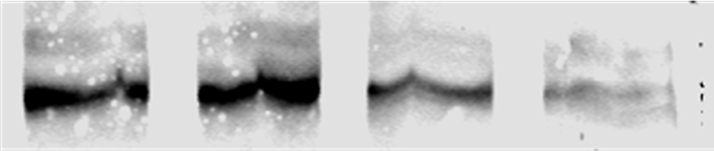

Supplement: S2 File — (ZIP) [file ppat.1010047.s003.zip › S2 File underlying data Figure 5/Fig.5C-STAT6 dDBD-IB-LANA.tif]

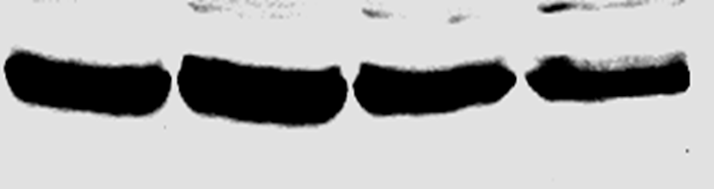

Supplement: S2 File — (ZIP) [file ppat.1010047.s003.zip › S2 File underlying data Figure 5/Fig.5C-STAT6 dDBD-IB-STAT6.tif]

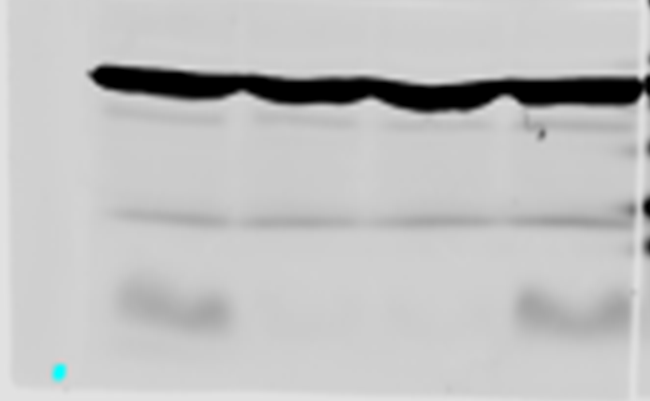

Supplement: S2 File — (ZIP) [file ppat.1010047.s003.zip › S2 File underlying data Figure 5/Fig.5C-STAT6 dN-IB-GAPDH.tif]

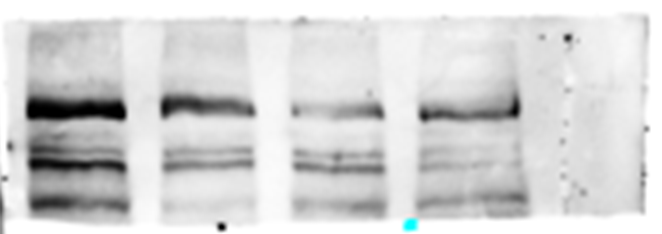

Supplement: S2 File — (ZIP) [file ppat.1010047.s003.zip › S2 File underlying data Figure 5/Fig.5C-STAT6 dN-IB-LANA.tif]

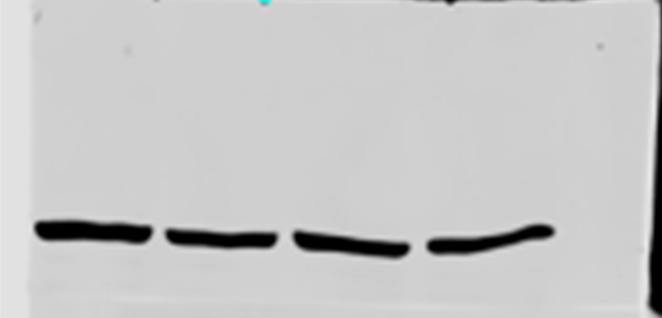

Supplement: S2 File — (ZIP) [file ppat.1010047.s003.zip › S2 File underlying data Figure 5/Fig.5C-STAT6 dN-IB-STAT6.tif]

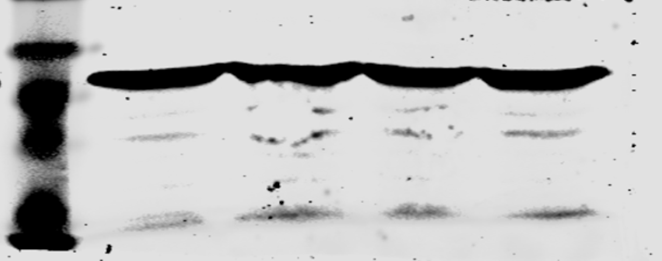

Supplement: S2 File — (ZIP) [file ppat.1010047.s003.zip › S2 File underlying data Figure 5/Fig.5C-STAT6 Y641F-IB-GAPDH.tif]

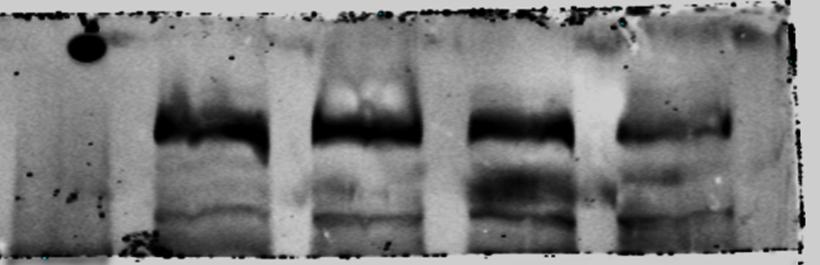

Supplement: S2 File — (ZIP) [file ppat.1010047.s003.zip › S2 File underlying data Figure 5/Fig.5C-STAT6 Y641F-IB-LANA.tif]

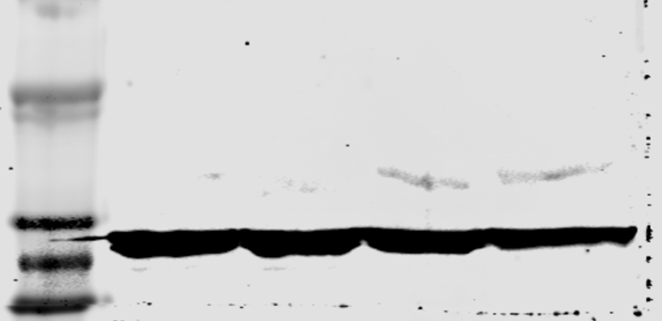

Supplement: S2 File — (ZIP) [file ppat.1010047.s003.zip › S2 File underlying data Figure 5/Fig.5C-STAT6 Y641F-IB-STAT6.tif]

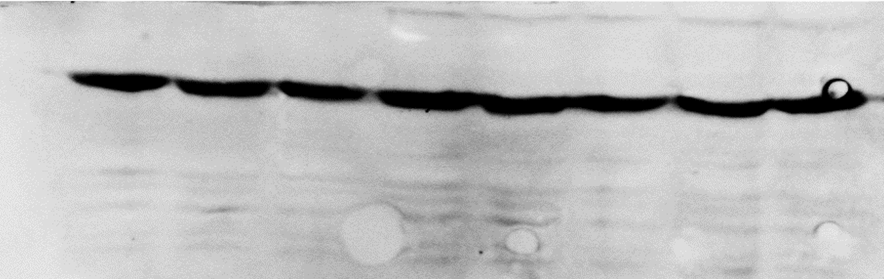

Supplement: S2 File — (ZIP) [file ppat.1010047.s003.zip › S2 File underlying data Figure 5/Fig.5C-vector-STAT6 FL-IB-GAPDH.tif]

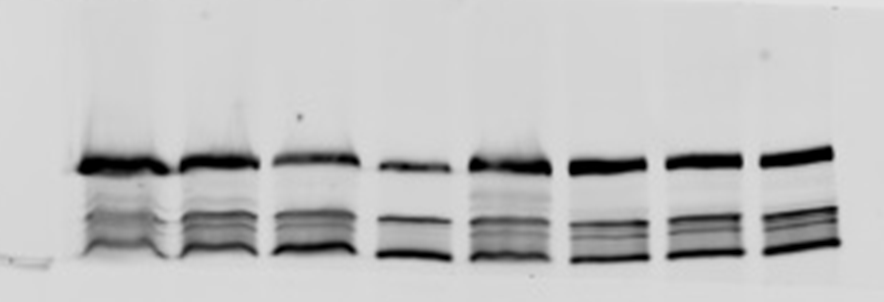

Supplement: S2 File — (ZIP) [file ppat.1010047.s003.zip › S2 File underlying data Figure 5/Fig.5C-vector-STAT6 FL-IB-LANA.tif]

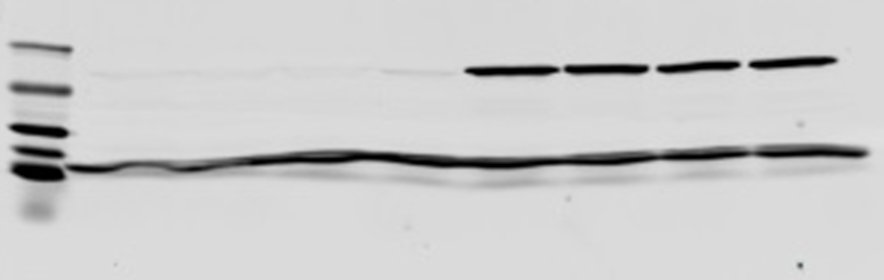

Supplement: S2 File — (ZIP) [file ppat.1010047.s003.zip › S2 File underlying data Figure 5/Fig.5C-vector-STAT6 FL-IB-STAT6.tif]

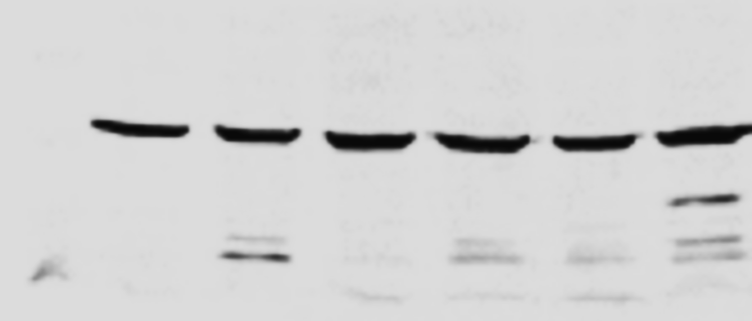

Supplement: S3 File — (ZIP) [file ppat.1010047.s004.zip › S3 File underlying data Figure 6/Fig.6B-FLAG-STAT6-IB-GAPDH.tif]

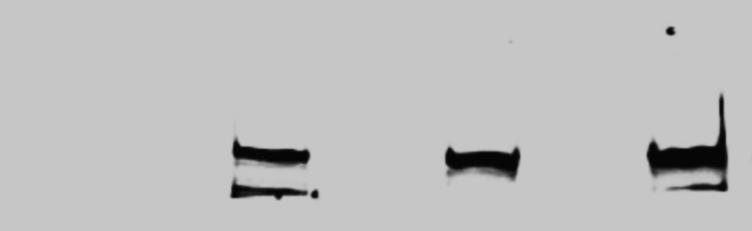

Supplement: S3 File — (ZIP) [file ppat.1010047.s004.zip › S3 File underlying data Figure 6/Fig.6B-FLAG-STAT6-IB-LANA.tif]

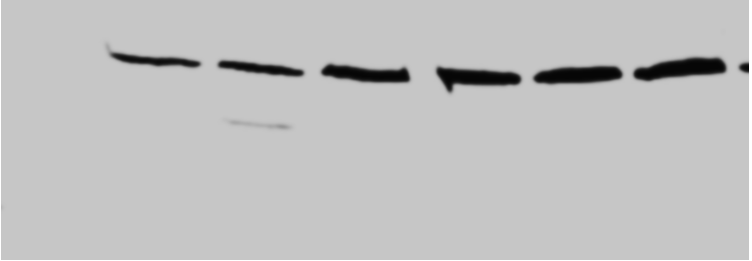

Supplement: S3 File — (ZIP) [file ppat.1010047.s004.zip › S3 File underlying data Figure 6/Fig.6B-FLAG-STAT6-IB-STAT6.tif]

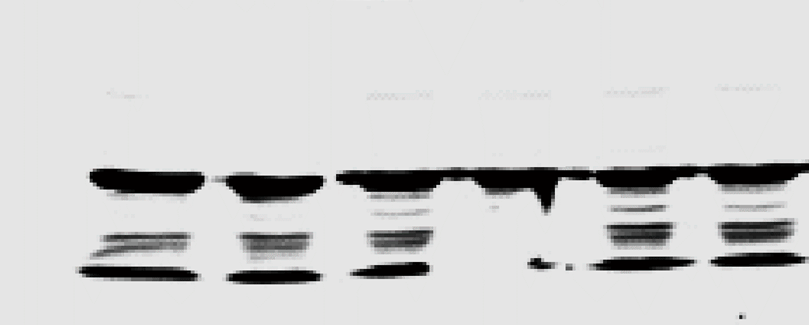

Supplement: S3 File — (ZIP) [file ppat.1010047.s004.zip › S3 File underlying data Figure 6/Fig.6B-HA-STAT3-IB-GAPDH.tif]

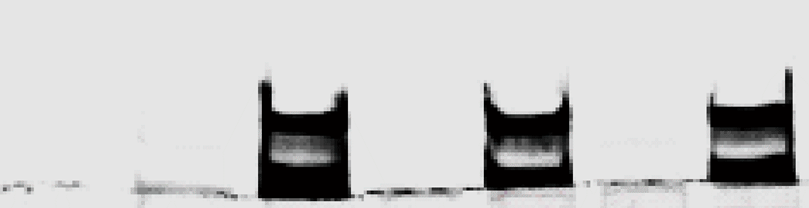

Supplement: S3 File — (ZIP) [file ppat.1010047.s004.zip › S3 File underlying data Figure 6/Fig.6B-HA-STAT3-IB-LANA.tif]

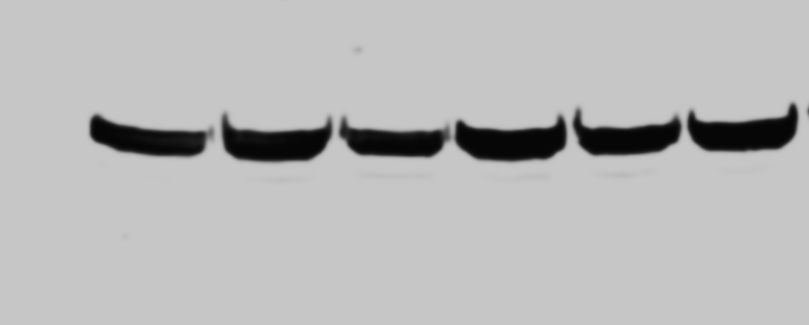

Supplement: S3 File — (ZIP) [file ppat.1010047.s004.zip › S3 File underlying data Figure 6/Fig.6B-HA-STAT3-IB-STAT6.tif]

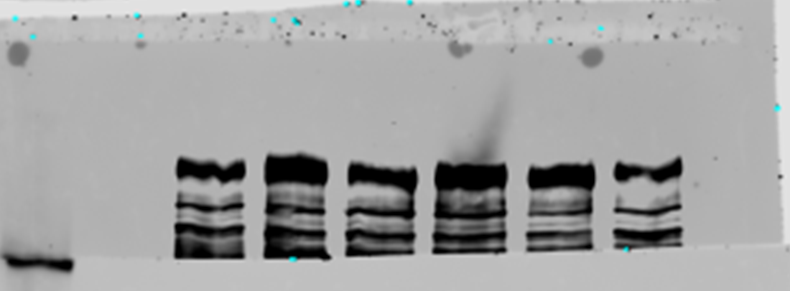

Supplement: S3 File — (ZIP) [file ppat.1010047.s004.zip › S3 File underlying data Figure 6/Fig.6C-FLAG-STAT6-+LANA-IB-LANA.tif]

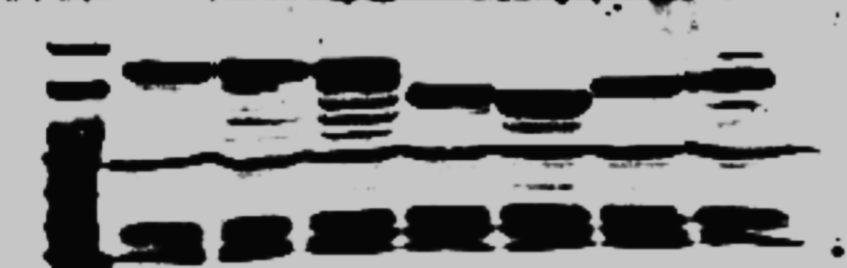

Supplement: S3 File — (ZIP) [file ppat.1010047.s004.zip › S3 File underlying data Figure 6/Fig.6C-FLAG-STAT6-+LANA-IB-STAT6.tif]

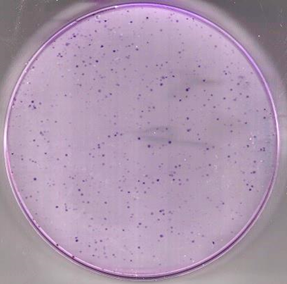

Supplement: S4 File — (ZIP) [file ppat.1010047.s005.zip › S4 File underlying data Figure 9/Fig.9C-iSLK-shCtrl.tif]

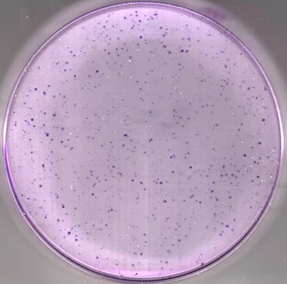

Supplement: S4 File — (ZIP) [file ppat.1010047.s005.zip › S4 File underlying data Figure 9/Fig.9C-iSLK-shSTAT6.tif]

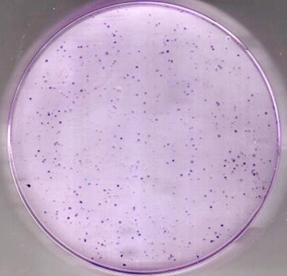

Supplement: S4 File — (ZIP) [file ppat.1010047.s005.zip › S4 File underlying data Figure 9/Fig.9C-K-iSLK-shCtrl.tif]

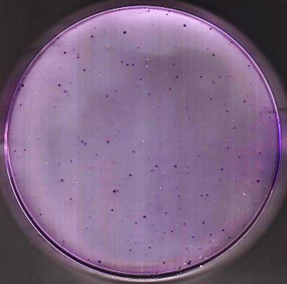

Supplement: S4 File — (ZIP) [file ppat.1010047.s005.zip › S4 File underlying data Figure 9/Fig.9C-K-iSLK-shSTAT6.tif]

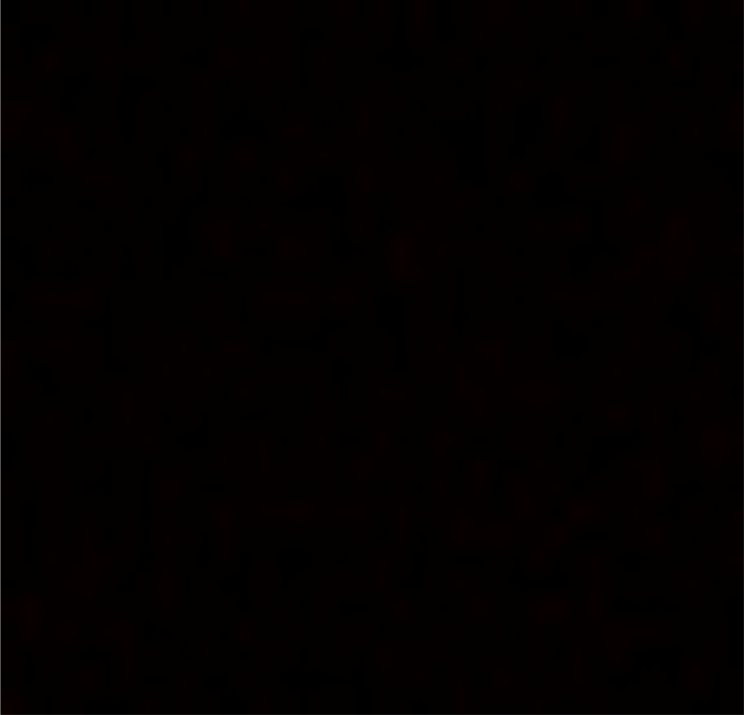

Supplement: S5 File — (ZIP) [file ppat.1010047.s006.zip › S5 File underlying data Figure S1/Figure S1-dDBD-.tif]

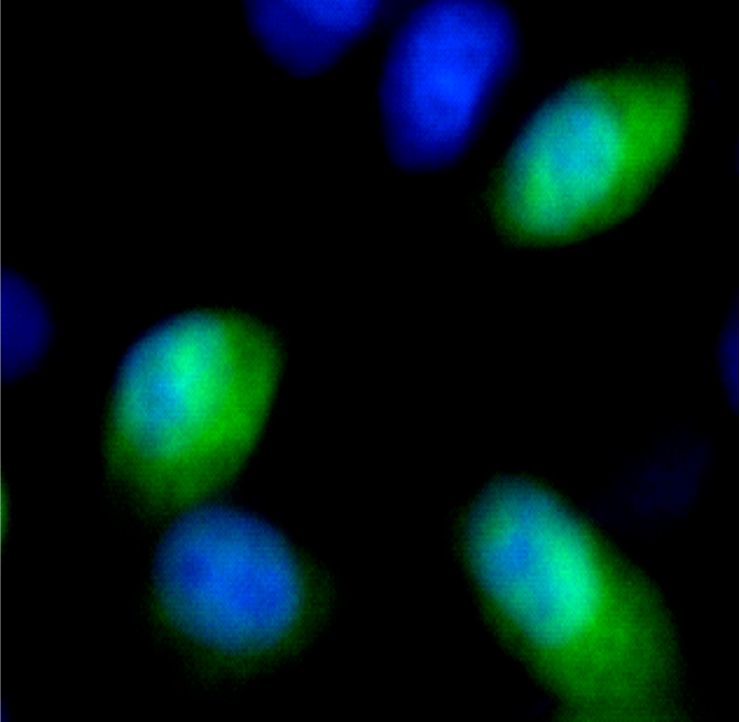

Supplement: S5 File — (ZIP) [file ppat.1010047.s006.zip › S5 File underlying data Figure S1/Figure S1-dDBD-Merge-DAPI.tif]

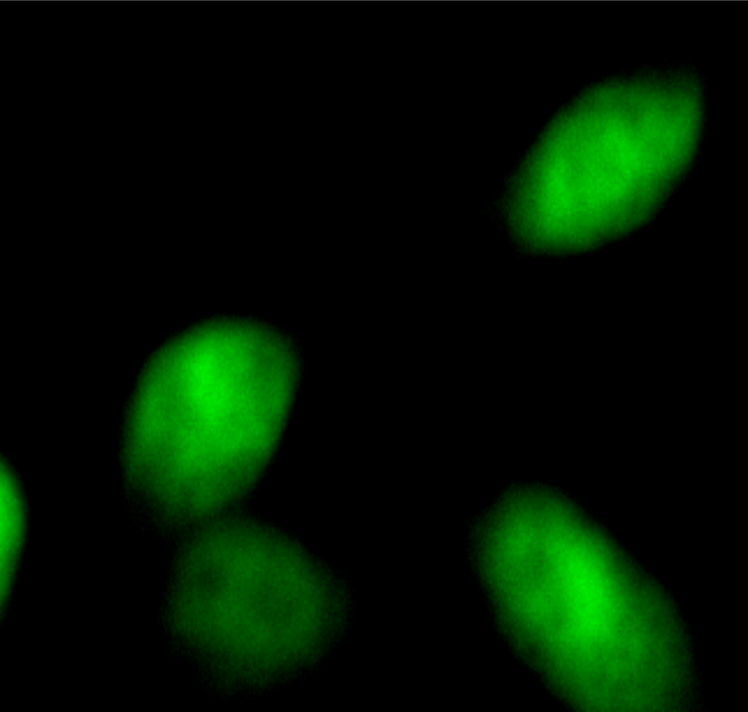

Supplement: S5 File — (ZIP) [file ppat.1010047.s006.zip › S5 File underlying data Figure S1/Figure S1-dDBD-STAT6.tif]

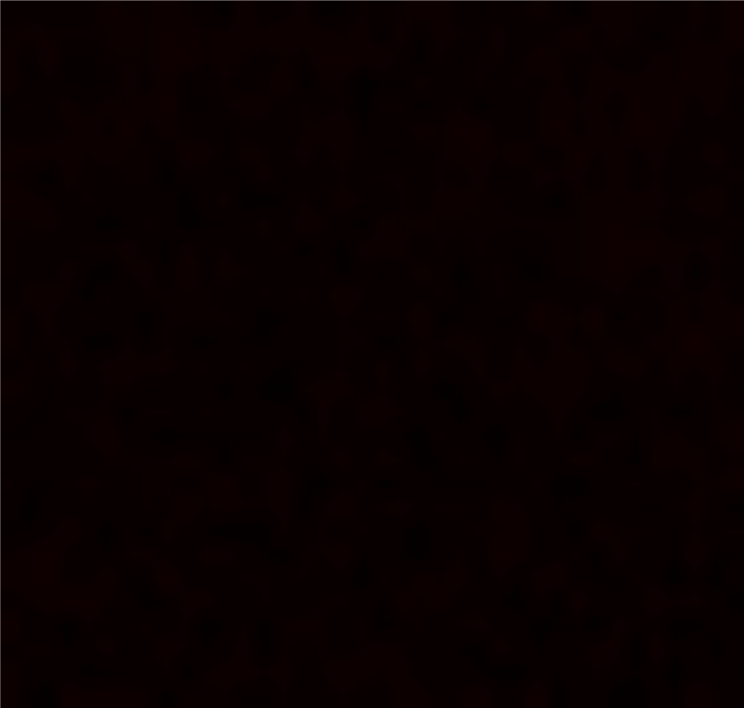

Supplement: S5 File — (ZIP) [file ppat.1010047.s006.zip › S5 File underlying data Figure S1/Figure S1-dN-.tif]

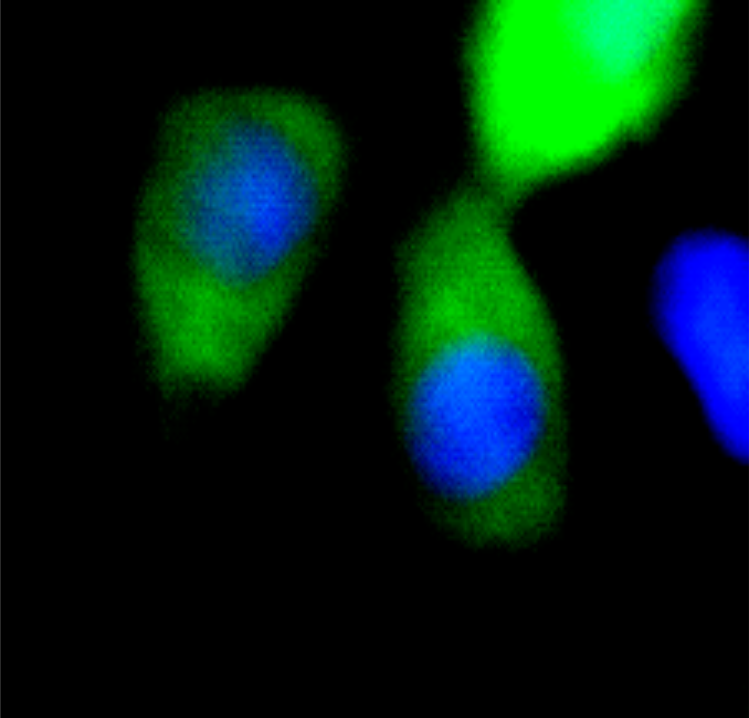

Supplement: S5 File — (ZIP) [file ppat.1010047.s006.zip › S5 File underlying data Figure S1/Figure S1-dN-Merge-DAPI.tif]

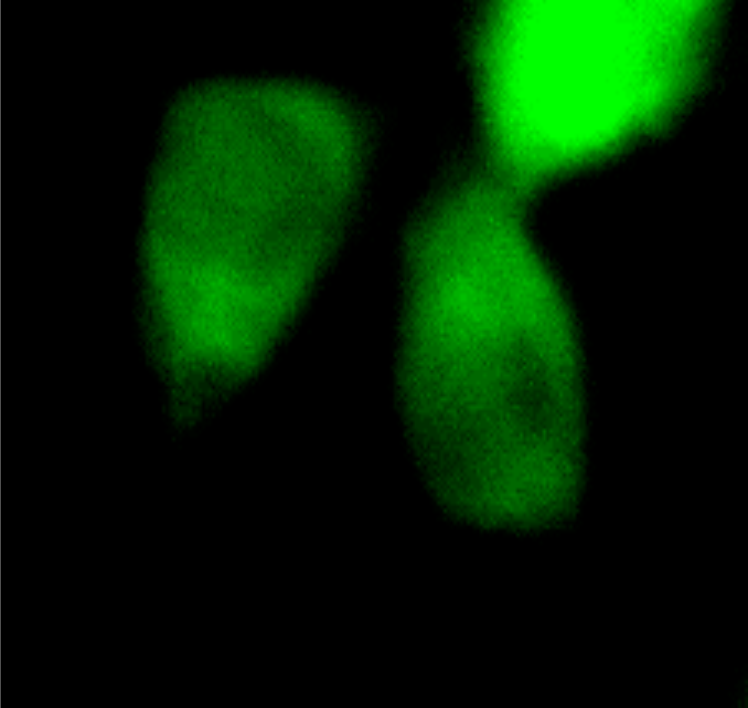

Supplement: S5 File — (ZIP) [file ppat.1010047.s006.zip › S5 File underlying data Figure S1/Figure S1-dN-STAT6.tif]

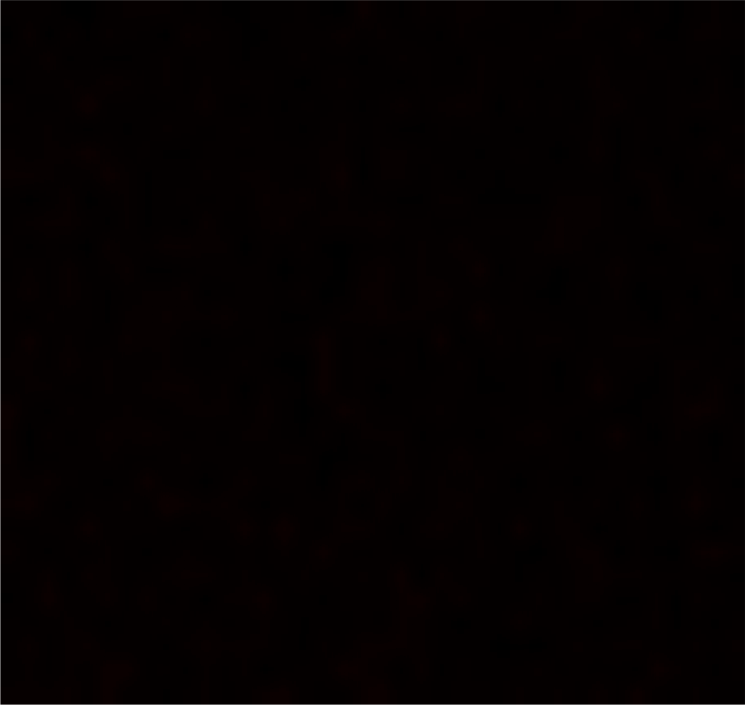

Supplement: S5 File — (ZIP) [file ppat.1010047.s006.zip › S5 File underlying data Figure S1/Figure S1-dTAD-.tif]

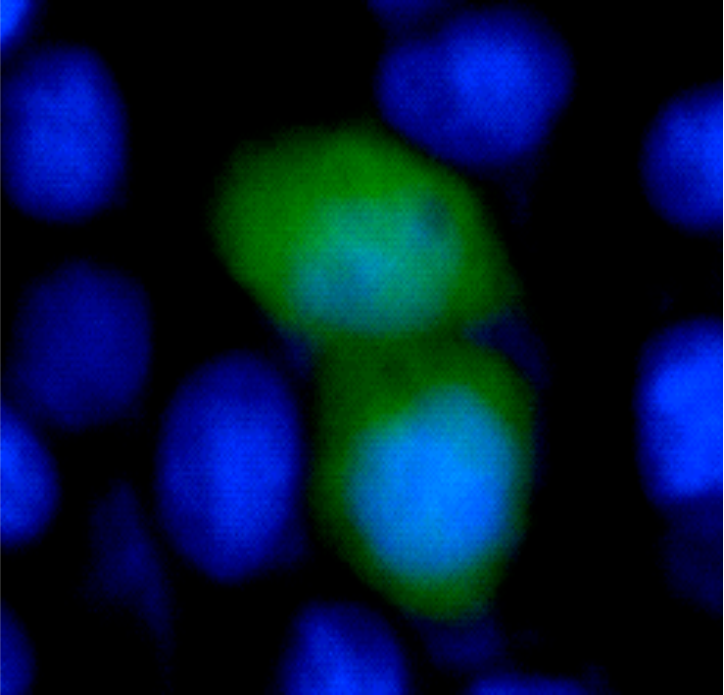

Supplement: S5 File — (ZIP) [file ppat.1010047.s006.zip › S5 File underlying data Figure S1/Figure S1-dTAD-Merge-DAPI.tif]

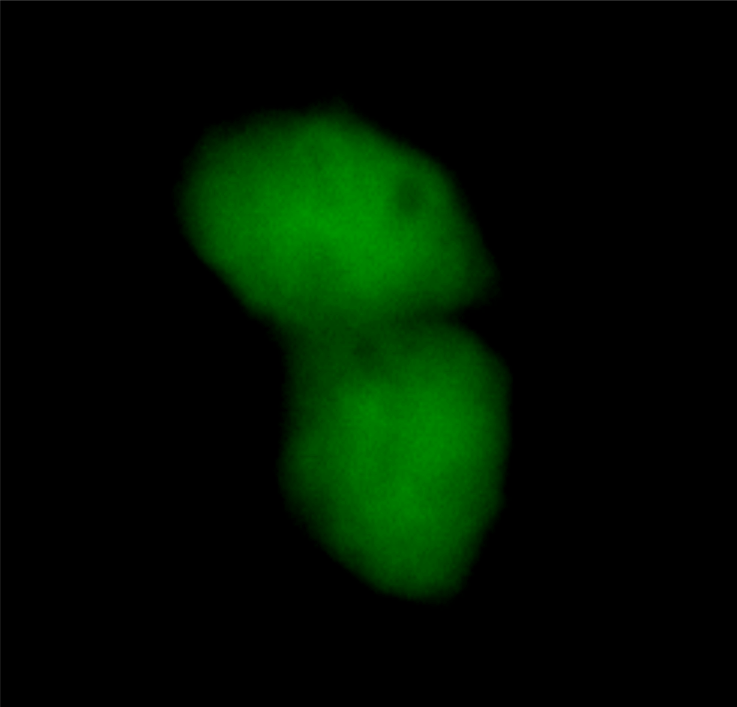

Supplement: S5 File — (ZIP) [file ppat.1010047.s006.zip › S5 File underlying data Figure S1/Figure S1-dTAD-STAT6.tif]
