# Supplementary material for: Expression of Concern: Nuclear Localization and Cleavage of STAT6 Is Induced by Kaposi’s Sarcoma-Associated Herpesvirus for Viral Latency
Source: PLoS Pathog. 2021 Dec 15;17(12):e1010047. doi: 10.1371/journal.ppat.1010047 (PMC8673609; doi:10.1371/journal.ppat.1010047)

Figure 1A

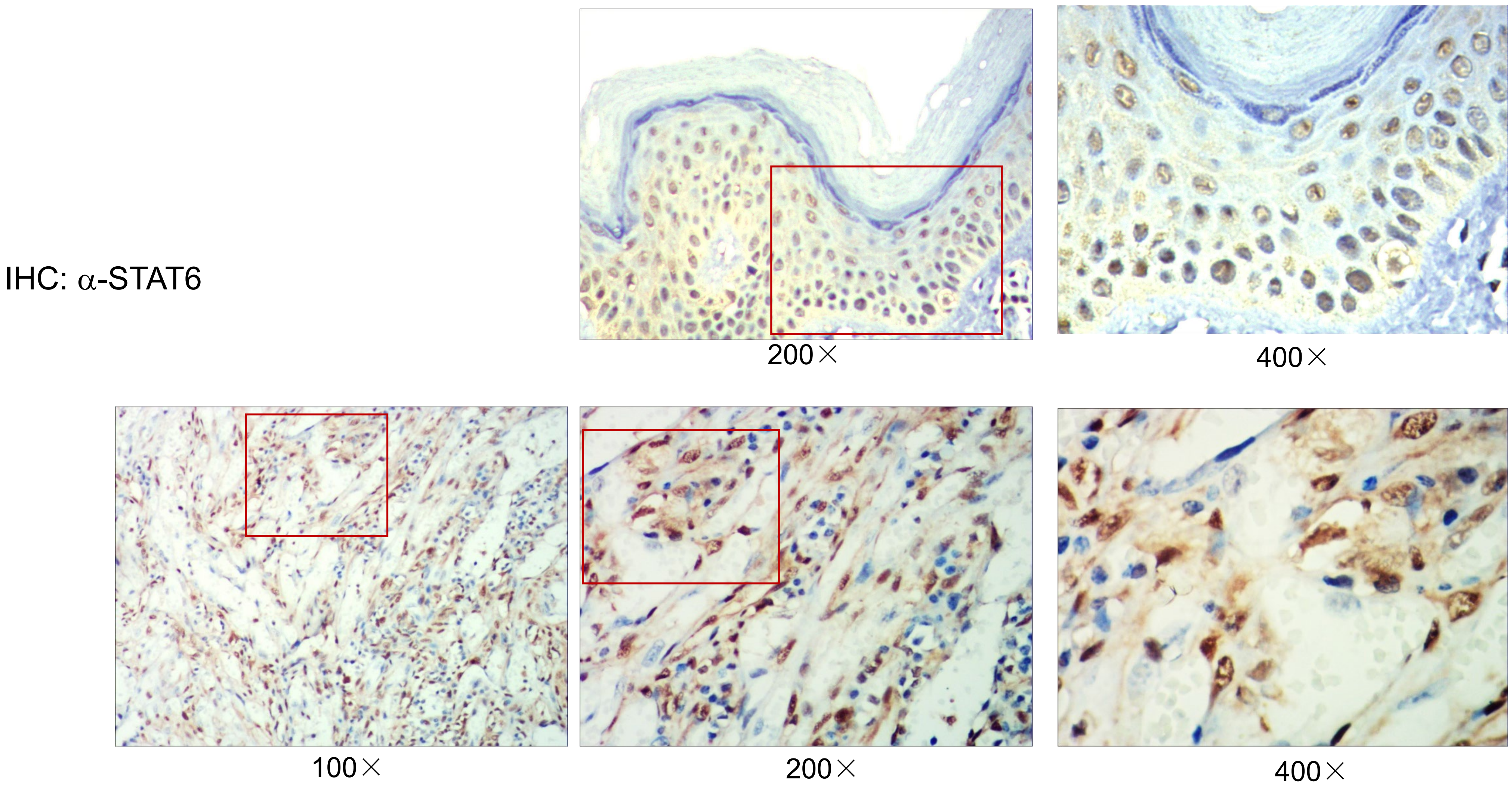

Figure 1C

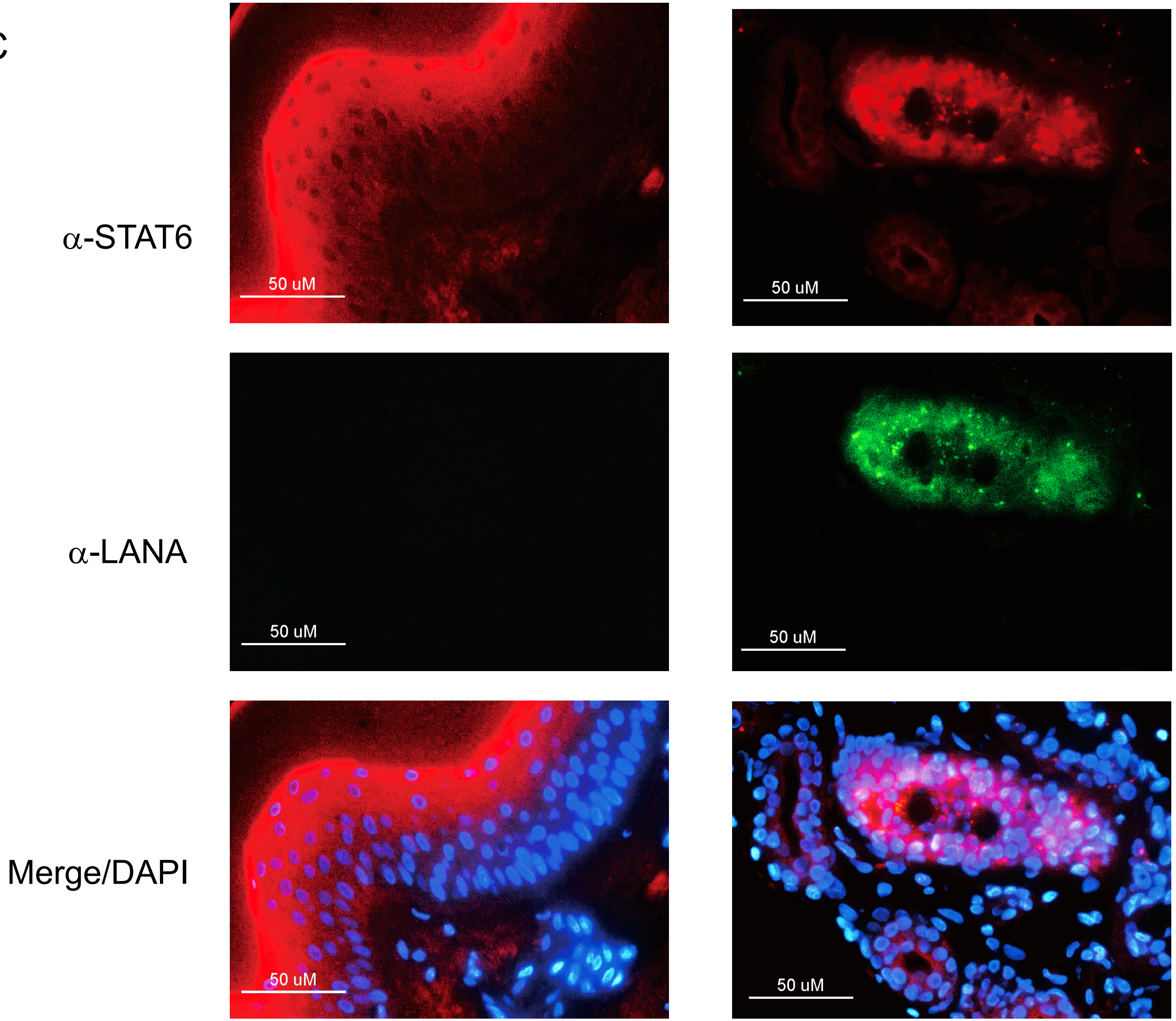

Figure 2A

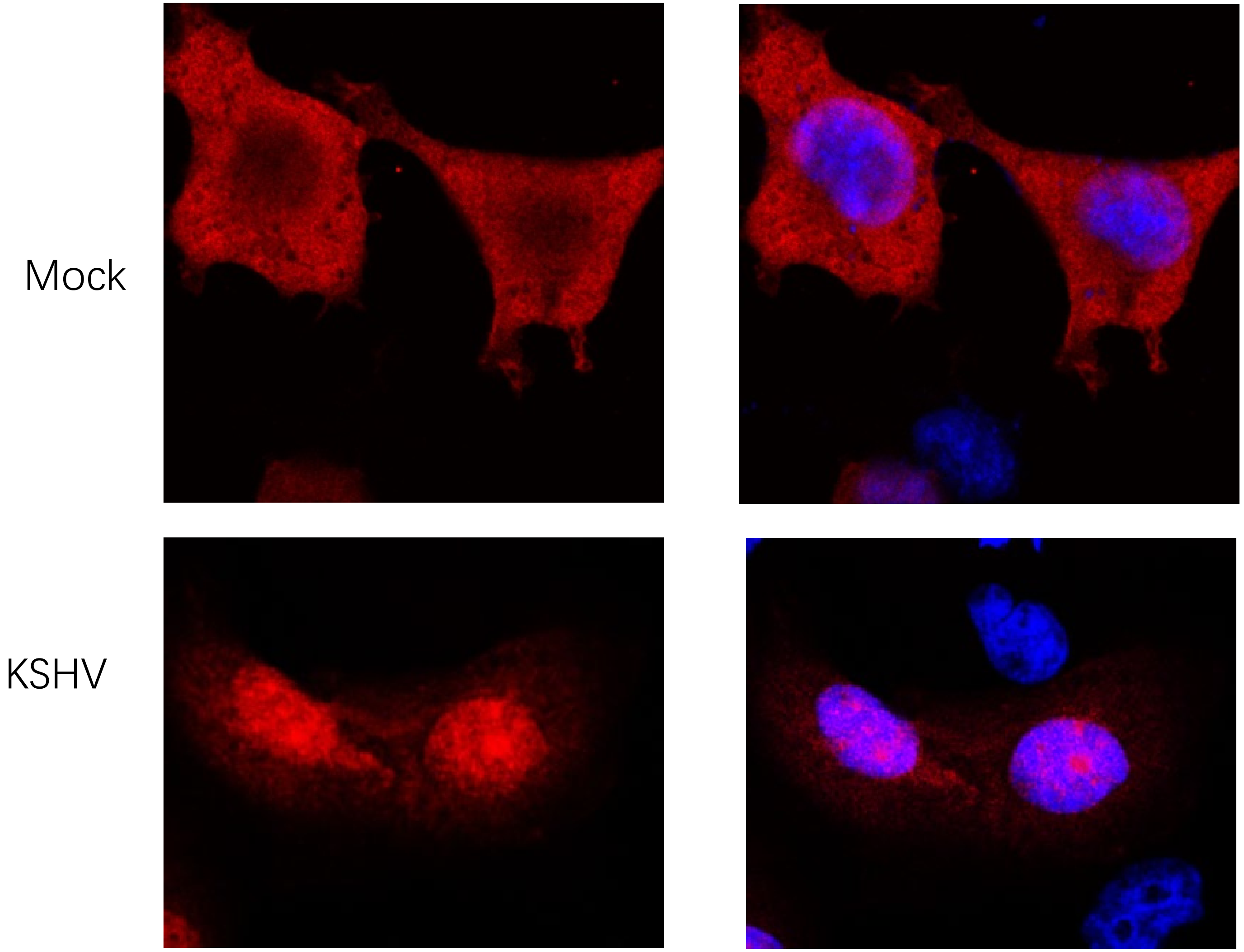

Figure 2B

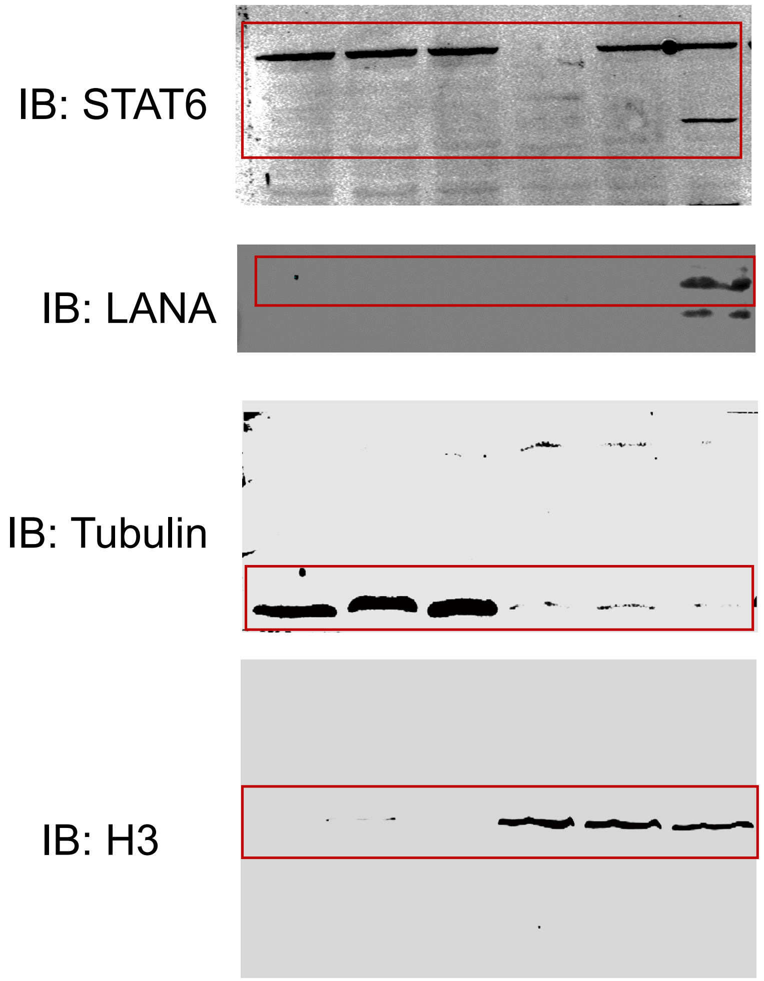

Figure 2C

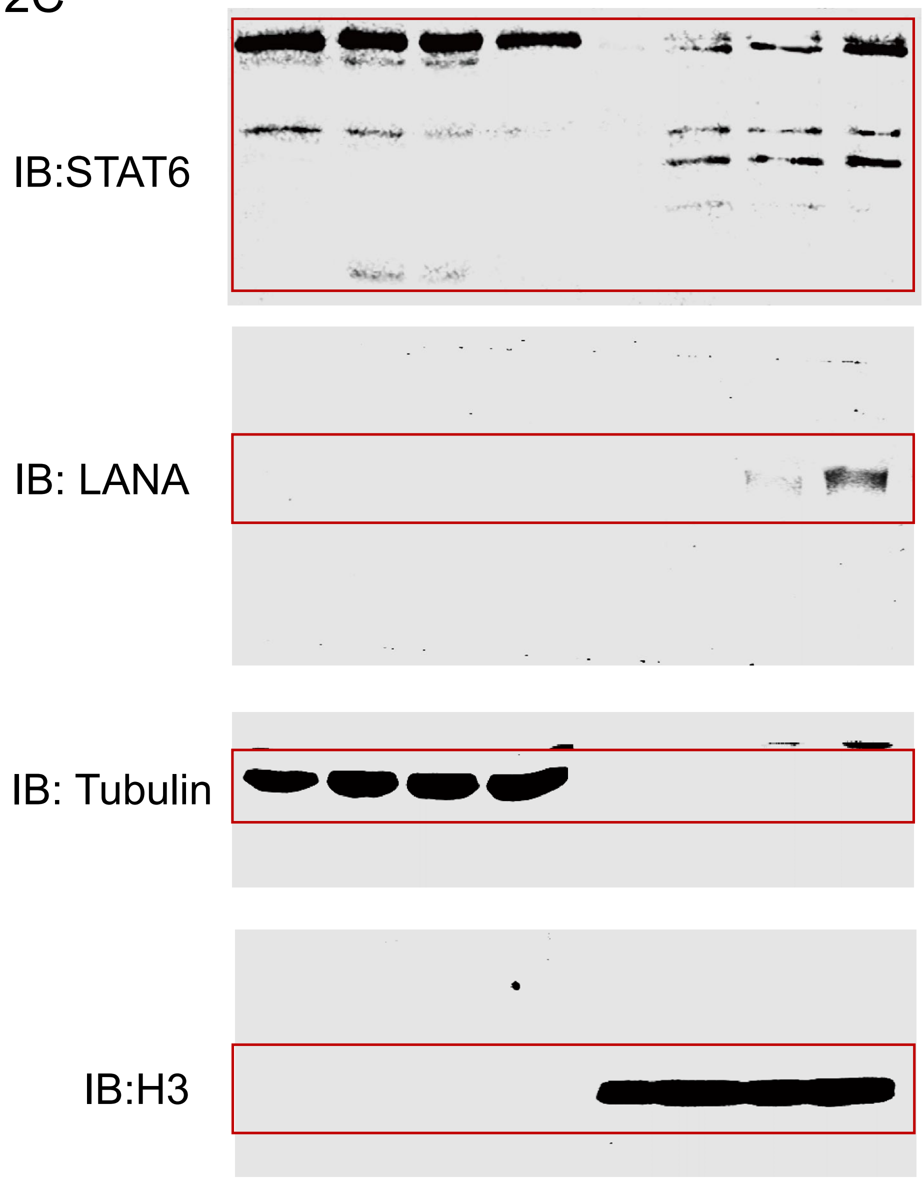

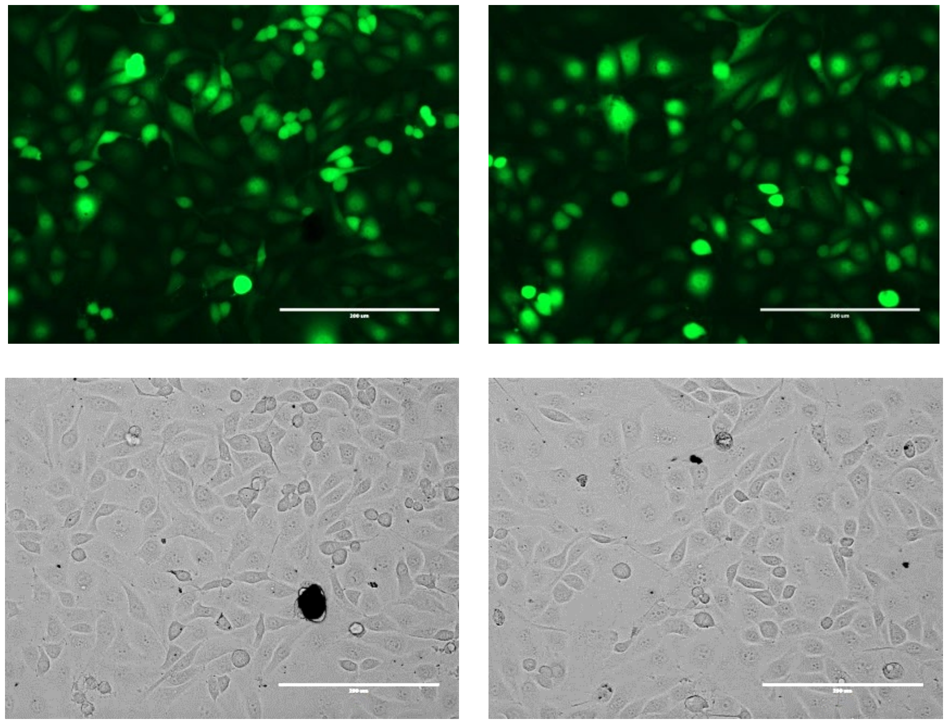

HUVEC/GFP-KSHV

Figure 3A

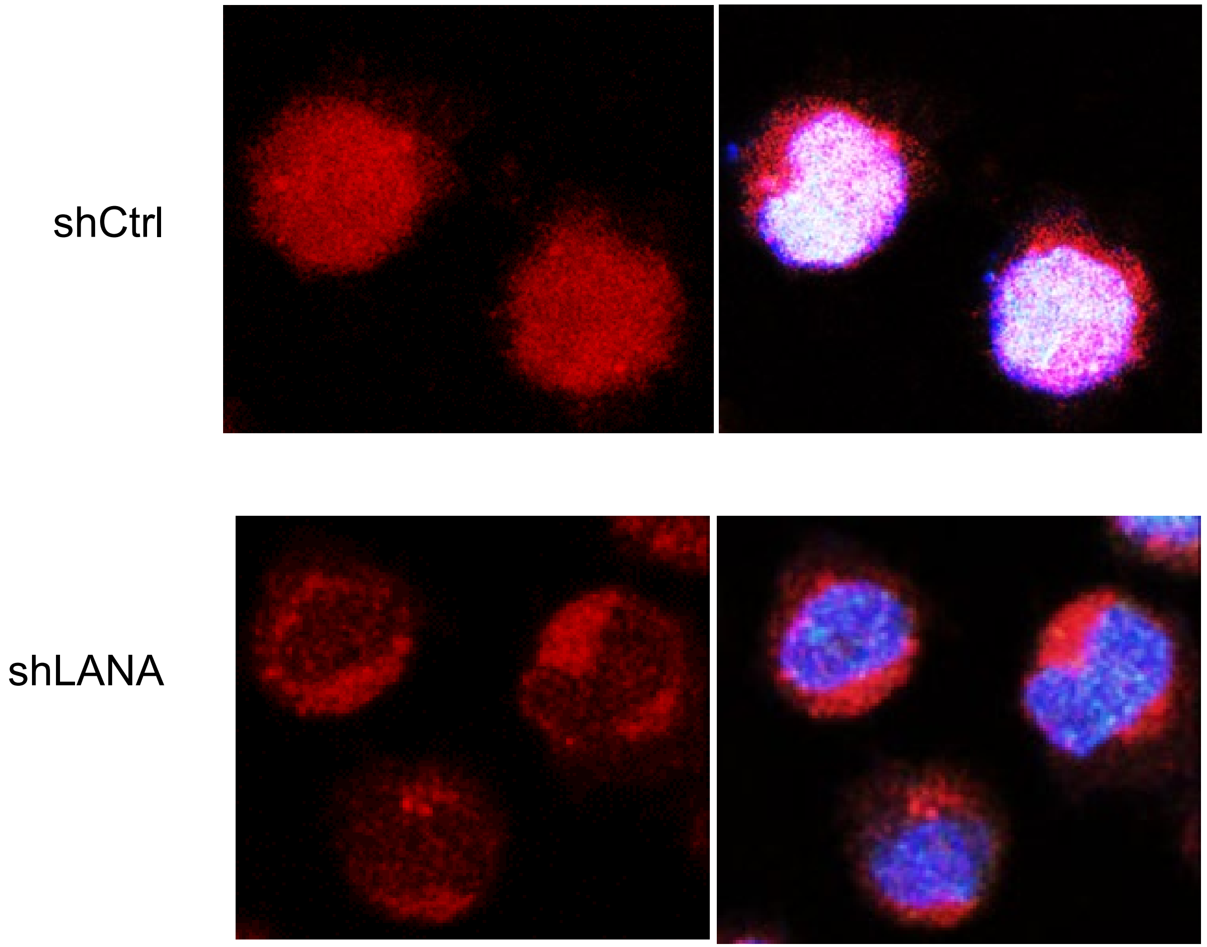

Figure 3B

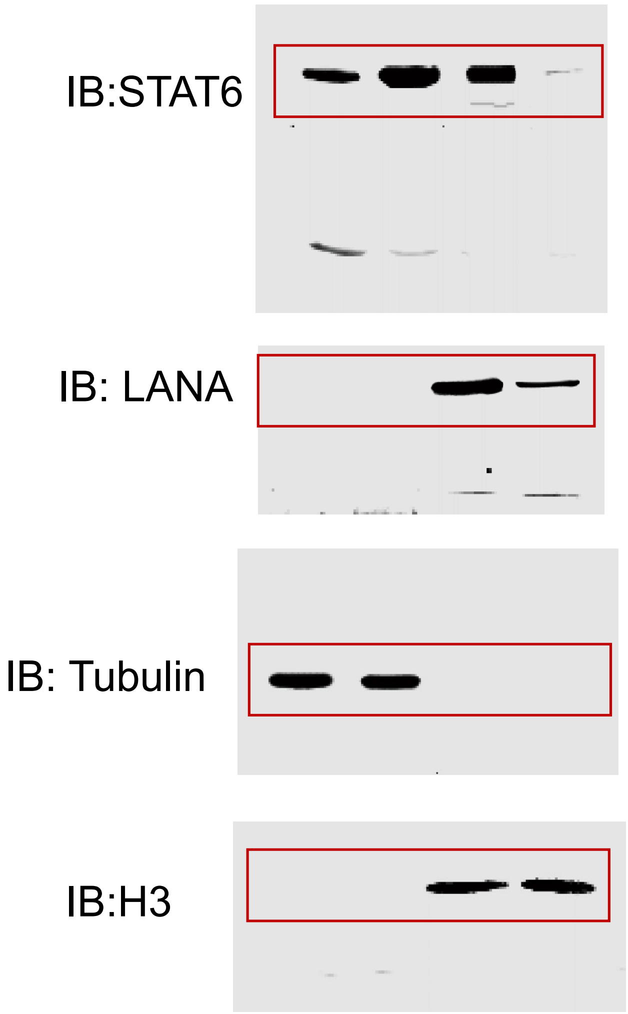

Figure 3C

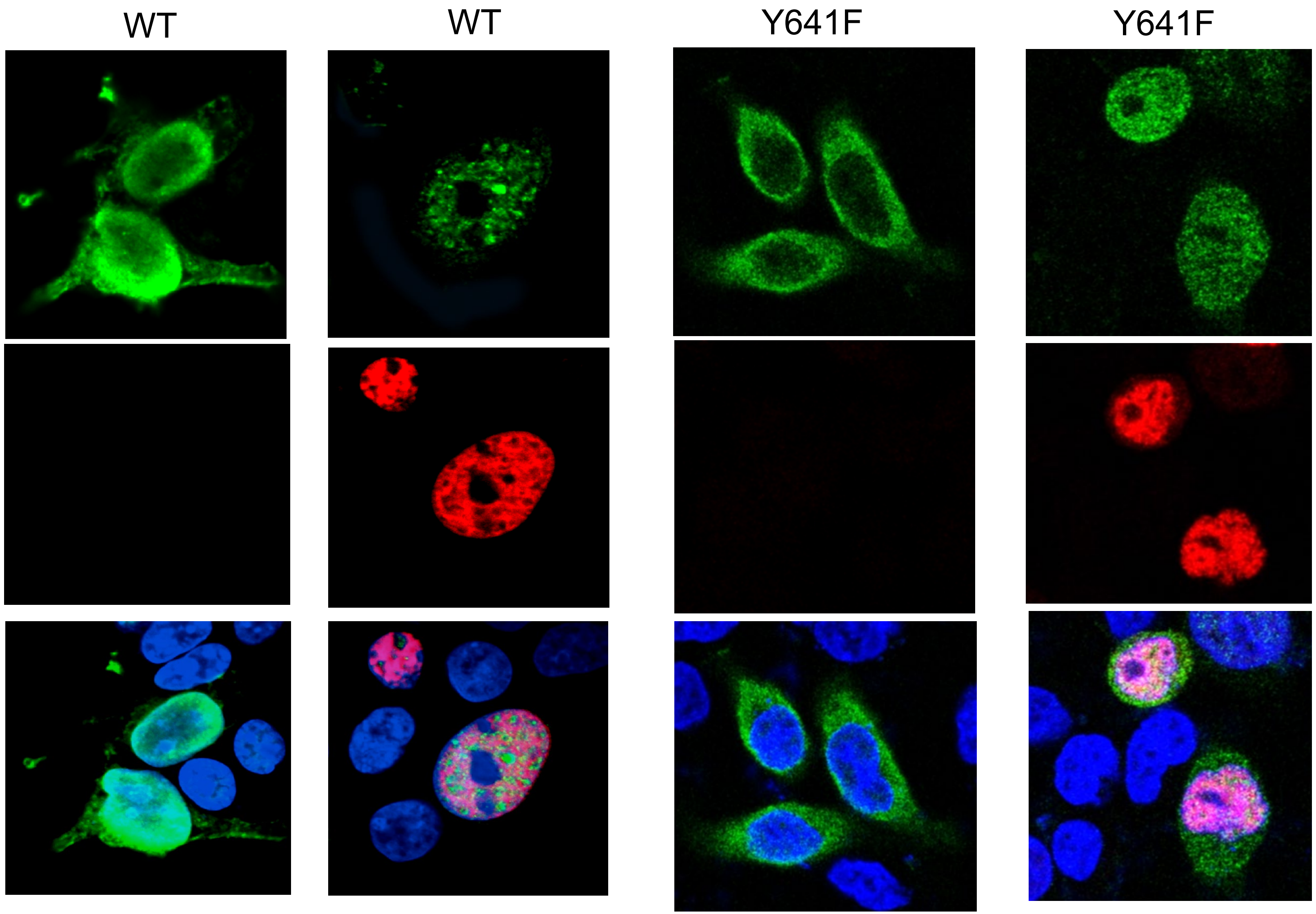

Figure 3D

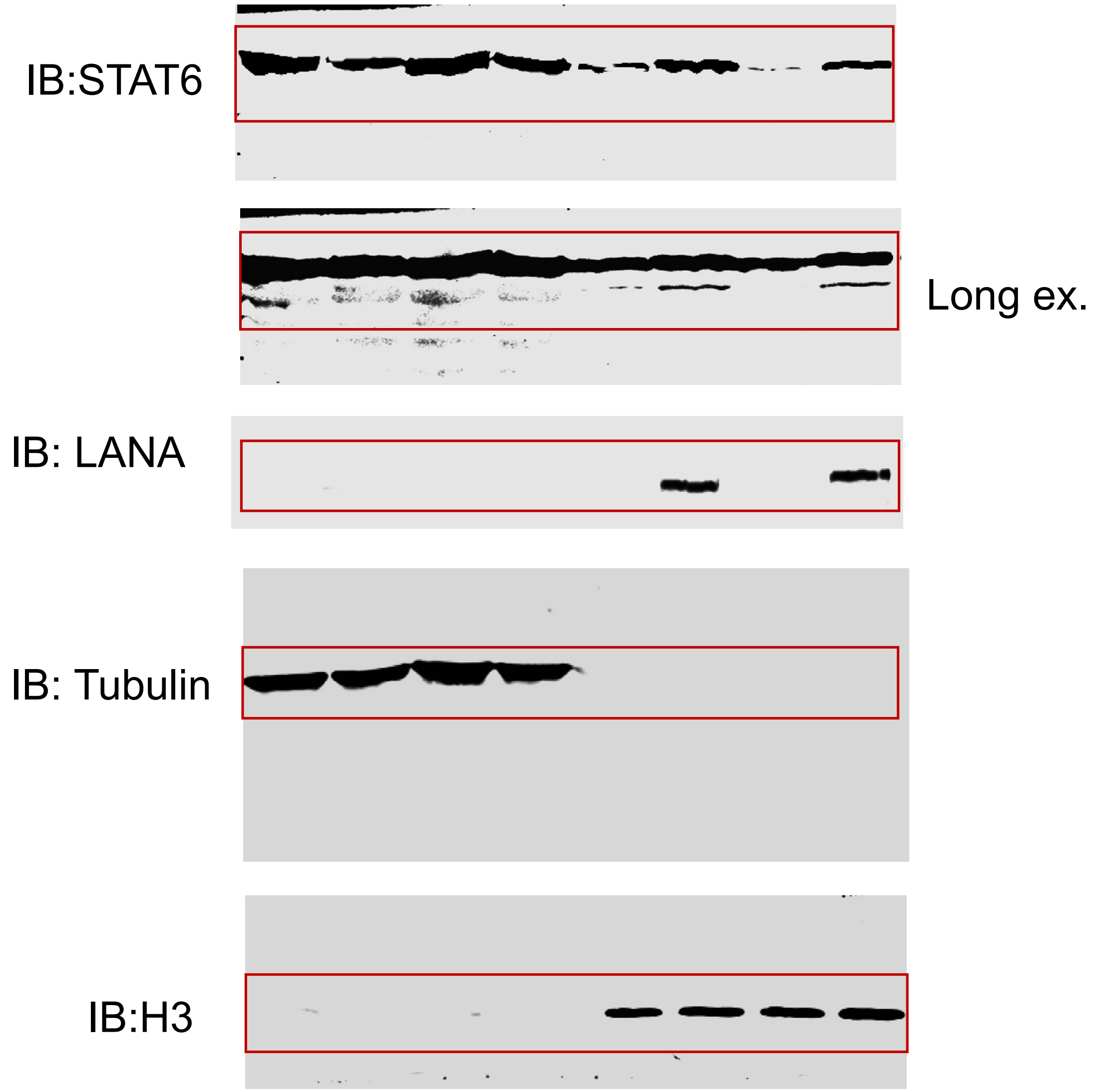

Figure 3E

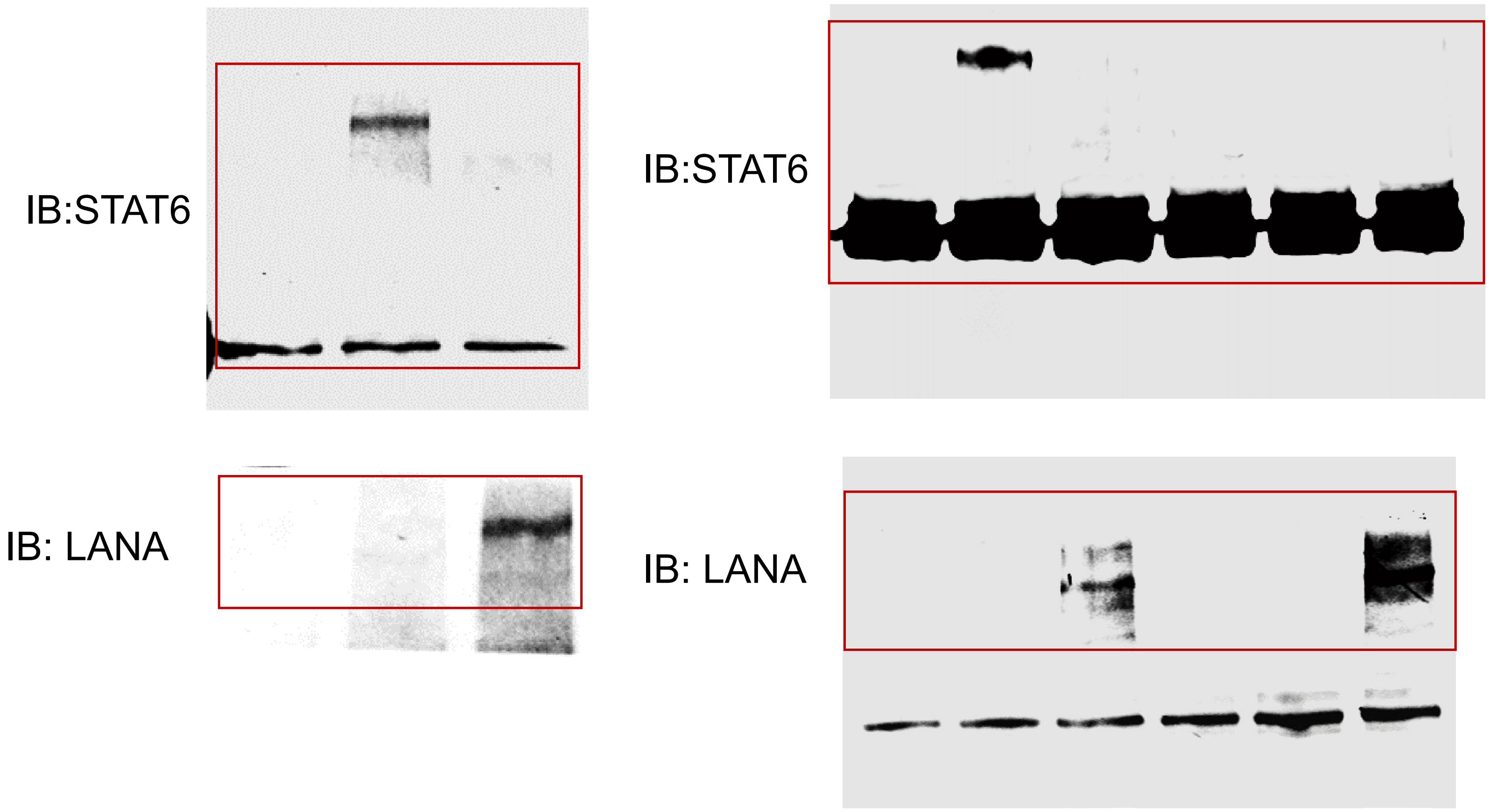

Figure 4A

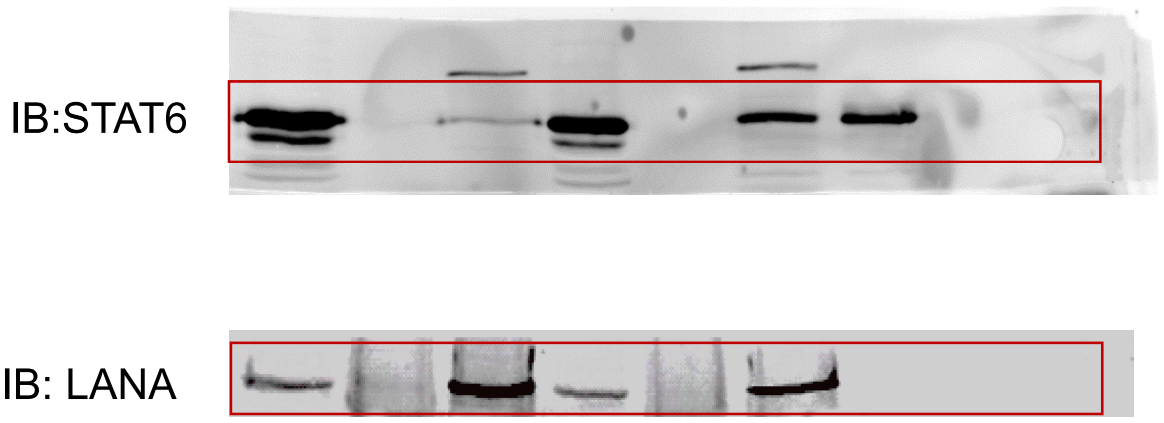

Figure 4B

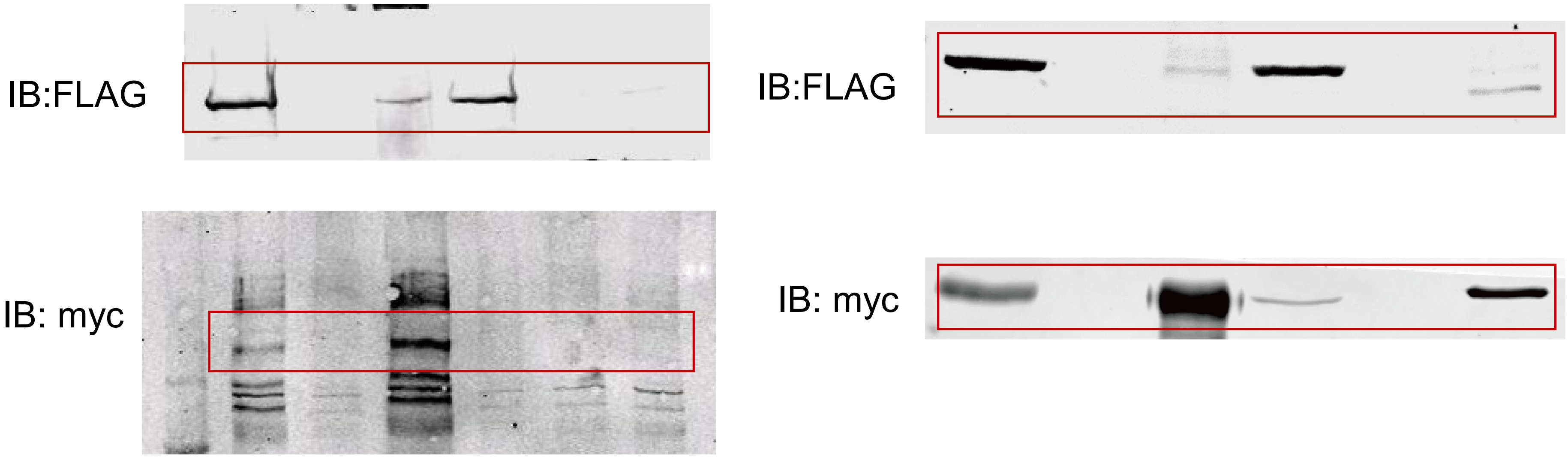

Figure 4C

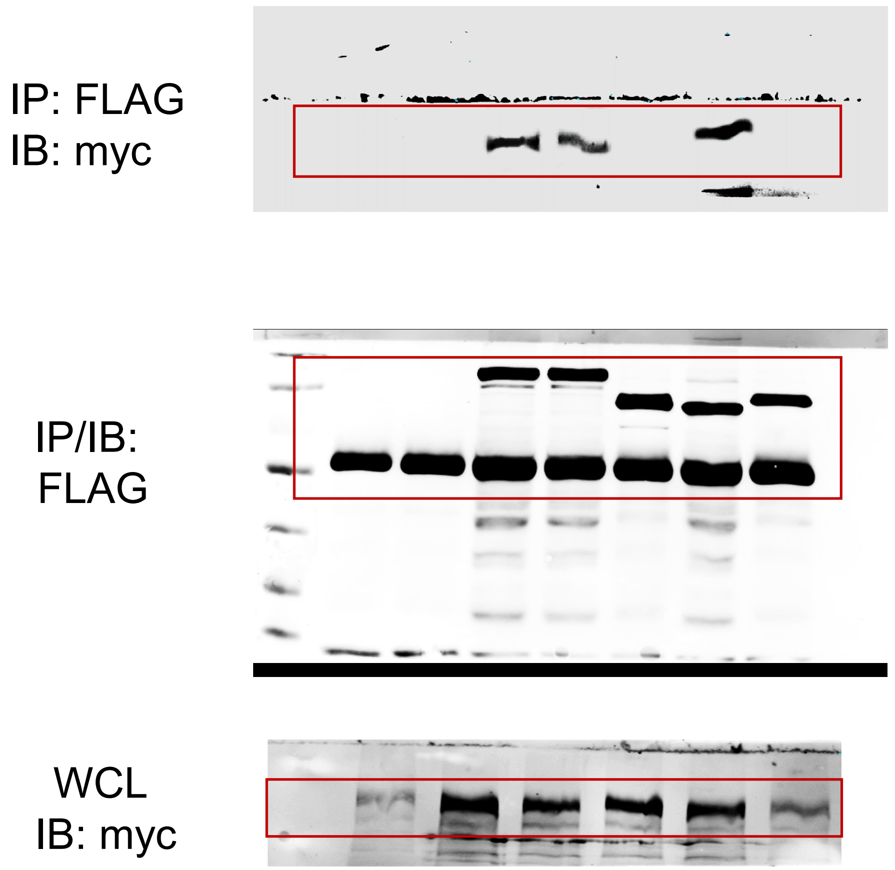

Figure 5A

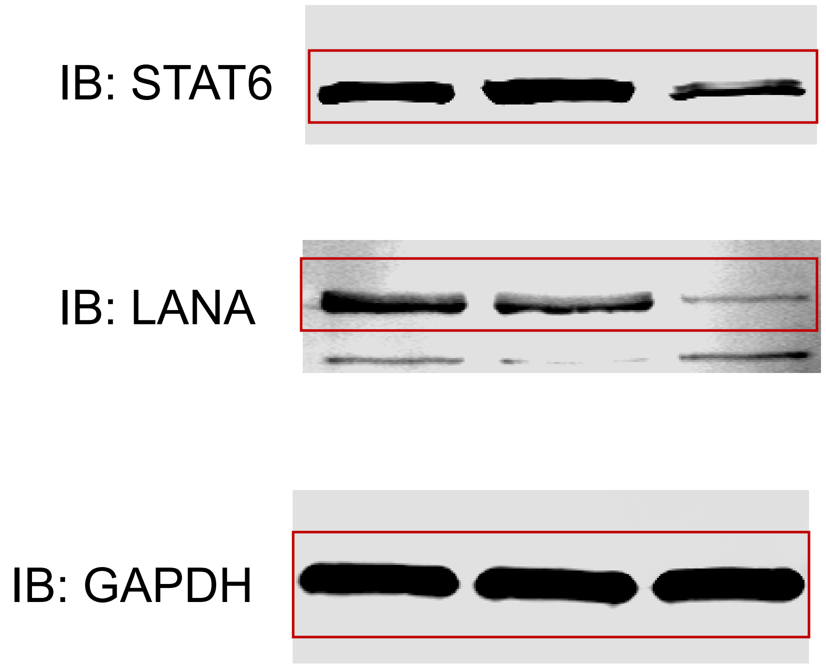

Figure 5B

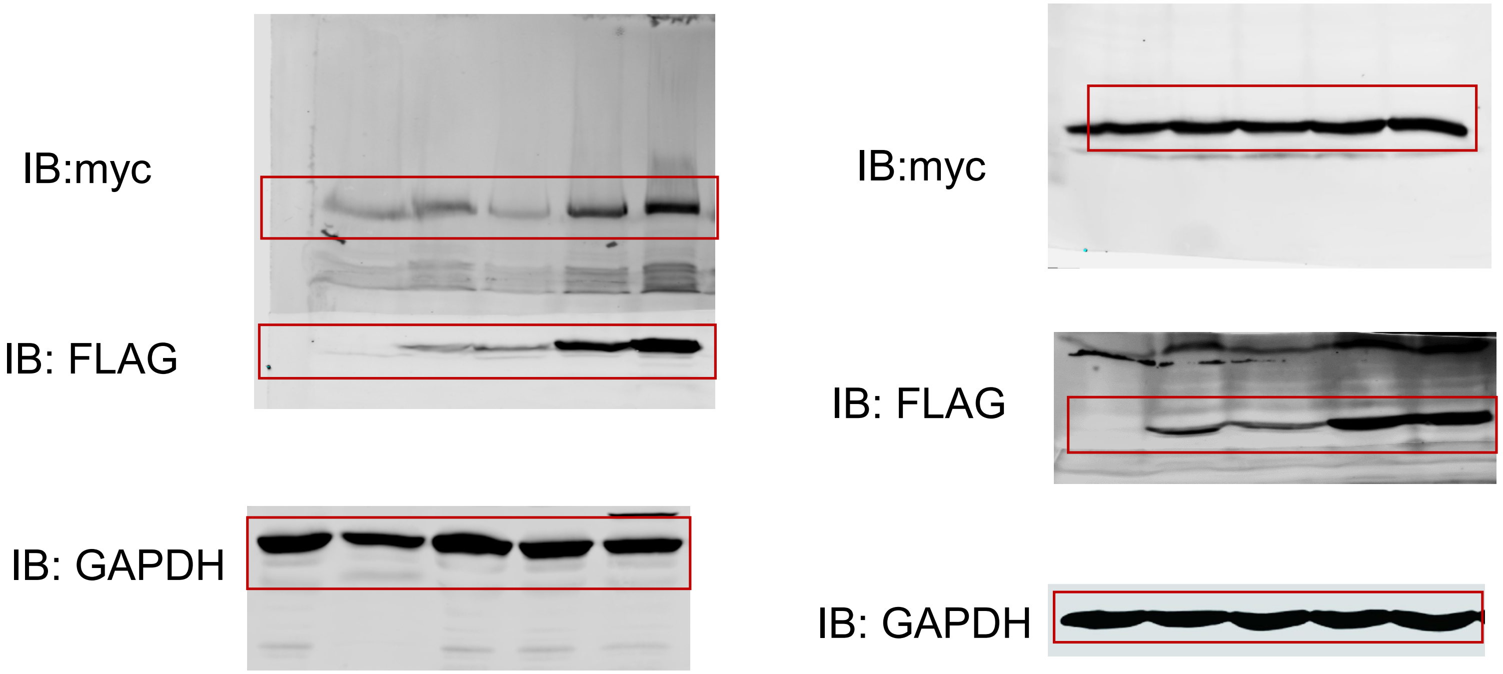

Figure 5C

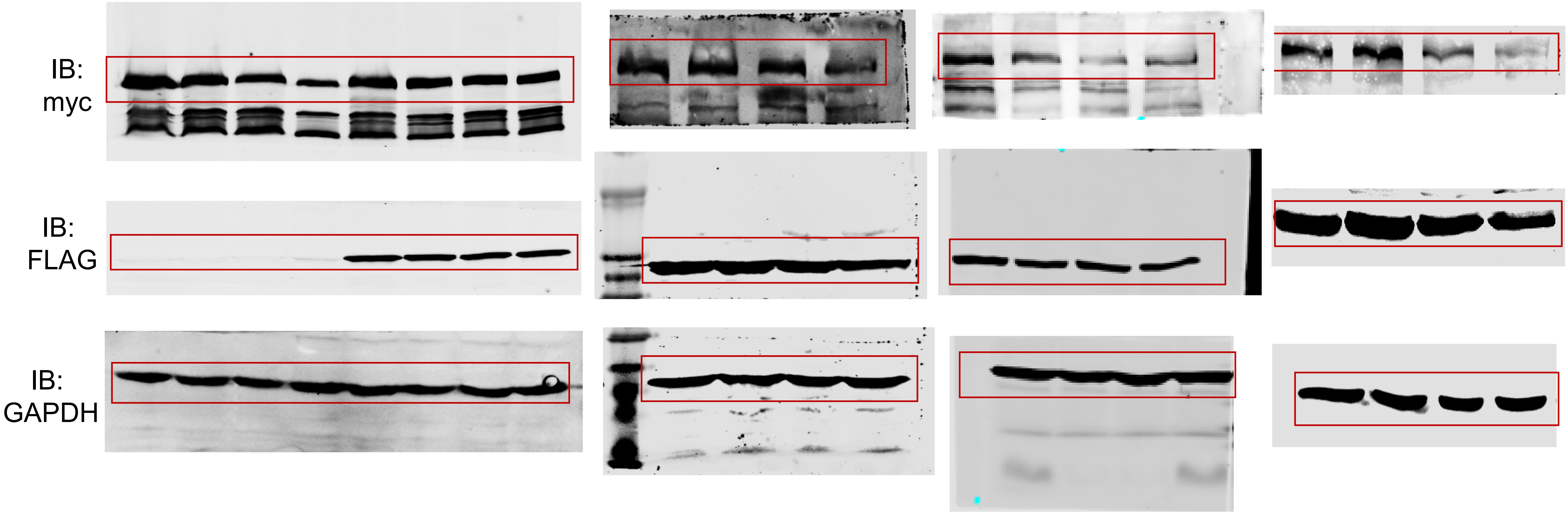

Figure 6A

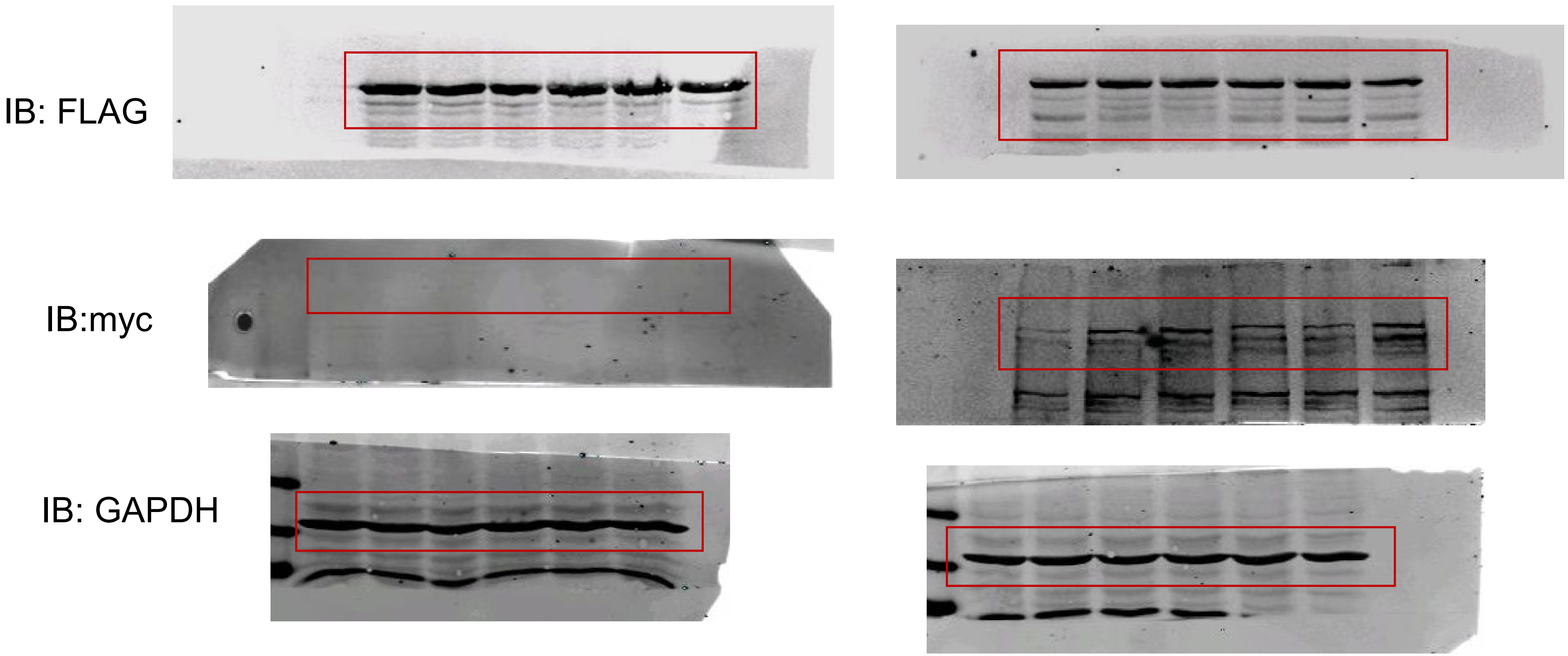

Figure 6B

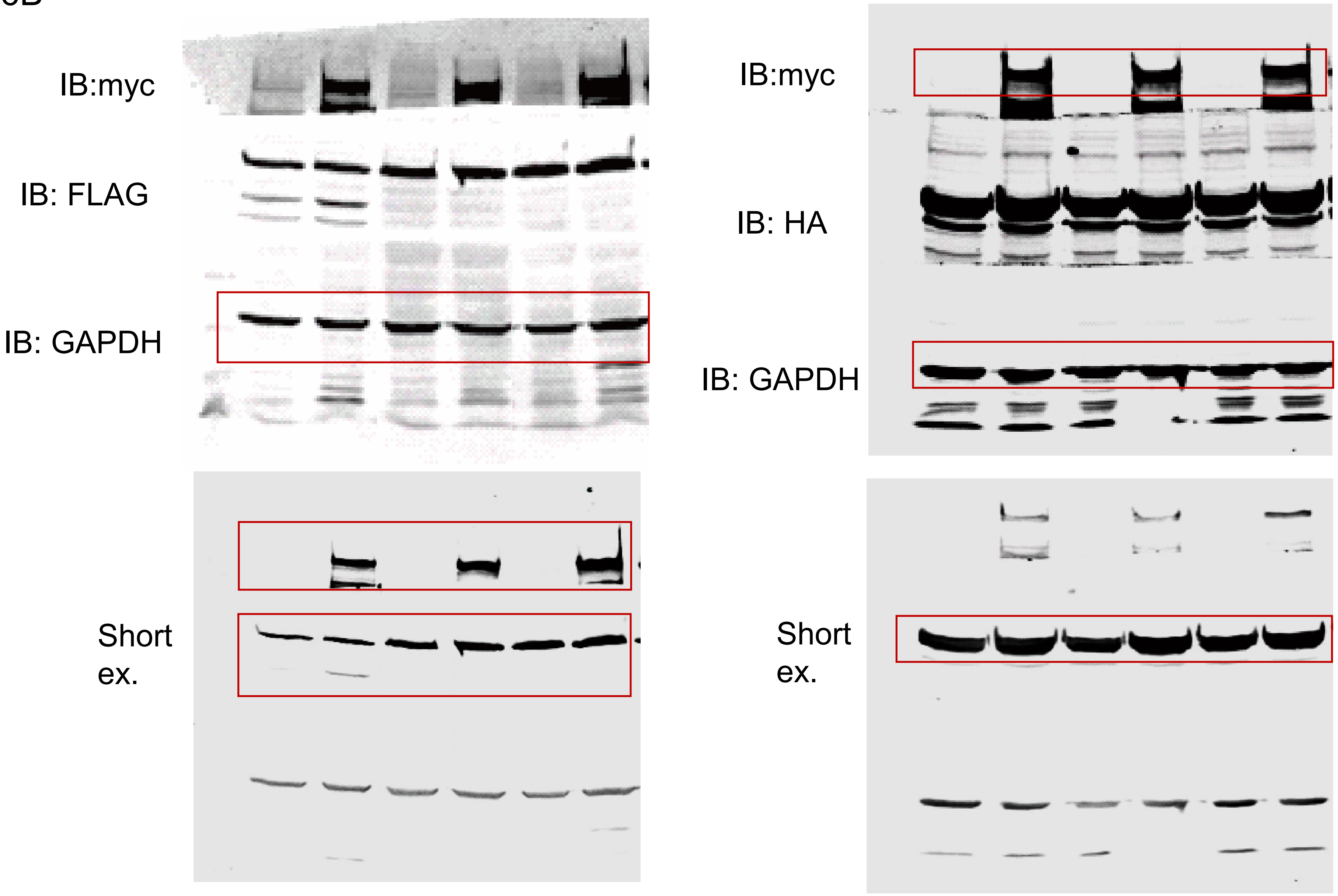

Figure 6C

IB: FLAG

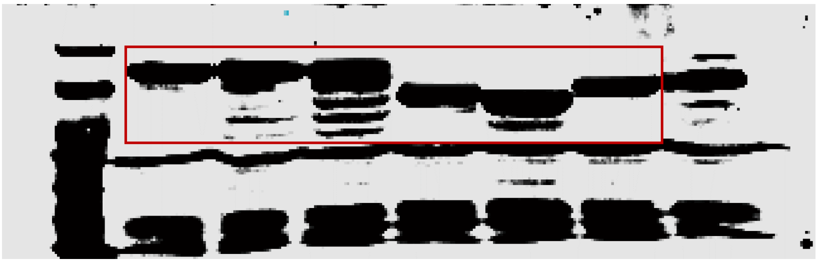

IB: myc

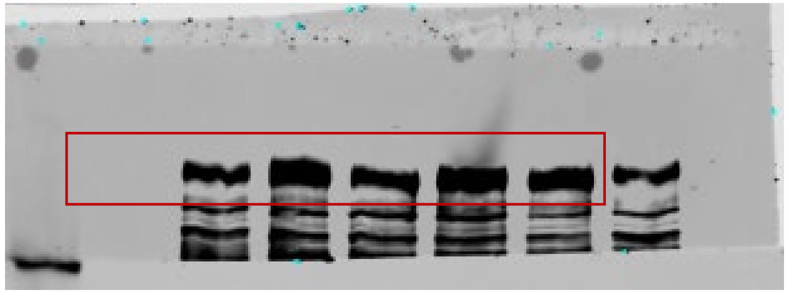

Figure 6D

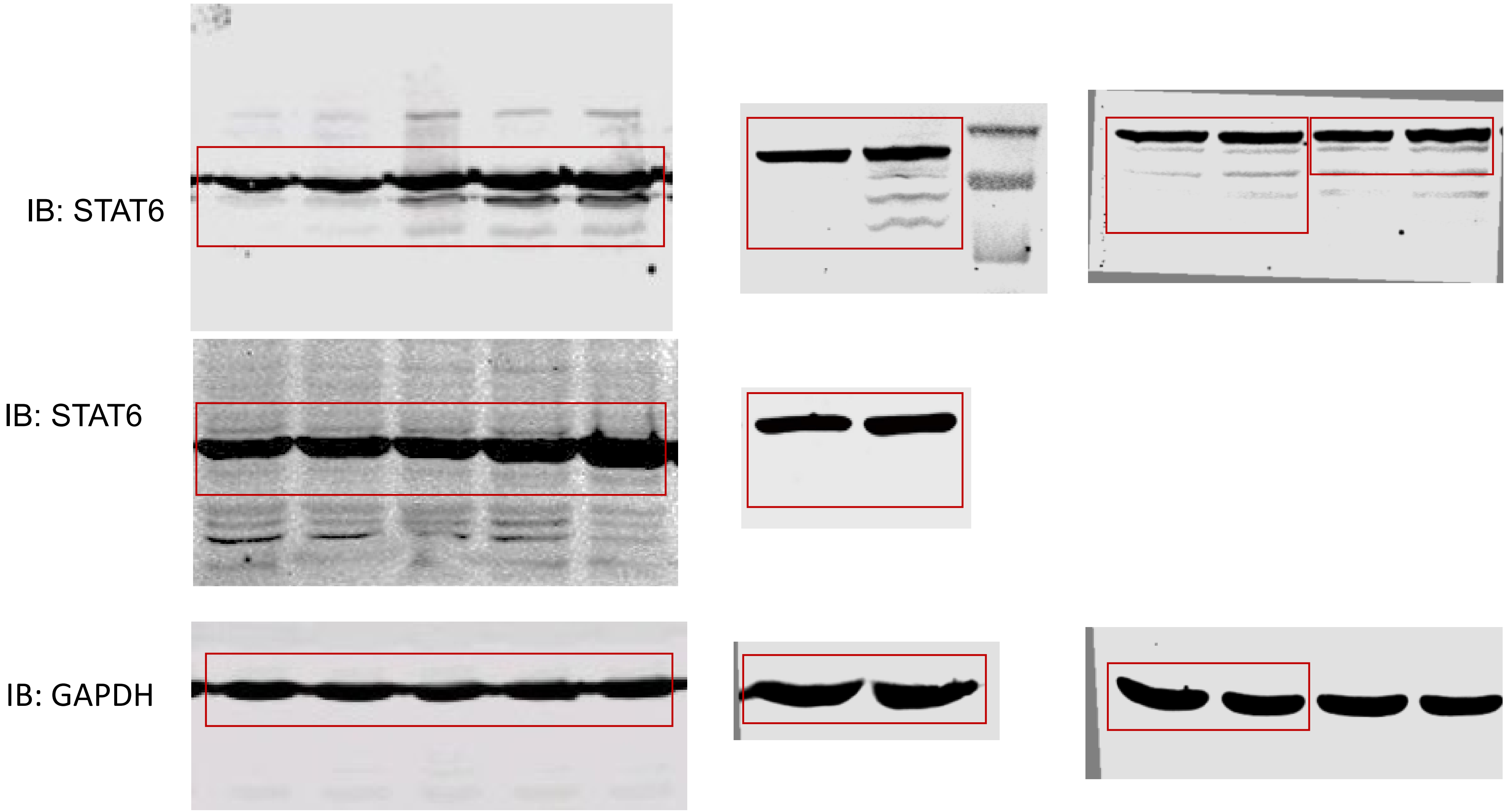

Figure 7D

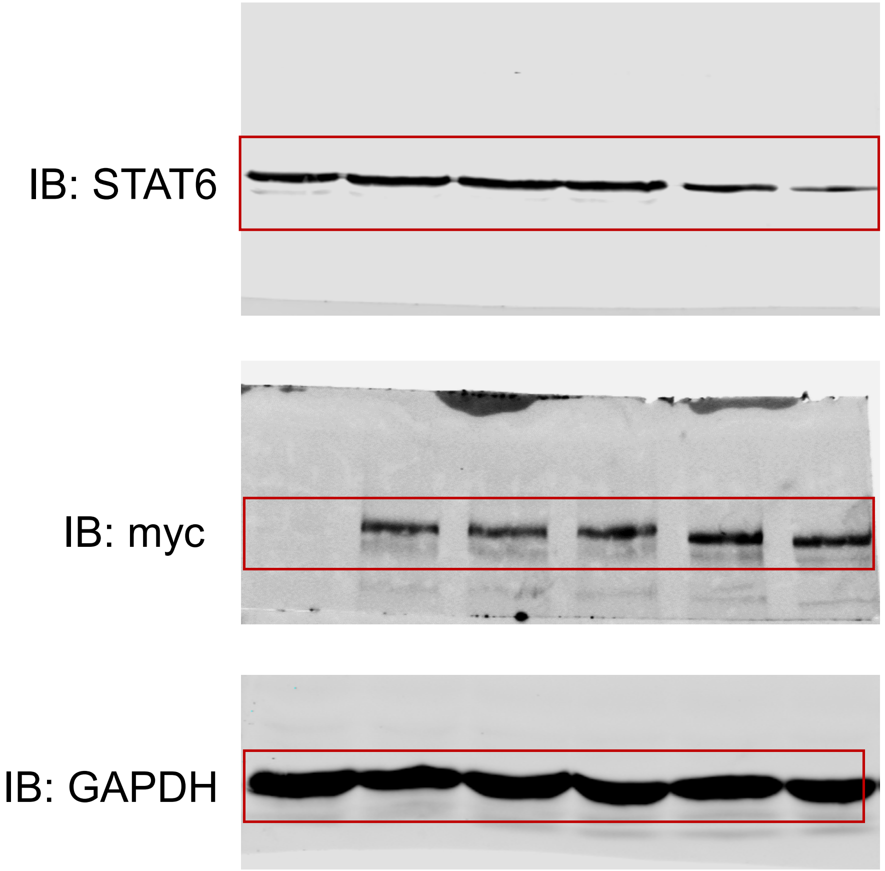

Figure 8C

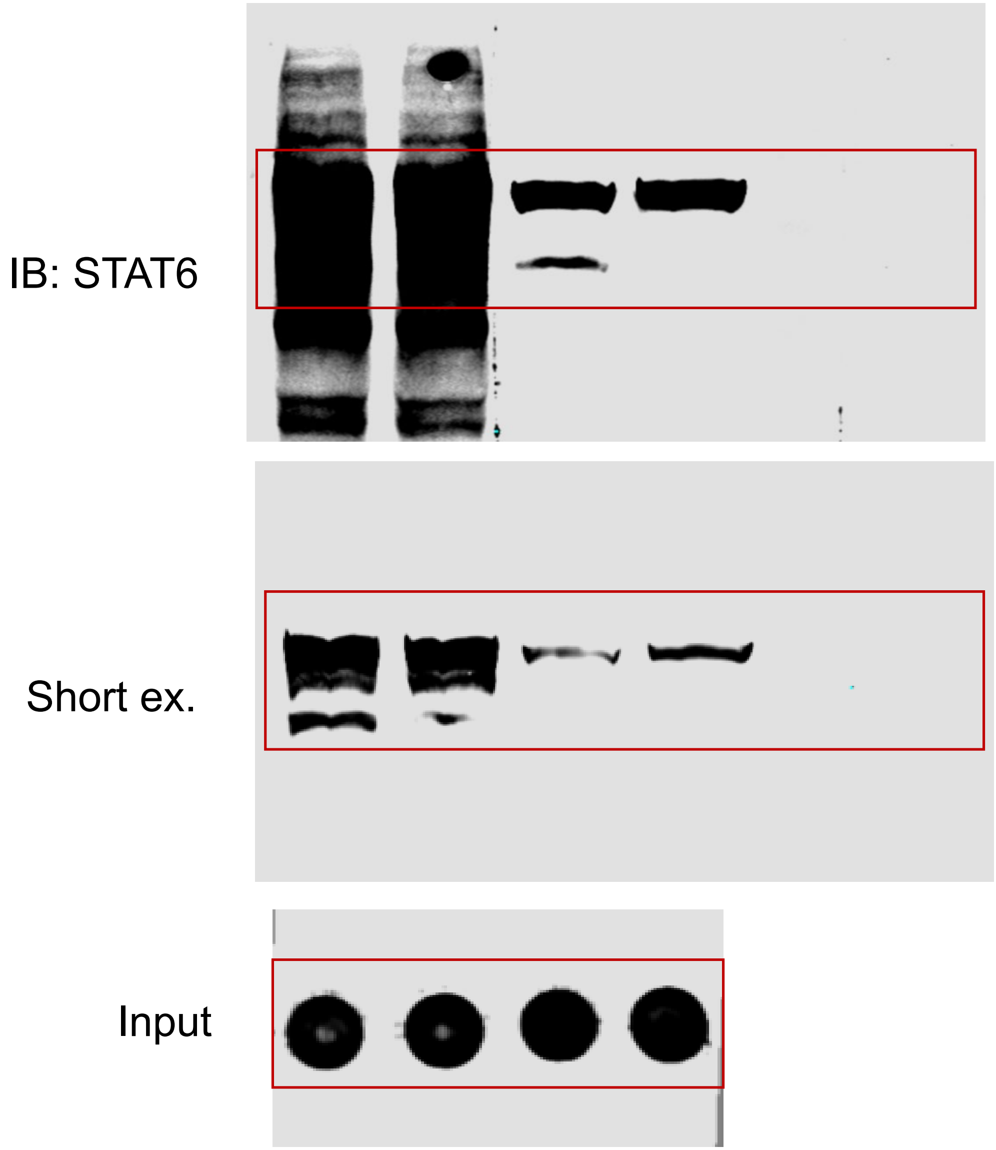

Figure 9A

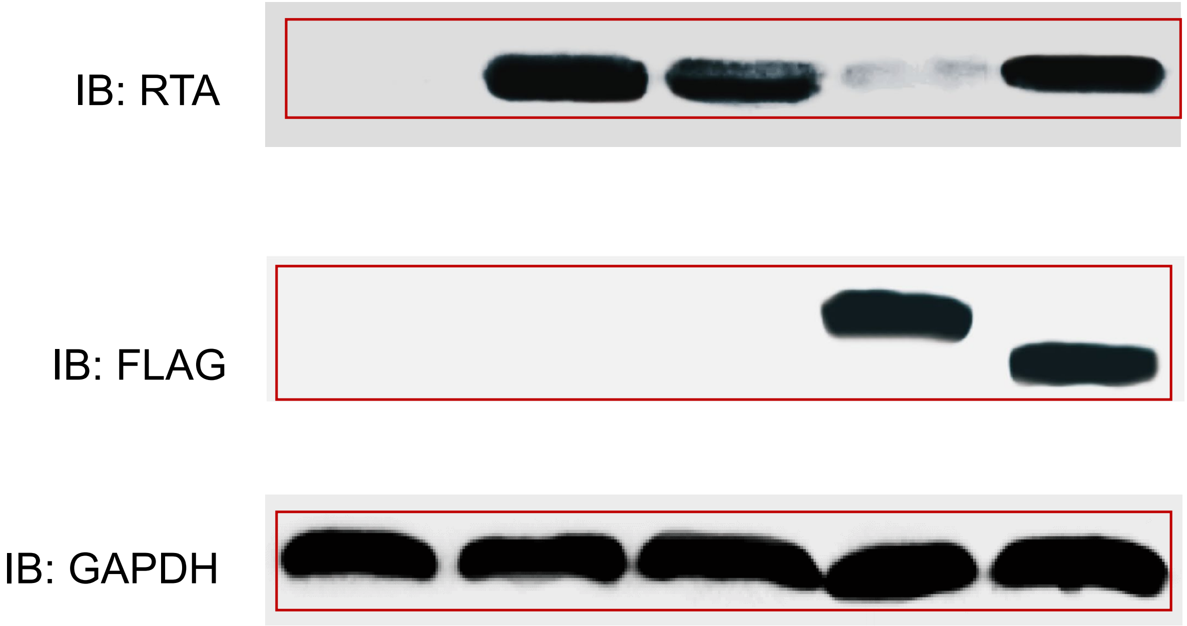

Figure 9B

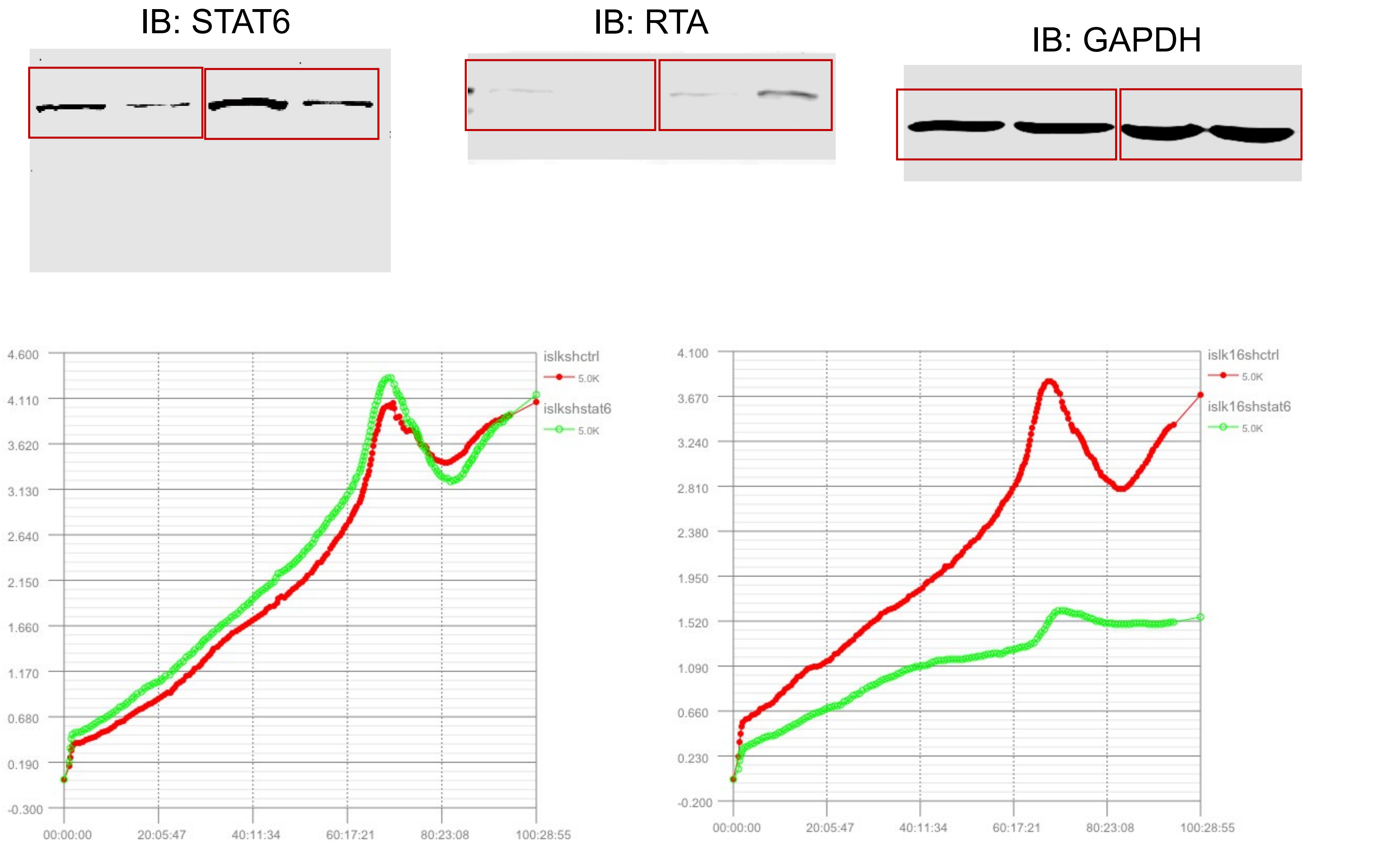

Cell growth curves were real-time recorded by Xcelligence RTCA machine

Figure 9C

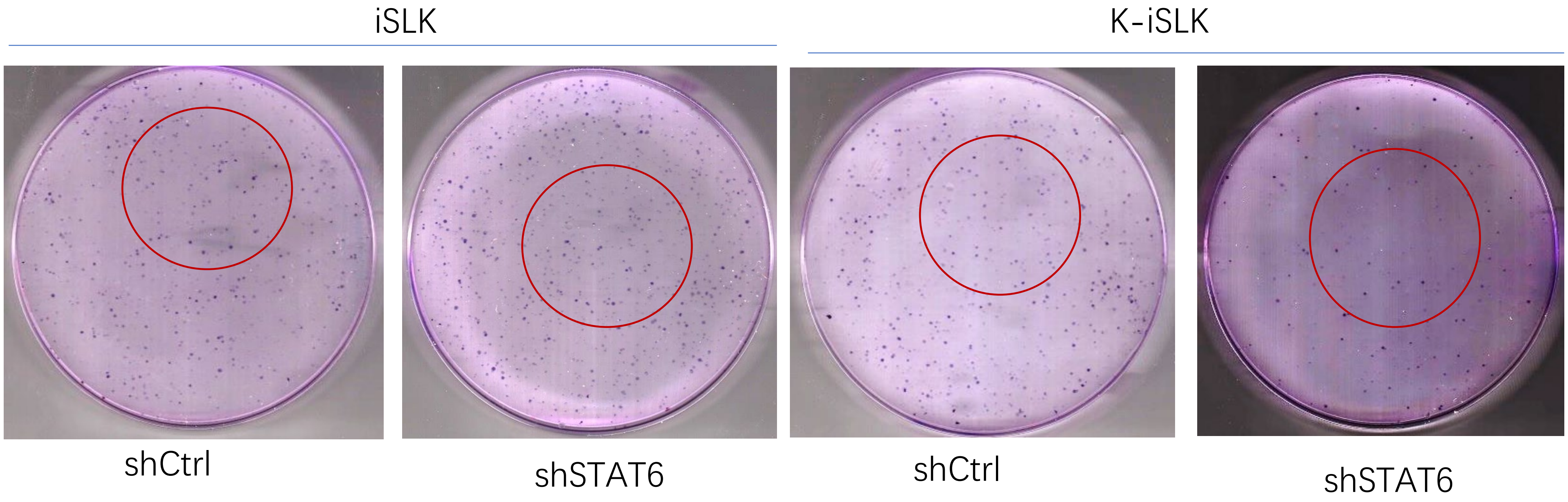

Supplement: S7 File — (PDF) [file ppat.1010047.s008.pdf]
